# Supplementary material for: Clinical signs of possible serious infection and associated mortality among young infants presenting at first-level health facilities
Source: PLoS One. 2021 Jun 30;16(6):e0253110. doi: 10.1371/journal.pone.0253110 (PMC8244884; doi:10.1371/journal.pone.0253110)
Supplement: S1 Table — (DOCX) [file pone.0253110.s001.docx]

**S1 Table. Case fatality ratio (CFR) with the place of treatment by study site and by IMCI* classification and recategorisation of signs of clinical severe infection or severe pneumonia on mortality risk (n = 7046)**

|  | **DRC (n=1253)** | | **Kenya (n=2269)** | | **Ibadan, Nigeria (n=1164)** | | **Ile-Ife, Nigeria (n=1039)** | | **Zaria, Nigeria (n=1321)** | |
| --- | --- | --- | --- | --- | --- | --- | --- | --- | --- | --- |
|  | **Outpatient treatment** | **Inpatient treatment** | **Outpatient treatment** | **Inpatient treatment** | **Outpatient treatment** | **inpatient treatment** | **outpatient treatment** | **inpatient treatment** | **Outpatient treatment** | **inpatient treatment** |
|  | **Deaths/ infants (CFR**^†^**)** | **Deaths/ infants (CFR**^†^**)** | **Deaths/ infants (CFR**^†^**)** | **Deaths/ infants (CFR**^†^**)** | **Deaths/ infants (CFR**^†^**)** | **Deaths/ infants (CFR**^†^**)** | **Deaths/ infants (CFR**^†^**)** | **Deaths/ infants (CFR**^†^**)** | **Deaths/ infants (CFR**^†^**)** | **Deaths/ infants (CFR**^†^**)** |
| **A. IMCI* classification** |  |  |  |  |  |  |  |  |  |  |
| Pneumonia^‡^ | 0/284 (0.0%) | 0/8 (0.0%) | 1/711 (0.1%) | 1/11 (9.1%) | 0/168 (0.0%) | 0/64 (0.0%) | 0/173 (0.0%) | 0/0 | 2/165 (1.2%) | 0/259 (0.0%) |
| Clinical severe infection^§^ or severe pneumonia^¶^ | 26/837 (3.1%) | 7/50 (14.0%) | 17/1510 (1.1%) | 2/18 (11.1%) | 7/800 (0.9%) | 2/96 (2.1%) | 4/824 (0.5%) | 1/37 (2.7%) | 34/714 (4.8%) | 11/151 (7.3%) |
| Critical illness^#^ | 11/41 (26.8%) | 8/33 (24.2%) | 3/16 (18.7%) | 2/3 (66.7%) | 1/3 (33.3%) | 0/2 (0.0%) | 0/5 (0.0%) | 0/0 | 3/18 (16.7%) | 0/14 (0.0%) |
|  |  |  |  |  |  |  |  |  |  |  |
| **B. Recategorisation of signs of clinical severe infection**^§^ **or severe pneumonia**^¶^  **based on mortality risk** |  |  |  |  |  |  |  |  |  |  |
| **Low-mortality risk signs^**^** | **12/677 (1.8%)** | **4/31 (12.9%)** | **8/1120 (0.7%)** | **1/13 (7.7%)** | **6/686 (0.9%)** | **0/87 (0.0%)** | **4/775 (0.5%)** | **1/36 (2.8%)** | **7/468 (1.5%)** | **6/136 (4.4%)** |
| High body temperature^††^ | 3/338 (0.9%) | 0/2 (0.0%)) | 1/300 (0.3%) | 0/4 (0.0%)- | 2/339 (0.6%) | 0/11 (0.0%) | 1/202 (0.5%) | 0/6 (0.0%) | 4/204 (2.0%) | 0/3 (0.0%) |
| Severe chest indrawing^††^ | 4/113 (3.5%) | 1/2 (50.0%) | 2/526 (0.4%) | 0/2 (0.0%) | 2/181 (1.1%) | 0/6 (0.0%) | 1/403 (0.2%) | 1/9 (11.1%) | 1/87 (1.1%) | 0/0 |
| Only fast breathing in 0-6 days old^††^ | 5/226 (2.2%) | 3/27 (11.1%) | 5/294 (1.7%) | 1/7 (14.3%) | 2/166 (1.2%) | 0/70 (0.0%) | 2/170 (1.2%) | 0/21 (0.0%) | 2/177 (1.1%) | 6/133 (4.5%) |
| **Moderate-mortality risk signs^‡‡^** | **14/160 (8.7%)** | **3/19 (15.8%)** | **9/390 (2.3%)** | **1/5 (20.0%)** | **1/114 (0.9%)** | **2/9 (22.2%)** | **0/49 (0.0%)** | **0/1 (0.0%)** | **27/246 (11.0%)** | **5/15 (33.3%)** |
| Movement only when stimulated^††^ | 0/3 (0.0%) | 0/0 | 1/6 (16.7%) | 0/0 | 0/9 (0.0%) | 0/1 (0.0%) | 0/0 | 0/0 | 0/12 (0.0%) | 0/0 |
| Stopped feeding well^††^ | 2/38 (5.3%) | 1/3 (33.3%) | 4/87 (4.6%) | 0/0 | 0/32 (0.0%) | 0/3 (0.0%) | 0/4 (0.0%) | 0/0 | 3/82 (3.7%) | 0/2 (0.0%) |
| Low body temperature^††^ | 3/36 (8.3%) | 1/11 (9.1%) | 1/55 (1.8%) | 1/3 (33.3%) | 0/7 (0.0%) | 1/1 (100.0%) | 0/7 (0.0%) | 0/0 | 11/56 (19.6%) | 2/6 (33.3%) |
| Multiple signs of clinical severe infection^§^ | 9/83 (10.8%) | 1/5 (20.0%) | 3/390 (2.3%) | 0/2 (0.0%) | 1/66 (1.5%) | 1/4 (25.0%) | 0/38 (0.0%) | 0/1 (0.0%) | 13/96 (13.5%) | 3/7 (42.9%) |

*IMCI: Integrated management of childhood illnesses.

†CFR: Case fatality ratio.

‡Pneumonia is defined as fast breathing (respiratory rate of ≥ 60 breaths per minute) in 7-59 days old infants.

§Clinical severe infection is defined as the presence of any one of the following signs: severe chest indrawing, high body temperature (≥ 38 ^o^C), stopped feeding well, movement only when stimulated, or low body temperature (< 35.5 ^o^C).

¶ Severe pneumonia is defined as fast breathing (respiratory rate of ≥ 60 breaths per minute) in 0-6 days old infants.

^#^Critical illness is defined as the presence of any one of the following signs: convulsions, unable to feed at all, or no movement at all. For the current analysis, only these three common signs of critical illness were considered.

^**^ Low-mortality risk signs are defined as infants with a case fatality ratio for any sign < 2.0%.

†† Young infant presenting with a single sign.

‡‡ Moderate-mortality risk signs are defined as infants with a case fatality ratio for any sign >2.0%.
