# Supplementary material for: Clinical signs of possible serious infection and associated mortality among young infants presenting at first-level health facilities
Source: PLoS One. 2021 Jun 30;16(6):e0253110. doi: 10.1371/journal.pone.0253110 (PMC8244884; doi:10.1371/journal.pone.0253110)
Supplement: S1 File — (PDF) [file pone.0253110.s002.pdf]

siteid	age	sex	classification	place
Kenya	6	female	>1 CSI	OPD
Kenya	4	female	fever	OPD
Kenya	4	female	fever	OPD
Kenya	3	female	Hypo	OPD
Kenya	2	female	LCI	OPD
Kenya	2	Male	LCI	OPD
Kenya	4	Male	poor feeding	OPD
Kenya	6	Male	fever	OPD
Kenya	4	female	>1 CSI	OPD
Kenya	4	female	poor feeding	OPD
Kenya	5	female	LCI	OPD
Kenya	3	female	fever	OPD
Kenya	5	female	Hypo	OPD
Kenya	2	female	>1 CSI	OPD
Kenya	4	Male	LCI	OPD
Kenya	3	Male	Hypo	OPD
Kenya	3	Male	LCI	OPD
Kenya	1	Male	LCI	OPD
Kenya	5	Male	LCI	OPD
Kenya	5	female	poor feeding	OPD
Kenya	4	female	fever	OPD
Kenya	4	Male	LCI	OPD
Kenya	6	Male	fever	OPD
Kenya	3	female	LCI	OPD
Kenya	3	female	poor feeding	OPD
Kenya	3	Male	poor feeding	OPD
Kenya	2	Male	fever	OPD
Kenya	1	female	Hypo	OPD
Kenya	4	female	>1 CSI	OPD
Kenya	4	Male	poor feeding	OPD
Kenya	3	Male	>1 CSI	OPD
Kenya	3	Male	fever	OPD
Kenya	1	Male	Hypo	OPD
Kenya	4	Male	LCI	OPD
Kenya	3	female	fever	OPD
Kenya	2	female	>1 CSI	OPD
Kenya	3	Male	Hypo	OPD
Kenya	1	female	>1 CSI	OPD
Kenya	3	female	fever	OPD
Kenya	1	Male	poor feeding	OPD
Kenya	3	female	>1 CSI	OPD
Kenya	4	Male	fever	OPD
Kenya	6	female	>1 CSI	OPD
Kenya	4	female	Hypo	OPD
Kenya	2	Male	>1 CSI	OPD
Kenya	6	female	fever	OPD
Kenya	2	Male	fever	OPD
Kenya	3	Male	poor feeding	OPD
Kenya	5	Male	>1 CSI	OPD

Kenya	1	Male	fever	OPD
Kenya	2	female	fever	OPD
Kenya	3	female	>1 CSI	OPD
Kenya	6	female	Hypo	OPD
Kenya	2	Male	fever	OPD
Kenya	2	female	fever	OPD
Kenya	3	female	fever	OPD
Kenya	3	Male	fever	OPD
Kenya	2	Male	fever	OPD
Kenya	3	Male	poor feeding	OPD
Kenya	4	Male	poor feeding	OPD
Kenya	4	Male	poor feeding	OPD
Kenya	2	female	fever	OPD
Kenya	1	Male	fever	OPD
Kenya	1	Male	Hypo	OPD
Kenya	2	female	fever	OPD
Kenya	4	Male	Hypo	OPD
Kenya	3	female	fever	OPD
Kenya	3	Male	fever	OPD
Kenya	1	female	Hypo	OPD
Kenya	6	Male	poor feeding	OPD
Kenya	3	Male	fever	OPD
Kenya	6	female	fever	OPD
Kenya	3	female	Hypo	OPD
Kenya	3	Male	>1 CSI	OPD
Kenya	3	Male	LCI	OPD
Kenya	1	female	LCI	OPD
Kenya	6	female	fever	OPD
Kenya	5	Male	LCI	OPD
Kenya	6	female	Hypo	OPD
Kenya	2	Male	>1 CSI	OPD
Kenya	3	female	LCI	OPD
Kenya	3	Male	LCI	OPD
Kenya	3	Male	fever	OPD
Kenya	6	female	fever	OPD
Kenya	2	female	Hypo	OPD
Kenya	2	Male	Hypo	OPD
Kenya	2	female	Hypo	OPD
Kenya	5	female	fever	OPD
Kenya	1	female	LCI	OPD
Kenya	5	female	fever	OPD
Kenya	1	Male	Hypo	OPD
Kenya	4	Male	LCI	OPD
Kenya	5	female	fever	OPD
Kenya	3	Male	>1 CSI	OPD
Kenya	6	female	fever	OPD
Kenya	1	Male	Hypo	OPD
Kenya	3	female	>1 CSI	OPD
Kenya	4	Male	>1 CSI	OPD
Kenya	2	Male	fever	OPD

Kenya	1	female	>1 CSI	OPD
Kenya	4	Male	fever	OPD
Kenya	4	female	Hypo	OPD
Kenya	3	Male	LCI	OPD
Kenya	1	female	>1 CSI	OPD
Kenya	4	Male	>1 CSI	OPD
Kenya	1	Male	Hypo	OPD
Kenya	6	Male	Hypo	OPD
Kenya	6	Male	LCI	OPD
Kenya	6	female	fever	OPD
Kenya	6	Male	fever	OPD
Kenya	1	female	Hypo	OPD
Kenya	6	female	>1 CSI	OPD
Kenya	3	Male	>1 CSI	OPD
Kenya	3	Male	fever	OPD
Kenya	3	female	Hypo	OPD
Kenya	6	Male	LCI	OPD
Kenya	2	Male	poor feeding	OPD
Kenya	4	Male	poor feeding	OPD
Kenya	4	female	fever	OPD
Kenya	4	Male	LCI	OPD
Kenya	1	female	Hypo	OPD
Kenya	4	Male	LCI	OPD
Kenya	4	Male	>1 CSI	OPD
Kenya	1	Male	Hypo	OPD
Kenya	1	Male	Hypo	OPD
Kenya	2	Male	>1 CSI	OPD
Kenya	2	Male	>1 CSI	OPD
Kenya	4	Male	LCI	OPD
Kenya	4	Male	>1 CSI	OPD
Kenya	4	female	fever	OPD
Kenya	5	female	LCI	OPD
Kenya	2	female	Hypo	OPD
Kenya	2	Male	LCI	OPD
Kenya	5	Male	>1 CSI	OPD
Kenya	3	Male	LCI	OPD
Kenya	3	Male	>1 CSI	OPD
Kenya	2	Male	>1 CSI	OPD
Kenya	6	female	LCI	OPD
Kenya	6	female	poor feeding	OPD
Kenya	6	Male	LCI	OPD
Kenya	6	Male	LCI	OPD
Kenya	3	Male	LCI	OPD
Kenya	4	Male	LCI	OPD
Kenya	2	female	fever	OPD
Kenya	2	female	>1 CSI	OPD
Kenya	6	Male	fever	OPD
Kenya	1	Male	fever	OPD
Kenya	3	Male	LCI	OPD
Kenya	4	female	LCI	OPD

Kenya	6	Male	poor feeding	OPD
Kenya	6	female	Hypo	OPD
Kenya	1	Male	>1 CSI	OPD
Kenya	3	female	Hypo	OPD
Kenya	11	Male	LCI	OPD
Kenya	19	female	Hypo	OPD
Kenya	19	female	Hypo	OPD
Kenya	21	Male	LCI	OPD
Kenya	11	Male	poor feeding	OPD
Kenya	16	female	fever	OPD
Kenya	7	Male	fever	OPD
Kenya	44	Male	LCI	OPD
Kenya	8	female	fever	OPD
Kenya	22	Male	LCI	OPD
Kenya	11	female	LCI	OPD
Kenya	38	female	LCI	OPD
Kenya	10	female	LCI	OPD
Kenya	28	Male	LCI	OPD
Kenya	12	Male	LCI	OPD
Kenya	28	female	fever	OPD
Kenya	9	Male	>1 CSI	OPD
Kenya	25	female	LCI	OPD
Kenya	7	Male	>1 CSI	OPD
Kenya	12	female	fever	OPD
Kenya	12	female	LCI	OPD
Kenya	10	female	LCI	OPD
Kenya	14	Male	LCI	OPD
Kenya	23	female	LCI	OPD
Kenya	8	Male	>1 CSI	OPD
Kenya	15	female	poor feeding	OPD
Kenya	20	Male	>1 CSI	OPD
Kenya	59	Male	fever	OPD
Kenya	6	female	>1 CSI	OPD
Kenya	13	Male	LCI	OPD
Kenya	17	Male	LCI	OPD
Kenya	21	Male	fever	OPD
Kenya	17	Male	fever	OPD
Kenya	7	Male	LCI	OPD
Kenya	14	female	LCI	OPD
Kenya	30	female	fever	OPD
Kenya	30	female	fever	OPD
Kenya	27	female	LCI	OPD
Kenya	57	Male	LCI	OPD
Kenya	21	female	LCI	OPD
Kenya	11	Male	LCI	OPD
Kenya	10	Male	fever	OPD
Kenya	12	female	LCI	OPD
Kenya	39	female	fever	OPD
Kenya	2	female	LCI	OPD
Kenya	11	Male	fever	OPD

Kenya	31	Male	LCI	OPD
Kenya	17	female	>1 CSI	OPD
Kenya	18	female	fever	OPD
Kenya	14	female	LCI	OPD
Kenya	23	Male	LCI	OPD
Kenya	14	female	fever	OPD
Kenya	19	female	fever	OPD
Kenya	10	Male	>1 CSI	OPD
Kenya	40	Male	LCI	OPD
Kenya	31	Male	LCI	OPD
Kenya	31	female	LCI	OPD
Kenya	18	Male	>1 CSI	OPD
Kenya	41	Male	fever	OPD
Kenya	8	female	LCI	OPD
Kenya	3	Male	fever	OPD
Kenya	16	Male	fever	OPD
Kenya	57	Male	LCI	OPD
Kenya	20	female	LCI	OPD
Kenya	7	female	poor feeding	OPD
Kenya	19	female	LCI	OPD
Kenya	34	Male	LCI	OPD
Kenya	9	Male	LCI	OPD
Kenya	26	Male	LCI	OPD
Kenya	17	female	LCI	OPD
Kenya	9	Male	fever	OPD
Kenya	47	Male	fever	OPD
Kenya	14	Male	>1 CSI	OPD
Kenya	12	Male	fever	OPD
Kenya	38	Male	LCI	OPD
Kenya	47	female	fever	OPD
Kenya	21	female	>1 CSI	OPD
Kenya	42	female	fever	OPD
Kenya	1	female	LCI	OPD
Kenya	46	Male	>1 CSI	OPD
Kenya	17	female	LCI	OPD
Kenya	29	female	LCI	OPD
Kenya	21	female	LCI	OPD
Kenya	12	female	>1 CSI	OPD
Kenya	31	female	fever	OPD
Kenya	10	female	poor feeding	OPD
Kenya	28	Male	LCI	OPD
Kenya	33	Male	>1 CSI	OPD
Kenya	20	female	LCI	OPD
Kenya	14	Male	LCI	OPD
Kenya	17	female	LCI	OPD
Kenya	8	female	Hypo	OPD
Kenya	8	female	fever	OPD
Kenya	13	female	fever	OPD
Kenya	28	Male	LCI	OPD
Kenya	40	female	>1 CSI	OPD

Kenya	17	female	>1 CSI	OPD
Kenya	18	female	fever	OPD
Kenya	22	Male	LCI	OPD
Kenya	20	Male	LCI	OPD
Kenya	13	female	LCI	OPD
Kenya	34	female	fever	OPD
Kenya	26	female	fever	OPD
Kenya	58	female	>1 CSI	OPD
Kenya	10	Male	fever	OPD
Kenya	13	female	fever	OPD
Kenya	11	Male	LCI	OPD
Kenya	17	Male	LCI	OPD
Kenya	14	Male	>1 CSI	OPD
Kenya	9	Male	fever	OPD
Kenya	21	female	poor feeding	OPD
Kenya	7	female	poor feeding	OPD
Kenya	8	Male	LCI	OPD
Kenya	8	female	poor feeding	OPD
Kenya	18	Male	LCI	OPD
Kenya	46	female	fever	OPD
Kenya	14	female	poor feeding	OPD
Kenya	46	Male	LCI	OPD
Kenya	31	Male	poor feeding	OPD
Kenya	18	Male	LCI	OPD
Kenya	13	female	>1 CSI	OPD
Kenya	28	Male	>1 CSI	OPD
Kenya	21	female	LCI	OPD
Kenya	21	female	LCI	OPD
Kenya	38	female	LCI	OPD
Kenya	13	Male	LCI	OPD
Kenya	42	female	fever	OPD
Kenya	12	Male	LCI	OPD
Kenya	8	female	fever	OPD
Kenya	20	Male	LCI	OPD
Kenya	11	female	fever	OPD
Kenya	51	Male	fever	OPD
Kenya	21	female	poor feeding	OPD
Kenya	14	Male	fever	OPD
Kenya	19	female	LCI	OPD
Kenya	11	female	fever	OPD
Kenya	55	female	fever	OPD
Kenya	4	female	>1 CSI	OPD
Kenya	24	female	LCI	OPD
Kenya	9	Male	poor feeding	OPD
Kenya	32	female	LCI	OPD
Kenya	22	female	Hypo	OPD
Kenya	20	female	LCI	OPD
Kenya	11	Male	>1 CSI	OPD
Kenya	16	female	LCI	OPD
Kenya	10	female	fever	OPD

Kenya	44	female	>1 CSI	OPD
Kenya	9	Male	poor feeding	OPD
Kenya	16	female	poor feeding	OPD
Kenya	10	Male	>1 CSI	OPD
Kenya	7	female	>1 CSI	OPD
Kenya	11	Male	fever	OPD
Kenya	19	female	LCI	OPD
Kenya	57	Male	LCI	OPD
Kenya	14	female	fever	OPD
Kenya	12	female	fever	OPD
Kenya	28	female	>1 CSI	OPD
Kenya	32	Male	LCI	OPD
Kenya	7	Male	LCI	OPD
Kenya	55	female	LCI	OPD
Kenya	32	Male	fever	OPD
Kenya	53	female	>1 CSI	OPD
Kenya	14	Male	LCI	OPD
Kenya	11	female	LCI	OPD
Kenya	25	Male	LCI	OPD
Kenya	7	female	>1 CSI	OPD
Kenya	27	female	poor feeding	OPD
Kenya	42	Male	>1 CSI	OPD
Kenya	7	female	LCI	OPD
Kenya	29	Male	LCI	OPD
Kenya	25	female	fever	OPD
Kenya	17	female	poor feeding	OPD
Kenya	16	female	>1 CSI	OPD
Kenya	8	Male	LCI	OPD
Kenya	6	Male	LCI	OPD
Kenya	22	Male	LCI	OPD
Kenya	13	female	fever	OPD
Kenya	32	female	LCI	OPD
Kenya	32	female	LCI	OPD
Kenya	51	female	LCI	OPD
Kenya	21	female	fever	OPD
Kenya	48	Male	LCI	OPD
Kenya	10	Male	poor feeding	OPD
Kenya	42	female	>1 CSI	OPD
Kenya	22	female	>1 CSI	OPD
Kenya	43	female	fever	OPD
Kenya	12	Male	fever	OPD
Kenya	16	Male	>1 CSI	OPD
Kenya	10	female	LCI	OPD
Kenya	40	Male	fever	OPD
Kenya	7	Male	>1 CSI	OPD
Kenya	35	female	fever	OPD
Kenya	7	Male	LCI	OPD
Kenya	9	female	LCI	OPD
Kenya	15	Male	fever	OPD
Kenya	9	Male	LCI	OPD

Kenya	44	female	fever	OPD
Kenya	19	female	LCI	OPD
Kenya	30	female	fever	OPD
Kenya	35	female	fever	OPD
Kenya	49	Male	poor feeding	OPD
Kenya	29	Male	fever	OPD
Kenya	16	female	>1 CSI	OPD
Kenya	10	female	fever	OPD
Kenya	16	Male	LCI	OPD
Kenya	14	female	poor feeding	OPD
Kenya	44	female	>1 CSI	OPD
Kenya	44	Male	LCI	OPD
Kenya	32	Male	LCI	OPD
Kenya	36	female	fever	OPD
Kenya	50	Male	LCI	OPD
Kenya	23	Male	LCI	OPD
Kenya	49	Male	LCI	OPD
Kenya	8	Male	LCI	OPD
Kenya	15	Male	fever	OPD
Kenya	8	female	>1 CSI	OPD
Kenya	41	Male	fever	OPD
Kenya	28	female	LCI	OPD
Kenya	13	Male	LCI	OPD
Kenya	10	Male	LCI	OPD
Kenya	29	female	LCI	OPD
Kenya	20	Male	LCI	OPD
Kenya	35	Male	LCI	OPD
Kenya	10	female	>1 CSI	OPD
Kenya	7	Male	poor feeding	OPD
Kenya	20	Male	fever	OPD
Kenya	14	female	>1 CSI	OPD
Kenya	12	Male	LCI	OPD
Kenya	27	female	fever	OPD
Kenya	13	Male	LCI	OPD
Kenya	40	female	fever	OPD
Kenya	14	Male	fever	OPD
Kenya	18	female	LCI	OPD
Kenya	39	female	fever	OPD
Kenya	3	female	LCI	OPD
Kenya	17	female	LCI	OPD
Kenya	12	Male	>1 CSI	OPD
Kenya	48	Male	LCI	OPD
Kenya	46	female	LCI	OPD
Kenya	43	Male	LCI	OPD
Kenya	27	female	>1 CSI	OPD
Kenya	57	female	fever	OPD
Kenya	30	Male	>1 CSI	OPD
Kenya	19	Male	LCI	OPD
Kenya	28	female	LCI	OPD
Kenya	10	female	LCI	OPD

Kenya	30	female	LCI	OPD
Kenya	27	Male	fever	OPD
Kenya	40	Male	LCI	OPD
Kenya	22	Male	>1 CSI	OPD
Kenya	44	Male	poor feeding	OPD
Kenya	58	Male	movement on stimula	OPD
Kenya	13	Male	>1 CSI	OPD
Kenya	29	Male	LCI	OPD
Kenya	25	Male	LCI	OPD
Kenya	25	Male	fever	OPD
Kenya	54	Male	fever	OPD
Kenya	33	Male	fever	OPD
Kenya	45	Male	LCI	OPD
Kenya	37	female	>1 CSI	OPD
Kenya	16	Male	fever	OPD
Kenya	39	female	LCI	OPD
Kenya	13	Male	fever	OPD
Kenya	45	female	fever	OPD
Kenya	9	female	movement on stimula	OPD
Kenya	35	Male	LCI	OPD
Kenya	25	female	>1 CSI	OPD
Kenya	56	female	fever	OPD
Kenya	14	Male	>1 CSI	OPD
Kenya	27	female	fever	OPD
Kenya	47	female	fever	OPD
Kenya	10	female	fever	OPD
Kenya	35	female	fever	OPD
Kenya	42	Male	fever	OPD
Kenya	37	female	fever	OPD
Kenya	16	female	LCI	OPD
Kenya	39	female	fever	OPD
Kenya	18	female	>1 CSI	OPD
Kenya	36	Male	LCI	OPD
Kenya	8	Male	LCI	OPD
Kenya	10	Male	fever	OPD
Kenya	18	Male	LCI	OPD
Kenya	13	Male	fever	OPD
Kenya	48	female	fever	OPD
Kenya	26	Male	>1 CSI	OPD
Kenya	54	female	>1 CSI	OPD
Kenya	8	female	fever	OPD
Kenya	59	female	>1 CSI	OPD
Kenya	29	Male	LCI	OPD
Kenya	46	Male	LCI	OPD
Kenya	42	Male	LCI	OPD
Kenya	43	Male	LCI	OPD
Kenya	24	female	LCI	OPD
Kenya	56	female	LCI	OPD
Kenya	27	Male	LCI	OPD
Kenya	35	female	>1 CSI	OPD

Kenya	13	female	poor feeding	OPD
Kenya	57	Male	LCI	OPD
Kenya	22	Male	LCI	OPD
Kenya	22	female	LCI	OPD
Kenya	28	female	LCI	OPD
Kenya	28	female	LCI	OPD
Kenya	49	Male	>1 CSI	OPD
Kenya	8	female	fever	OPD
Kenya	10	Male	fever	OPD
Kenya	21	female	>1 CSI	OPD
Kenya	45	Male	>1 CSI	OPD
Kenya	31	Male	poor feeding	OPD
Kenya	33	Male	poor feeding	OPD
Kenya	39	Male	poor feeding	OPD
Kenya	19	Male	LCI	OPD
Kenya	7	Male	LCI	OPD
Kenya	57	Male	fever	OPD
Kenya	17	female	>1 CSI	OPD
Kenya	52	Male	LCI	OPD
Kenya	24	female	>1 CSI	OPD
Kenya	27	Male	fever	OPD
Kenya	14	female	fever	OPD
Kenya	51	Male	fever	OPD
Kenya	40	Male	fever	OPD
Kenya	53	Male	>1 CSI	OPD
Kenya	33	female	>1 CSI	OPD
Kenya	23	female	poor feeding	OPD
Kenya	29	Male	LCI	OPD
Kenya	22	female	fever	OPD
Kenya	59	Male	>1 CSI	OPD
Kenya	21	female	LCI	OPD
Kenya	27	Male	LCI	OPD
Kenya	20	female	LCI	OPD
Kenya	28	female	LCI	OPD
Kenya	21	female	LCI	OPD
Kenya	16	Male	LCI	OPD
Kenya	33	female	fever	OPD
Kenya	49	female	>1 CSI	OPD
Kenya	57	Male	poor feeding	OPD
Kenya	43	female	>1 CSI	OPD
Kenya	49	female	fever	OPD
Kenya	11	Male	LCI	OPD
Kenya	29	female	>1 CSI	OPD
Kenya	44	female	LCI	OPD
Kenya	1	Male	movement on stimula	OPD
Kenya	12	Male	LCI	OPD
Kenya	56	female	fever	OPD
Kenya	32	Male	LCI	OPD
Kenya	23	female	>1 CSI	OPD
Kenya	12	Male	Hypo	OPD

Kenya	14	Male	LCI	OPD
Kenya	19	female	LCI	OPD
Kenya	19	female	>1 CSI	OPD
Kenya	42	female	LCI	OPD
Kenya	59	Male	poor feeding	OPD
Kenya	10	Male	LCI	OPD
Kenya	36	Male	LCI	OPD
Kenya	43	Male	>1 CSI	OPD
Kenya	39	female	LCI	OPD
Kenya	14	female	LCI	OPD
Kenya	51	Male	LCI	OPD
Kenya	21	female	>1 CSI	OPD
Kenya	17	female	LCI	OPD
Kenya	47	female	fever	OPD
Kenya	13	female	fever	OPD
Kenya	16	Male	LCI	OPD
Kenya	30	female	LCI	OPD
Kenya	17	Male	LCI	OPD
Kenya	10	Male	fever	OPD
Kenya	3	Male	>1 CSI	OPD
Kenya	18	female	LCI	OPD
Kenya	26	Male	LCI	OPD
Kenya	9	Male	>1 CSI	OPD
Kenya	32	Male	>1 CSI	OPD
Kenya	19	Male	LCI	OPD
Kenya	16	female	>1 CSI	OPD
Kenya	18	female	LCI	OPD
Kenya	42	Male	LCI	OPD
Kenya	58	female	fever	OPD
Kenya	37	Male	fever	OPD
Kenya	34	Male	LCI	OPD
Kenya	59	female	>1 CSI	OPD
Kenya	21	Male	>1 CSI	OPD
Kenya	22	Male	LCI	OPD
Kenya	33	female	fever	OPD
Kenya	42	female	LCI	OPD
Kenya	40	female	LCI	OPD
Kenya	2	Male	LCI	OPD
Kenya	56	female	LCI	OPD
Kenya	29	Male	LCI	OPD
Kenya	28	female	fever	OPD
Kenya	37	female	poor feeding	OPD
Kenya	29	female	LCI	OPD
Kenya	17	Male	LCI	OPD
Kenya	34	female	>1 CSI	OPD
Kenya	56	Male	LCI	OPD
Kenya	7	Male	>1 CSI	OPD
Kenya	35	Male	LCI	OPD
Kenya	8	female	>1 CSI	OPD
Kenya	27	female	poor feeding	OPD

Kenya	49	female	>1 CSI	OPD
Kenya	14	Male	LCI	OPD
Kenya	41	female	LCI	OPD
Kenya	29	female	>1 CSI	OPD
Kenya	17	Male	LCI	OPD
Kenya	44	female	>1 CSI	OPD
Kenya	23	Male	fever	OPD
Kenya	14	female	LCI	OPD
Kenya	10	female	LCI	OPD
Kenya	21	Male	LCI	OPD
Kenya	21	Male	LCI	OPD
Kenya	15	Male	LCI	OPD
Kenya	22	Male	fever	OPD
Kenya	53	female	fever	OPD
Kenya	30	Male	LCI	OPD
Kenya	12	Male	LCI	OPD
Kenya	15	Male	>1 CSI	OPD
Kenya	21	Male	LCI	OPD
Kenya	54	Male	fever	OPD
Kenya	35	Male	LCI	OPD
Kenya	24	female	LCI	OPD
Kenya	8	Male	Hypo	OPD
Kenya	17	female	LCI	OPD
Kenya	28	female	LCI	OPD
Kenya	33	Male	>1 CSI	OPD
Kenya	24	Male	LCI	OPD
Kenya	12	female	LCI	OPD
Kenya	51	Male	LCI	OPD
Kenya	16	female	LCI	OPD
Kenya	53	Male	LCI	OPD
Kenya	8	Male	LCI	OPD
Kenya	21	female	fever	OPD
Kenya	52	female	fever	OPD
Kenya	14	female	fever	OPD
Kenya	11	female	>1 CSI	OPD
Kenya	10	Male	LCI	OPD
Kenya	9	female	>1 CSI	OPD
Kenya	20	female	>1 CSI	OPD
Kenya	8	Male	>1 CSI	OPD
Kenya	12	female	>1 CSI	OPD
Kenya	13	Male	>1 CSI	OPD
Kenya	27	female	poor feeding	OPD
Kenya	31	Male	>1 CSI	OPD
Kenya	15	female	LCI	OPD
Kenya	11	Male	poor feeding	OPD
Kenya	57	female	fever	OPD
Kenya	11	Male	poor feeding	OPD
Kenya	10	female	>1 CSI	OPD
Kenya	22	Male	LCI	OPD
Kenya	58	Male	poor feeding	OPD

Kenya	32	female	>1 CSI	OPD
Kenya	40	Male	LCI	OPD
Kenya	6	female	>1 CSI	OPD
Kenya	17	female	LCI	OPD
Kenya	17	female	>1 CSI	OPD
Kenya	11	female	LCI	OPD
Kenya	10	female	>1 CSI	OPD
Kenya	27	Male	LCI	OPD
Kenya	29	female	LCI	OPD
Kenya	16	female	LCI	OPD
Kenya	45	Male	LCI	OPD
Kenya	20	female	LCI	OPD
Kenya	14	female	poor feeding	OPD
Kenya	34	Male	LCI	OPD
Kenya	6	Male	>1 CSI	OPD
Kenya	16	Male	>1 CSI	OPD
Kenya	14	female	>1 CSI	OPD
Kenya	10	female	>1 CSI	OPD
Kenya	38	female	poor feeding	OPD
Kenya	56	Male	LCI	OPD
Kenya	18	Male	LCI	OPD
Kenya	39	female	LCI	OPD
Kenya	11	female	LCI	OPD
Kenya	14	female	>1 CSI	OPD
Kenya	15	Male	fever	OPD
Kenya	24	female	LCI	OPD
Kenya	58	female	poor feeding	OPD
Kenya	32	Male	LCI	OPD
Kenya	11	Male	LCI	OPD
Kenya	19	Male	LCI	OPD
Kenya	13	Male	LCI	OPD
Kenya	11	Male	>1 CSI	OPD
Kenya	55	female	LCI	OPD
Kenya	8	Male	poor feeding	OPD
Kenya	9	Male	LCI	OPD
Kenya	35	Male	fever	OPD
Kenya	16	female	fever	OPD
Kenya	46	female	LCI	OPD
Kenya	44	female	LCI	OPD
Kenya	49	female	fever	OPD
Kenya	25	female	LCI	OPD
Kenya	47	female	>1 CSI	OPD
Kenya	16	female	LCI	OPD
Kenya	20	female	LCI	OPD
Kenya	19	female	fever	OPD
Kenya	11	female	poor feeding	OPD
Kenya	16	Male	>1 CSI	OPD
Kenya	14	Male	>1 CSI	OPD
Kenya	12	female	LCI	OPD
Kenya	9	Male	LCI	OPD

Kenya	11	female	poor feeding	OPD
Kenya	13	female	LCI	OPD
Kenya	14	female	LCI	OPD
Kenya	23	female	>1 CSI	OPD
Kenya	19	female	poor feeding	OPD
Kenya	4	Male	LCI	OPD
Kenya	8	Male	Hypo	OPD
Kenya	13	female	>1 CSI	OPD
Kenya	50	Male	fever	OPD
Kenya	22	female	fever	OPD
Kenya	28	female	LCI	OPD
Kenya	35	female	fever	OPD
Kenya	7	female	LCI	OPD
Kenya	5	Male	LCI	OPD
Kenya	41	Male	poor feeding	OPD
Kenya	7	Male	Hypo	OPD
Kenya	47	Male	fever	OPD
Kenya	15	female	fever	OPD
Kenya	7	Male	LCI	OPD
Kenya	28	Male	LCI	OPD
Kenya	31	Male	LCI	OPD
Kenya	5	female	>1 CSI	OPD
Kenya	7	female	LCI	OPD
Kenya	4	female	LCI	OPD
Kenya	35	Male	>1 CSI	OPD
Kenya	27	Male	LCI	OPD
Kenya	19	female	LCI	OPD
Kenya	27	female	fever	OPD
Kenya	9	Male	>1 CSI	OPD
Kenya	22	female	LCI	OPD
Kenya	13	Male	fever	OPD
Kenya	41	female	LCI	OPD
Kenya	12	Male	fever	OPD
Kenya	30	female	LCI	OPD
Kenya	42	female	>1 CSI	OPD
Kenya	18	Male	LCI	OPD
Kenya	7	Male	fever	OPD
Kenya	46	female	fever	OPD
Kenya	34	Male	poor feeding	OPD
Kenya	19	Male	LCI	OPD
Kenya	15	female	LCI	OPD
Kenya	12	Male	fever	OPD
Kenya	18	female	LCI	OPD
Kenya	22	Male	LCI	OPD
Kenya	9	female	LCI	OPD
Kenya	4	female	fever	OPD
Kenya	41	female	LCI	OPD
Kenya	12	female	LCI	OPD
Kenya	7	Male	LCI	OPD
Kenya	44	Male	LCI	OPD

Kenya	20	female	>1 CSI	OPD
Kenya	8	Male	Hypo	OPD
Kenya	14	female	LCI	OPD
Kenya	10	Male	poor feeding	OPD
Kenya	14	Male	LCI	OPD
Kenya	6	Male	fever	OPD
Kenya	10	Male	poor feeding	OPD
Kenya	14	Male	poor feeding	OPD
Kenya	9	female	LCI	OPD
Kenya	5	Male	>1 CSI	OPD
Kenya	8	female	LCI	OPD
Kenya	37	Male	LCI	OPD
Kenya	11	female	movement on stimula	OPD
Kenya	37	female	fever	OPD
Kenya	4	Male	LCI	OPD
Kenya	8	female	LCI	OPD
Kenya	23	female	LCI	OPD
Kenya	19	Male	>1 CSI	OPD
Kenya	30	female	>1 CSI	OPD
Kenya	8	Male	>1 CSI	OPD
Kenya	10	female	>1 CSI	OPD
Kenya	6	Male	LCI	OPD
Kenya	21	Male	poor feeding	OPD
Kenya	14	female	>1 CSI	OPD
Kenya	59	female	poor feeding	OPD
Kenya	14	Male	fever	OPD
Kenya	41	Male	movement on stimula	OPD
Kenya	9	female	poor feeding	OPD
Kenya	37	Male	LCI	OPD
Kenya	58	Male	fever	OPD
Kenya	18	female	poor feeding	OPD
Kenya	45	female	LCI	OPD
Kenya	43	Male	LCI	OPD
Kenya	40	Male	LCI	OPD
Kenya	10	Male	LCI	OPD
Kenya	9	female	LCI	OPD
Kenya	59	Male	LCI	OPD
Kenya	13	Male	>1 CSI	OPD
Kenya	14	female	LCI	OPD
Kenya	9	Male	fever	OPD
Kenya	54	female	>1 CSI	OPD
Kenya	43	female	poor feeding	OPD
Kenya	8	Male	LCI	OPD
Kenya	7	Male	>1 CSI	OPD
Kenya	6	Male	fever	OPD
Kenya	4	female	>1 CSI	OPD
Kenya	38	female	>1 CSI	OPD
Kenya	51	female	LCI	OPD
Kenya	20	female	LCI	OPD
Kenya	27	female	fever	OPD

Kenya	22	Male	>1 CSI	OPD
Kenya	43	Male	LCI	OPD
Kenya	16	Male	LCI	OPD
Kenya	3	female	fever	OPD
Kenya	14	Male	LCI	OPD
Kenya	7	Male	fever	OPD
Kenya	51	Male	LCI	OPD
Kenya	22	Male	fever	OPD
Kenya	14	female	>1 CSI	OPD
Kenya	31	female	LCI	OPD
Kenya	12	female	fever	OPD
Kenya	13	female	fever	OPD
Kenya	4	Male	fever	OPD
Kenya	9	Male	LCI	OPD
Kenya	40	Male	LCI	OPD
Kenya	8	Male	Hypo	OPD
Kenya	7	Male	poor feeding	OPD
Kenya	11	Male	>1 CSI	OPD
Kenya	34	female	LCI	OPD
Kenya	13	female	>1 CSI	OPD
Kenya	31	Male	LCI	OPD
Kenya	15	Male	Hypo	OPD
Kenya	24	Male	LCI	OPD
Kenya	17	Male	LCI	OPD
Kenya	25	female	fever	OPD
Kenya	32	Male	fever	OPD
Kenya	11	Male	poor feeding	OPD
Kenya	12	Male	>1 CSI	OPD
Kenya	9	female	fever	OPD
Kenya	27	female	>1 CSI	OPD
Kenya	17	female	LCI	OPD
Kenya	12	female	fever	OPD
Kenya	59	female	fever	OPD
Kenya	28	female	poor feeding	OPD
Kenya	13	Male	fever	OPD
Kenya	40	Male	LCI	OPD
Kenya	25	female	LCI	OPD
Kenya	11	female	LCI	OPD
Kenya	28	female	LCI	OPD
Kenya	10	Male	LCI	OPD
Kenya	15	Male	LCI	OPD
Kenya	18	female	>1 CSI	OPD
Kenya	8	female	>1 CSI	OPD
Kenya	5	female	LCI	OPD
Kenya	33	female	poor feeding	OPD
Kenya	33	female	LCI	OPD
Kenya	24	female	>1 CSI	OPD
Kenya	18	Male	LCI	OPD
Kenya	18	female	LCI	OPD
Kenya	23	Male	movement on stimula	OPD

Kenya	5	female	LCI	OPD
Kenya	22	Male	LCI	OPD
Kenya	21	Male	>1 CSI	OPD
Kenya	32	Male	poor feeding	OPD
Kenya	21	Male	poor feeding	OPD
Kenya	23	Male	LCI	OPD
Kenya	9	Male	LCI	OPD
Kenya	19	female	>1 CSI	OPD
Kenya	34	female	poor feeding	OPD
Kenya	12	Male	LCI	OPD
Kenya	27	female	>1 CSI	OPD
Kenya	17	Male	LCI	OPD
Kenya	31	Male	LCI	OPD
Kenya	38	female	LCI	OPD
Kenya	22	Male	fever	OPD
Kenya	7	Male	>1 CSI	OPD
Kenya	14	female	fever	OPD
Kenya	9	Male	LCI	OPD
Kenya	10	Male	fever	OPD
Kenya	12	Male	LCI	OPD
Kenya	2	female	LCI	OPD
Kenya	15	Male	fever	OPD
Kenya	7	female	fever	OPD
Kenya	30	Male	fever	OPD
Kenya	5	female	LCI	OPD
Kenya	37	female	LCI	OPD
Kenya	37	Male	>1 CSI	OPD
Kenya	41	female	LCI	OPD
Kenya	15	female	>1 CSI	OPD
Kenya	7	female	fever	OPD
Kenya	9	Male	fever	OPD
Kenya	10	female	fever	OPD
Kenya	44	female	LCI	OPD
Kenya	12	female	fever	OPD
Kenya	17	Male	LCI	OPD
Kenya	3	female	LCI	OPD
Kenya	16	Male	>1 CSI	OPD
Kenya	16	female	fever	OPD
Kenya	14	Male	LCI	OPD
Kenya	13	Male	LCI	OPD
Kenya	12	Male	LCI	OPD
Kenya	28	Male	LCI	OPD
Kenya	24	female	LCI	OPD
Kenya	14	female	poor feeding	OPD
Kenya	44	female	LCI	OPD
Kenya	19	Male	LCI	OPD
Kenya	23	Male	poor feeding	OPD
Kenya	24	Male	LCI	OPD
Kenya	53	Male	LCI	OPD
Kenya	19	Male	LCI	OPD

Kenya	15	female	LCI	OPD
Kenya	44	Male	LCI	OPD
Kenya	32	female	LCI	OPD
Kenya	49	female	>1 CSI	OPD
Kenya	44	Male	LCI	OPD
Kenya	32	Male	LCI	OPD
Kenya	26	female	fever	OPD
Kenya	15	Male	fever	OPD
Kenya	19	Male	LCI	OPD
Kenya	7	Male	poor feeding	OPD
Kenya	15	Male	fever	OPD
Kenya	24	female	fever	OPD
Kenya	20	female	>1 CSI	OPD
Kenya	32	female	LCI	OPD
Kenya	5	Male	LCI	OPD
Kenya	26	female	fever	OPD
Kenya	40	Male	LCI	OPD
Kenya	23	Male	>1 CSI	OPD
Kenya	39	Male	>1 CSI	OPD
Kenya	33	Male	LCI	OPD
Kenya	22	Male	LCI	OPD
Kenya	12	female	>1 CSI	OPD
Kenya	25	Male	Hypo	OPD
Kenya	42	female	>1 CSI	OPD
Kenya	12	Male	LCI	OPD
Kenya	37	Male	LCI	OPD
Kenya	20	female	fever	OPD
Kenya	13	Male	LCI	OPD
Kenya	41	Male	>1 CSI	OPD
Kenya	12	female	LCI	OPD
Kenya	11	Male	LCI	OPD
Kenya	10	Male	LCI	OPD
Kenya	17	Male	LCI	OPD
Kenya	8	Male	LCI	OPD
Kenya	13	female	LCI	OPD
Kenya	41	female	fever	OPD
Kenya	20	Male	poor feeding	OPD
Kenya	43	female	LCI	OPD
Kenya	2	Male	fever	OPD
Kenya	44	female	fever	OPD
Kenya	22	female	fever	OPD
Kenya	10	Male	>1 CSI	OPD
Kenya	40	female	fever	OPD
Kenya	2	Male	LCI	OPD
Kenya	45	female	LCI	OPD
Kenya	21	Male	LCI	OPD
Kenya	12	female	LCI	OPD
Kenya	9	female	>1 CSI	OPD
Kenya	11	Male	poor feeding	OPD
Kenya	10	Male	LCI	OPD

Kenya	1	Male	LCI	OPD
Kenya	44	Male	LCI	OPD
Kenya	32	female	LCI	OPD
Kenya	13	Male	fever	OPD
Kenya	8	Male	fever	OPD
Kenya	26	Male	LCI	OPD
Kenya	6	Male	fever	OPD
Kenya	13	female	>1 CSI	OPD
Kenya	7	Male	LCI	OPD
Kenya	8	Male	>1 CSI	OPD
Kenya	15	female	>1 CSI	OPD
Kenya	14	Male	fever	OPD
Kenya	2	female	LCI	OPD
Kenya	5	female	LCI	OPD
Kenya	15	Male	fever	OPD
Kenya	16	female	>1 CSI	OPD
Kenya	38	female	LCI	OPD
Kenya	16	Male	fever	OPD
Kenya	22	Male	LCI	OPD
Kenya	12	Male	LCI	OPD
Kenya	36	Male	fever	OPD
Kenya	43	female	fever	OPD
Kenya	22	female	fever	OPD
Kenya	7	Male	>1 CSI	OPD
Kenya	22	Male	fever	OPD
Kenya	43	female	LCI	OPD
Kenya	18	Male	LCI	OPD
Kenya	18	female	LCI	OPD
Kenya	4	female	LCI	OPD
Kenya	47	female	fever	OPD
Kenya	31	Male	LCI	OPD
Kenya	47	female	fever	OPD
Kenya	10	Male	>1 CSI	OPD
Kenya	28	female	LCI	OPD
Kenya	14	Male	LCI	OPD
Kenya	32	female	LCI	OPD
Kenya	23	female	fever	OPD
Kenya	19	Male	>1 CSI	OPD
Kenya	38	Male	LCI	OPD
Kenya	28	female	fever	OPD
Kenya	16	Male	LCI	OPD
Kenya	14	female	>1 CSI	OPD
Kenya	7	Male	LCI	OPD
Kenya	34	female	LCI	OPD
Kenya	35	female	LCI	OPD
Kenya	34	Male	fever	OPD
Kenya	18	Male	LCI	OPD
Kenya	9	Male	Hypo	OPD
Kenya	36	female	LCI	OPD
Kenya	15	female	fever	OPD

Kenya	8	female	LCI	OPD
Kenya	31	female	>1 CSI	OPD
Kenya	46	Male	fever	OPD
Kenya	25	female	>1 CSI	OPD
Kenya	31	female	LCI	OPD
Kenya	27	Male	LCI	OPD
Kenya	12	Male	>1 CSI	OPD
Kenya	31	female	LCI	OPD
Kenya	18	Male	LCI	OPD
Kenya	25	Male	LCI	OPD
Kenya	22	Male	LCI	OPD
Kenya	9	Male	LCI	OPD
Kenya	14	Male	>1 CSI	OPD
Kenya	9	female	fever	OPD
Kenya	48	Male	LCI	OPD
Kenya	52	female	fever	OPD
Kenya	5	Male	fever	OPD
Kenya	5	Male	>1 CSI	OPD
Kenya	20	female	LCI	OPD
Kenya	20	Male	LCI	OPD
Kenya	14	female	fever	OPD
Kenya	27	female	fever	OPD
Kenya	9	female	fever	OPD
Kenya	27	Male	LCI	OPD
Kenya	16	female	LCI	OPD
Kenya	22	female	LCI	OPD
Kenya	8	female	LCI	OPD
Kenya	18	Male	fever	OPD
Kenya	56	Male	LCI	OPD
Kenya	5	female	fever	OPD
Kenya	11	female	LCI	OPD
Kenya	45	female	>1 CSI	OPD
Kenya	9	Male	LCI	OPD
Kenya	8	Male	>1 CSI	OPD
Kenya	14	female	fever	OPD
Kenya	11	female	fever	OPD
Kenya	15	female	LCI	OPD
Kenya	26	female	LCI	OPD
Kenya	15	female	fever	OPD
Kenya	6	female	LCI	OPD
Kenya	17	female	fever	OPD
Kenya	15	Male	fever	OPD
Kenya	12	female	fever	OPD
Kenya	11	Male	>1 CSI	OPD
Kenya	2	Male	LCI	OPD
Kenya	2	female	LCI	OPD
Kenya	7	Male	LCI	OPD
Kenya	43	female	fever	OPD
Kenya	11	Male	>1 CSI	OPD
Kenya	15	female	LCI	OPD

Kenya	13	Male	fever	OPD
Kenya	20	Male	LCI	OPD
Kenya	12	female	fever	OPD
Kenya	26	Male	LCI	OPD
Kenya	11	Male	LCI	OPD
Kenya	15	Male	LCI	OPD
Kenya	12	female	>1 CSI	OPD
Kenya	13	Male	LCI	OPD
Kenya	10	female	poor feeding	OPD
Kenya	9	Male	LCI	OPD
Kenya	45	Male	LCI	OPD
Kenya	9	Male	LCI	OPD
Kenya	31	Male	fever	OPD
Kenya	27	female	>1 CSI	OPD
Kenya	54	female	fever	OPD
Kenya	37	Male	LCI	OPD
Kenya	33	Male	fever	OPD
Kenya	47	female	fever	OPD
Kenya	7	Male	>1 CSI	OPD
Kenya	22	Male	LCI	OPD
Kenya	27	female	fever	OPD
Kenya	13	Male	LCI	OPD
Kenya	24	Male	LCI	OPD
Kenya	29	Male	>1 CSI	OPD
Kenya	16	Male	LCI	OPD
Kenya	11	female	fever	OPD
Kenya	39	Male	LCI	OPD
Kenya	48	female	LCI	OPD
Kenya	5	Male	LCI	OPD
Kenya	14	Male	LCI	OPD
Kenya	9	female	LCI	OPD
Kenya	23	female	LCI	OPD
Kenya	9	Male	poor feeding	OPD
Kenya	9	Male	LCI	OPD
Kenya	54	Male	fever	OPD
Kenya	25	Male	>1 CSI	OPD
Kenya	36	Male	LCI	OPD
Kenya	20	Male	LCI	OPD
Kenya	4	Male	LCI	OPD
Kenya	25	Male	fever	OPD
Kenya	18	Male	fever	OPD
Kenya	21	Male	LCI	OPD
Kenya	28	Male	fever	OPD
Kenya	23	Male	LCI	OPD
Kenya	43	Male	LCI	OPD
Kenya	12	female	>1 CSI	OPD
Kenya	19	female	LCI	OPD
Kenya	42	female	>1 CSI	OPD
Kenya	16	Male	>1 CSI	OPD
Kenya	12	female	fever	OPD

Kenya	31	Male	LCI	OPD
Kenya	54	Male	LCI	OPD
Kenya	14	Male	>1 CSI	OPD
Kenya	18	Male	LCI	OPD
Kenya	11	female	poor feeding	OPD
Kenya	40	Male	LCI	OPD
Kenya	22	Male	LCI	OPD
Kenya	14	Male	fever	OPD
Kenya	22	female	>1 CSI	OPD
Kenya	10	female	fever	OPD
Kenya	10	female	>1 CSI	OPD
Kenya	18	female	fever	OPD
Kenya	13	female	>1 CSI	OPD
Kenya	26	female	LCI	OPD
Kenya	22	Male	LCI	OPD
Kenya	16	female	fever	OPD
Kenya	8	female	LCI	OPD
Kenya	18	female	fever	OPD
Kenya	35	female	poor feeding	OPD
Kenya	8	female	>1 CSI	OPD
Kenya	19	Male	>1 CSI	OPD
Kenya	28	Male	LCI	OPD
Kenya	21	Male	fever	OPD
Kenya	42	Male	>1 CSI	OPD
Kenya	27	Male	LCI	OPD
Kenya	18	Male	fever	OPD
Kenya	7	female	>1 CSI	OPD
Kenya	8	female	>1 CSI	OPD
Kenya	25	Male	fever	OPD
Kenya	42	female	LCI	OPD
Kenya	11	Male	>1 CSI	OPD
Kenya	17	Male	LCI	OPD
Kenya	4	Male	>1 CSI	OPD
Kenya	44	female	fever	OPD
Kenya	12	female	LCI	OPD
Kenya	30	female	LCI	OPD
Kenya	19	Male	LCI	OPD
Kenya	6	Male	>1 CSI	OPD
Kenya	18	Male	LCI	OPD
Kenya	40	Male	>1 CSI	OPD
Kenya	15	female	LCI	OPD
Kenya	45	female	fever	OPD
Kenya	10	Male	>1 CSI	OPD
Kenya	21	female	>1 CSI	OPD
Kenya	18	female	LCI	OPD
Kenya	40	Male	LCI	OPD
Kenya	11	female	fever	OPD
Kenya	22	female	>1 CSI	OPD
Kenya	41	female	fever	OPD
Kenya	29	female	LCI	OPD

Kenya	17	female	fever	OPD
Kenya	51	Male	LCI	OPD
Kenya	14	Male	LCI	OPD
Kenya	19	Male	fever	OPD
Kenya	11	Male	LCI	OPD
Kenya	8	female	>1 CSI	OPD
Kenya	21	Male	LCI	OPD
Kenya	13	female	LCI	OPD
Kenya	33	female	LCI	OPD
Kenya	35	Male	LCI	OPD
Kenya	31	Male	fever	OPD
Kenya	27	Male	LCI	OPD
Kenya	10	Male	LCI	OPD
Kenya	19	female	LCI	OPD
Kenya	22	Male	LCI	OPD
Kenya	19	female	fever	OPD
Kenya	12	Male	LCI	OPD
Kenya	12	Male	fever	OPD
Kenya	27	female	fever	OPD
Kenya	24	female	fever	OPD
Kenya	29	Male	LCI	OPD
Kenya	12	Male	>1 CSI	OPD
Kenya	37	Male	fever	OPD
Kenya	14	female	fever	OPD
Kenya	13	female	>1 CSI	OPD
Kenya	36	Male	fever	OPD
Kenya	30	Male	fever	OPD
Kenya	6	female	>1 CSI	OPD
Kenya	40	Male	LCI	OPD
Kenya	53	Male	fever	OPD
Kenya	12	Male	>1 CSI	OPD
Kenya	20	Male	>1 CSI	OPD
Kenya	20	female	>1 CSI	OPD
Kenya	19	Male	LCI	OPD
Kenya	24	Male	fever	OPD
Kenya	18	Male	fever	OPD
Kenya	48	Male	LCI	OPD
Kenya	14	female	LCI	OPD
Kenya	11	Male	fever	OPD
Kenya	12	Male	fever	OPD
Kenya	14	Male	LCI	OPD
Kenya	8	female	LCI	OPD
Kenya	21	female	fever	OPD
Kenya	8	female	LCI	OPD
Kenya	30	female	fever	OPD
Kenya	45	Male	>1 CSI	OPD
Kenya	12	female	>1 CSI	OPD
Kenya	17	female	fever	OPD
Kenya	14	Male	LCI	OPD
Kenya	48	female	>1 CSI	OPD

Kenya	15	Male	>1 CSI	OPD
Kenya	15	Male	>1 CSI	OPD
Kenya	8	Male	>1 CSI	OPD
Kenya	13	Male	LCI	OPD
Kenya	14	Male	>1 CSI	OPD
Kenya	10	female	>1 CSI	OPD
Kenya	11	Male	LCI	OPD
Kenya	48	Male	>1 CSI	OPD
Kenya	8	female	LCI	OPD
Kenya	23	Male	fever	OPD
Kenya	40	Male	LCI	OPD
Kenya	12	Male	LCI	OPD
Kenya	49	Male	LCI	OPD
Kenya	43	female	LCI	OPD
Kenya	15	Male	fever	OPD
Kenya	27	Male	LCI	OPD
Kenya	3	Male	>1 CSI	OPD
Kenya	29	Male	LCI	OPD
Kenya	10	Male	LCI	OPD
Kenya	25	Male	LCI	OPD
Kenya	40	Male	LCI	OPD
Kenya	11	female	>1 CSI	OPD
Kenya	28	female	LCI	OPD
Kenya	45	Male	LCI	OPD
Kenya	10	female	poor feeding	OPD
Kenya	12	Male	>1 CSI	OPD
Kenya	58	Male	LCI	OPD
Kenya	36	Male	LCI	OPD
Kenya	11	Male	LCI	OPD
Kenya	25	Male	>1 CSI	OPD
Kenya	38	Male	LCI	OPD
Kenya	26	female	LCI	OPD
Kenya	13	female	Hypo	OPD
Kenya	12	Male	>1 CSI	OPD
Kenya	24	Male	>1 CSI	OPD
Kenya	1	Male	fever	OPD
Kenya	23	Male	LCI	OPD
Kenya	41	Male	LCI	OPD
Kenya	17	Male	fever	OPD
Kenya	22	Male	fever	OPD
Kenya	36	Male	LCI	OPD
Kenya	14	female	LCI	OPD
Kenya	15	female	LCI	OPD
Kenya	1	female	fb0-6	OPD
Kenya	5	female	fb0-6	OPD
Kenya	3	female	fb0-6	OPD
Kenya	4	female	fb0-6	OPD
Kenya	3	Male	fb0-6	OPD
Kenya	3	female	fb0-6	OPD
Kenya	3	Male	fb0-6	OPD

Kenya	4	Male	fb0-6	OPD
Kenya	6	female	fb0-6	OPD
Kenya	6	Male	fb0-6	OPD
Kenya	6	female	fb0-6	OPD
Kenya	6	Male	fb0-6	OPD
Kenya	4	female	fb0-6	OPD
Kenya	5	female	fb0-6	OPD
Kenya	3	Male	fb0-6	OPD
Kenya	3	female	fb0-6	OPD
Kenya	4	female	fb0-6	OPD
Kenya	3	Male	fb0-6	OPD
Kenya	1	female	fb0-6	OPD
Kenya	1	Male	fb0-6	OPD
Kenya	2	female	fb0-6	OPD
Kenya	4	Male	fb0-6	OPD
Kenya	4	Male	fb0-6	OPD
Kenya	2	Male	fb0-6	OPD
Kenya	3	Male	fb0-6	OPD
Kenya	3	Male	fb0-6	OPD
Kenya	6	Male	fb0-6	OPD
Kenya	2	female	fb0-6	OPD
Kenya	4	female	fb0-6	OPD
Kenya	2	female	fb0-6	OPD
Kenya	4	female	fb0-6	OPD
Kenya	4	female	fb0-6	OPD
Kenya	6	Male	fb0-6	OPD
Kenya	3	female	fb0-6	OPD
Kenya	2	female	fb0-6	OPD
Kenya	3	Male	fb0-6	OPD
Kenya	3	Male	fb0-6	OPD
Kenya	5	Male	fb0-6	OPD
Kenya	5	Male	fb0-6	OPD
Kenya	6	female	fb0-6	OPD
Kenya	6	female	fb0-6	OPD
Kenya	6	female	fb0-6	OPD
Kenya	6	female	fb0-6	OPD
Kenya	4	Male	fb0-6	OPD
Kenya	5	Male	fb0-6	OPD
Kenya	3	Male	fb0-6	OPD
Kenya	3	female	fb0-6	OPD
Kenya	4	Male	fb0-6	OPD
Kenya	6	Male	fb0-6	OPD
Kenya	2	female	fb0-6	OPD
Kenya	5	Male	fb0-6	OPD
Kenya	5	Male	fb0-6	OPD
Kenya	5	female	fb0-6	OPD
Kenya	3	Male	fb0-6	OPD
Kenya	6	female	fb0-6	OPD
Kenya	5	Male	fb0-6	OPD
Kenya	3	Male	fb0-6	OPD

Kenya	5	Male	fb0-6	OPD
Kenya	5	Male	fb0-6	OPD
Kenya	6	female	fb0-6	OPD
Kenya	6	Male	fb0-6	OPD
Kenya	5	Male	fb0-6	OPD
Kenya	1	Male	fb0-6	OPD
Kenya	6	female	fb0-6	OPD
Kenya	6	female	fb0-6	OPD
Kenya	3	Male	fb0-6	OPD
Kenya	1	female	fb0-6	OPD
Kenya	4	Male	fb0-6	OPD
Kenya	2	Male	fb0-6	OPD
Kenya	5	Male	fb0-6	OPD
Kenya	4	Male	fb0-6	OPD
Kenya	3	female	fb0-6	OPD
Kenya	2	Male	fb0-6	OPD
Kenya	3	Male	fb0-6	OPD
Kenya	3	Male	fb0-6	OPD
Kenya	6	Male	fb0-6	OPD
Kenya	3	female	fb0-6	OPD
Kenya	2	female	fb0-6	OPD
Kenya	5	Male	fb0-6	OPD
Kenya	2	female	fb0-6	OPD
Kenya	1	Male	fb0-6	OPD
Kenya	2	female	fb0-6	OPD
Kenya	3	Male	fb0-6	OPD
Kenya	4	Male	fb0-6	OPD
Kenya	4	female	fb0-6	OPD
Kenya	4	Male	fb0-6	OPD
Kenya	6	female	fb0-6	OPD
Kenya	6	female	fb0-6	OPD
Kenya	3	female	fb0-6	OPD
Kenya	5	Male	fb0-6	OPD
Kenya	3	female	fb0-6	OPD
Kenya	4	Male	fb0-6	OPD
Kenya	3	female	fb0-6	OPD
Kenya	6	Male	fb0-6	OPD
Kenya	1	Male	fb0-6	OPD
Kenya	3	Male	fb0-6	OPD
Kenya	4	Male	fb0-6	OPD
Kenya	2	female	fb0-6	OPD
Kenya	2	female	fb0-6	OPD
Kenya	5	female	fb0-6	OPD
Kenya	3	Male	fb0-6	OPD
Kenya	6	Male	fb0-6	OPD
Kenya	4	Male	fb0-6	OPD
Kenya	2	female	fb0-6	OPD
Kenya	2	Male	fb0-6	OPD
Kenya	3	Male	fb0-6	OPD
Kenya	6	female	fb0-6	OPD

Kenya	4	female	fb0-6	OPD
Kenya	4	Male	fb0-6	OPD
Kenya	1	Male	fb0-6	OPD
Kenya	3	Male	fb0-6	OPD
Kenya	5	Male	fb0-6	OPD
Kenya	6	Male	fb0-6	OPD
Kenya	3	female	fb0-6	OPD
Kenya	5	female	fb0-6	OPD
Kenya	3	Male	fb0-6	OPD
Kenya	4	female	fb0-6	OPD
Kenya	2	Male	fb0-6	OPD
Kenya	6	Male	fb0-6	OPD
Kenya	2	female	fb0-6	OPD
Kenya	2	Male	fb0-6	OPD
Kenya	4	Male	fb0-6	OPD
Kenya	3	Male	fb0-6	OPD
Kenya	6	Male	fb0-6	OPD
Kenya	3	Male	fb0-6	OPD
Kenya	6	Male	fb0-6	OPD
Kenya	6	female	fb0-6	OPD
Kenya	6	Male	fb0-6	OPD
Kenya	2	female	fb0-6	OPD
Kenya	6	Male	fb0-6	OPD
Kenya	5	Male	fb0-6	OPD
Kenya	6	female	fb0-6	OPD
Kenya	3	female	fb0-6	OPD
Kenya	4	Male	fb0-6	OPD
Kenya	2	Male	fb0-6	OPD
Kenya	2	female	fb0-6	OPD
Kenya	3	Male	fb0-6	OPD
Kenya	2	Male	fb0-6	OPD
Kenya	6	female	fb0-6	OPD
Kenya	3	Male	fb0-6	OPD
Kenya	6	female	fb0-6	OPD
Kenya	4	female	fb0-6	OPD
Kenya	3	Male	fb0-6	OPD
Kenya	3	female	fb0-6	OPD
Kenya	4	Male	fb0-6	OPD
Kenya	4	Male	fb0-6	OPD
Kenya	3	Male	fb0-6	OPD
Kenya	4	Male	fb0-6	OPD
Kenya	4	Male	fb0-6	OPD
Kenya	3	female	fb0-6	OPD
Kenya	3	female	fb0-6	OPD
Kenya	1	Male	fb0-6	OPD
Kenya	6	Male	fb0-6	OPD
Kenya	2	Male	fb0-6	OPD
Kenya	5	Male	fb0-6	OPD
Kenya	4	Male	fb0-6	OPD
Kenya	3	female	fb0-6	OPD

Kenya	6	female	fb0-6	OPD
Kenya	5	Male	fb0-6	OPD
Kenya	1	female	fb0-6	OPD
Kenya	5	Male	fb0-6	OPD
Kenya	6	female	fb0-6	OPD
Kenya	4	Male	fb0-6	OPD
Kenya	6	female	fb0-6	OPD
Kenya	4	Male	fb0-6	OPD
Kenya	6	Male	fb0-6	OPD
Kenya	3	Male	fb0-6	OPD
Kenya	4	Male	fb0-6	OPD
Kenya	3	female	fb0-6	OPD
Kenya	5	female	fb0-6	OPD
Kenya	6	Male	fb0-6	OPD
Kenya	6	female	fb0-6	OPD
Kenya	6	Male	fb0-6	OPD
Kenya	3	Male	fb0-6	OPD
Kenya	3	female	fb0-6	OPD
Kenya	6	female	fb0-6	OPD
Kenya	6	Male	fb0-6	OPD
Kenya	6	Male	fb0-6	OPD
Kenya	4	Male	fb0-6	OPD
Kenya	4	Male	fb0-6	OPD
Kenya	3	female	fb0-6	OPD
Kenya	5	female	fb0-6	OPD
Kenya	4	female	fb0-6	OPD
Kenya	2	female	fb0-6	OPD
Kenya	4	Male	fb0-6	OPD
Kenya	1	Male	fb0-6	OPD
Kenya	3	female	fb0-6	OPD
Kenya	5	female	fb0-6	OPD
Kenya	4	Male	fb0-6	OPD
Kenya	2	female	fb0-6	OPD
Kenya	3	Male	fb0-6	OPD
Kenya	2	Male	fb0-6	OPD
Kenya	1	Male	fb0-6	OPD
Kenya	3	female	fb0-6	OPD
Kenya	3	female	fb0-6	OPD
Kenya	4	Male	fb0-6	OPD
Kenya	6	female	fb0-6	OPD
Kenya	6	female	fb0-6	OPD
Kenya	2	female	fb0-6	OPD
Kenya	2	Male	fb0-6	OPD
Kenya	2	Male	fb0-6	OPD
Kenya	2	female	fb0-6	OPD
Kenya	3	female	fb0-6	OPD
Kenya	6	Male	fb0-6	OPD
Kenya	6	female	fb0-6	OPD
Kenya	2	Male	fb0-6	OPD
Kenya	5	female	fb0-6	OPD

Kenya	6	female	fb0-6	OPD
Kenya	3	female	fb0-6	OPD
Kenya	3	female	fb0-6	OPD
Kenya	4	Male	fb0-6	OPD
Kenya	2	Male	fb0-6	OPD
Kenya	2	female	fb0-6	OPD
Kenya	1	Male	fb0-6	OPD
Kenya	4	female	fb0-6	OPD
Kenya	6	Male	fb0-6	OPD
Kenya	3	Male	fb0-6	OPD
Kenya	3	female	fb0-6	OPD
Kenya	3	female	fb0-6	OPD
Kenya	1	Male	fb0-6	OPD
Kenya	3	female	fb0-6	OPD
Kenya	6	female	fb0-6	OPD
Kenya	11	female	FB 7-59	OPD
Kenya	11	Male	FB 7-59	OPD
Kenya	10	Male	FB 7-59	OPD
Kenya	10	female	FB 7-59	OPD
Kenya	31	female	FB 7-59	OPD
Kenya	25	Male	FB 7-59	OPD
Kenya	15	female	FB 7-59	OPD
Kenya	14	female	FB 7-59	OPD
Kenya	29	Male	FB 7-59	OPD
Kenya	16	Male	FB 7-59	OPD
Kenya	17	Male	FB 7-59	OPD
Kenya	19	female	FB 7-59	OPD
Kenya	6	female	fb0-6	OPD
Kenya	17	Male	FB 7-59	OPD
Kenya	16	Male	FB 7-59	OPD
Kenya	13	Male	FB 7-59	OPD
Kenya	20	female	FB 7-59	OPD
Kenya	39	female	FB 7-59	OPD
Kenya	21	female	FB 7-59	OPD
Kenya	8	Male	FB 7-59	OPD
Kenya	18	female	FB 7-59	OPD
Kenya	13	female	FB 7-59	OPD
Kenya	14	Male	FB 7-59	OPD
Kenya	8	female	FB 7-59	OPD
Kenya	4	female	fb0-6	OPD
Kenya	9	female	FB 7-59	OPD
Kenya	21	Male	FB 7-59	OPD
Kenya	11	Male	FB 7-59	OPD
Kenya	37	Male	FB 7-59	OPD
Kenya	10	Male	FB 7-59	OPD
Kenya	16	female	FB 7-59	OPD
Kenya	32	female	FB 7-59	OPD
Kenya	29	female	FB 7-59	OPD
Kenya	10	Male	FB 7-59	OPD
Kenya	13	Male	FB 7-59	OPD

Kenya	38	Male	FB 7-59	OPD
Kenya	6	female	fb0-6	OPD
Kenya	24	Male	FB 7-59	OPD
Kenya	20	female	FB 7-59	OPD
Kenya	13	female	FB 7-59	OPD
Kenya	15	Male	FB 7-59	OPD
Kenya	8	Male	FB 7-59	OPD
Kenya	11	female	FB 7-59	OPD
Kenya	18	Male	FB 7-59	OPD
Kenya	6	female	fb0-6	OPD
Kenya	47	female	FB 7-59	OPD
Kenya	26	female	FB 7-59	OPD
Kenya	18	female	FB 7-59	OPD
Kenya	8	female	FB 7-59	OPD
Kenya	13	Male	FB 7-59	OPD
Kenya	18	female	FB 7-59	OPD
Kenya	10	female	FB 7-59	OPD
Kenya	27	Male	FB 7-59	OPD
Kenya	24	Male	FB 7-59	OPD
Kenya	13	Male	FB 7-59	OPD
Kenya	19	Male	FB 7-59	OPD
Kenya	15	Male	FB 7-59	OPD
Kenya	21	female	FB 7-59	OPD
Kenya	13	Male	FB 7-59	OPD
Kenya	14	female	FB 7-59	OPD
Kenya	19	Male	FB 7-59	OPD
Kenya	18	Male	FB 7-59	OPD
Kenya	9	female	FB 7-59	OPD
Kenya	12	Male	FB 7-59	OPD
Kenya	16	Male	FB 7-59	OPD
Kenya	8	Male	FB 7-59	OPD
Kenya	8	Male	FB 7-59	OPD
Kenya	17	female	FB 7-59	OPD
Kenya	18	female	FB 7-59	OPD
Kenya	11	female	FB 7-59	OPD
Kenya	12	female	FB 7-59	OPD
Kenya	14	female	FB 7-59	OPD
Kenya	8	Male	FB 7-59	OPD
Kenya	38	female	FB 7-59	OPD
Kenya	13	female	FB 7-59	OPD
Kenya	14	Male	FB 7-59	OPD
Kenya	11	Male	FB 7-59	OPD
Kenya	13	Male	FB 7-59	OPD
Kenya	8	female	FB 7-59	OPD
Kenya	8	female	FB 7-59	OPD
Kenya	6	female	fb0-6	OPD
Kenya	22	Male	FB 7-59	OPD
Kenya	10	female	FB 7-59	OPD
Kenya	12	female	FB 7-59	OPD
Kenya	17	Male	FB 7-59	OPD

Kenya	12	female	FB 7-59	OPD
Kenya	14	female	FB 7-59	OPD
Kenya	16	Male	FB 7-59	OPD
Kenya	13	female	FB 7-59	OPD
Kenya	35	Male	FB 7-59	OPD
Kenya	19	female	FB 7-59	OPD
Kenya	10	female	FB 7-59	OPD
Kenya	9	Male	FB 7-59	OPD
Kenya	21	female	FB 7-59	OPD
Kenya	12	Male	FB 7-59	OPD
Kenya	15	Male	FB 7-59	OPD
Kenya	7	female	FB 7-59	OPD
Kenya	21	Male	FB 7-59	OPD
Kenya	27	Male	FB 7-59	OPD
Kenya	4	Male	fb0-6	OPD
Kenya	27	Male	FB 7-59	OPD
Kenya	42	Male	FB 7-59	OPD
Kenya	15	Male	FB 7-59	OPD
Kenya	4	female	fb0-6	OPD
Kenya	29	Male	FB 7-59	OPD
Kenya	47	Male	FB 7-59	OPD
Kenya	21	Male	FB 7-59	OPD
Kenya	20	Male	FB 7-59	OPD
Kenya	17	female	FB 7-59	OPD
Kenya	6	Male	fb0-6	OPD
Kenya	7	Male	FB 7-59	OPD
Kenya	33	female	FB 7-59	OPD
Kenya	30	Male	FB 7-59	OPD
Kenya	15	Male	FB 7-59	OPD
Kenya	7	Male	FB 7-59	OPD
Kenya	12	female	FB 7-59	OPD
Kenya	18	female	FB 7-59	OPD
Kenya	17	female	FB 7-59	OPD
Kenya	21	Male	FB 7-59	OPD
Kenya	13	Male	FB 7-59	OPD
Kenya	17	Male	FB 7-59	OPD
Kenya	5	Male	fb0-6	OPD
Kenya	14	Male	FB 7-59	OPD
Kenya	7	female	FB 7-59	OPD
Kenya	18	Male	FB 7-59	OPD
Kenya	9	female	FB 7-59	OPD
Kenya	12	Male	FB 7-59	OPD
Kenya	17	Male	FB 7-59	OPD
Kenya	10	female	FB 7-59	OPD
Kenya	10	female	FB 7-59	OPD
Kenya	45	Male	FB 7-59	OPD
Kenya	13	Male	FB 7-59	OPD
Kenya	11	female	FB 7-59	OPD
Kenya	53	Male	FB 7-59	OPD
Kenya	24	Male	FB 7-59	OPD

Kenya	13	female	FB 7-59	OPD
Kenya	35	Male	FB 7-59	OPD
Kenya	32	female	FB 7-59	OPD
Kenya	11	female	FB 7-59	OPD
Kenya	7	female	FB 7-59	OPD
Kenya	21	Male	FB 7-59	OPD
Kenya	3	Male	fb0-6	OPD
Kenya	7	Male	FB 7-59	OPD
Kenya	9	female	FB 7-59	OPD
Kenya	8	female	FB 7-59	OPD
Kenya	7	female	FB 7-59	OPD
Kenya	10	female	FB 7-59	OPD
Kenya	20	Male	FB 7-59	OPD
Kenya	6	female	fb0-6	OPD
Kenya	14	Male	FB 7-59	OPD
Kenya	9	female	FB 7-59	OPD
Kenya	20	Male	FB 7-59	OPD
Kenya	20	female	FB 7-59	OPD
Kenya	14	Male	FB 7-59	OPD
Kenya	17	female	FB 7-59	OPD
Kenya	9	female	FB 7-59	OPD
Kenya	19	female	FB 7-59	OPD
Kenya	8	Male	FB 7-59	OPD
Kenya	45	female	FB 7-59	OPD
Kenya	10	Male	FB 7-59	OPD
Kenya	10	Male	FB 7-59	OPD
Kenya	12	female	FB 7-59	OPD
Kenya	16	Male	FB 7-59	OPD
Kenya	10	Male	FB 7-59	OPD
Kenya	11	female	FB 7-59	OPD
Kenya	15	female	FB 7-59	OPD
Kenya	26	Male	FB 7-59	OPD
Kenya	7	Male	FB 7-59	OPD
Kenya	13	female	FB 7-59	OPD
Kenya	15	Male	FB 7-59	OPD
Kenya	12	female	FB 7-59	OPD
Kenya	10	Male	FB 7-59	OPD
Kenya	8	Male	FB 7-59	OPD
Kenya	30	female	FB 7-59	OPD
Kenya	4	female	fb0-6	OPD
Kenya	25	Male	FB 7-59	OPD
Kenya	10	female	FB 7-59	OPD
Kenya	20	female	FB 7-59	OPD
Kenya	18	Male	FB 7-59	OPD
Kenya	16	Male	FB 7-59	OPD
Kenya	13	Male	FB 7-59	OPD
Kenya	13	female	FB 7-59	OPD
Kenya	29	Male	FB 7-59	OPD
Kenya	13	female	FB 7-59	OPD
Kenya	16	female	FB 7-59	OPD

Kenya	14	Male	FB 7-59	OPD
Kenya	18	Male	FB 7-59	OPD
Kenya	11	Male	FB 7-59	OPD
Kenya	30	female	FB 7-59	OPD
Kenya	17	Male	FB 7-59	OPD
Kenya	5	female	fb0-6	OPD
Kenya	31	female	FB 7-59	OPD
Kenya	20	female	FB 7-59	OPD
Kenya	29	Male	FB 7-59	OPD
Kenya	54	Male	FB 7-59	OPD
Kenya	14	female	FB 7-59	OPD
Kenya	3	Male	fb0-6	OPD
Kenya	15	female	FB 7-59	OPD
Kenya	14	Male	FB 7-59	OPD
Kenya	11	female	FB 7-59	OPD
Kenya	14	female	FB 7-59	OPD
Kenya	11	female	FB 7-59	OPD
Kenya	19	Male	FB 7-59	OPD
Kenya	25	female	FB 7-59	OPD
Kenya	12	female	FB 7-59	OPD
Kenya	8	female	FB 7-59	OPD
Kenya	8	Male	FB 7-59	OPD
Kenya	19	female	FB 7-59	OPD
Kenya	11	female	FB 7-59	OPD
Kenya	10	Male	FB 7-59	OPD
Kenya	10	female	FB 7-59	OPD
Kenya	15	female	FB 7-59	OPD
Kenya	8	Male	FB 7-59	OPD
Kenya	17	female	FB 7-59	OPD
Kenya	33	Male	FB 7-59	OPD
Kenya	20	female	FB 7-59	OPD
Kenya	3	Male	fb0-6	OPD
Kenya	10	Male	FB 7-59	OPD
Kenya	13	Male	FB 7-59	OPD
Kenya	2	Male	fb0-6	OPD
Kenya	11	female	FB 7-59	OPD
Kenya	17	female	FB 7-59	OPD
Kenya	9	Male	FB 7-59	OPD
Kenya	59	Male	FB 7-59	OPD
Kenya	27	female	FB 7-59	OPD
Kenya	21	Male	FB 7-59	OPD
Kenya	18	Male	FB 7-59	OPD
Kenya	13	Male	FB 7-59	OPD
Kenya	6	Male	fb0-6	OPD
Kenya	15	female	FB 7-59	OPD
Kenya	11	female	FB 7-59	OPD
Kenya	13	female	FB 7-59	OPD
Kenya	14	Male	FB 7-59	OPD
Kenya	25	Male	FB 7-59	OPD
Kenya	11	female	FB 7-59	OPD

Kenya	14	female	FB 7-59	OPD
Kenya	10	Male	FB 7-59	OPD
Kenya	13	female	FB 7-59	OPD
Kenya	24	Male	FB 7-59	OPD
Kenya	17	Male	FB 7-59	OPD
Kenya	16	Male	FB 7-59	OPD
Kenya	35	female	FB 7-59	OPD
Kenya	27	Male	FB 7-59	OPD
Kenya	17	Male	FB 7-59	OPD
Kenya	18	Male	FB 7-59	OPD
Kenya	45	female	FB 7-59	OPD
Kenya	17	Male	FB 7-59	OPD
Kenya	14	female	FB 7-59	OPD
Kenya	19	female	FB 7-59	OPD
Kenya	14	female	FB 7-59	OPD
Kenya	23	Male	FB 7-59	OPD
Kenya	14	Male	FB 7-59	OPD
Kenya	16	Male	FB 7-59	OPD
Kenya	14	Male	FB 7-59	OPD
Kenya	13	Male	FB 7-59	OPD
Kenya	15	female	FB 7-59	OPD
Kenya	8	female	FB 7-59	OPD
Kenya	9	Male	FB 7-59	OPD
Kenya	12	Male	FB 7-59	OPD
Kenya	21	Male	FB 7-59	OPD
Kenya	16	female	FB 7-59	OPD
Kenya	12	female	FB 7-59	OPD
Kenya	14	female	FB 7-59	OPD
Kenya	18	female	FB 7-59	OPD
Kenya	14	Male	FB 7-59	OPD
Kenya	9	Male	FB 7-59	OPD
Kenya	14	female	FB 7-59	OPD
Kenya	13	female	FB 7-59	OPD
Kenya	17	Male	FB 7-59	OPD
Kenya	21	Male	FB 7-59	OPD
Kenya	16	Male	FB 7-59	OPD
Kenya	16	Male	FB 7-59	OPD
Kenya	49	Male	FB 7-59	OPD
Kenya	24	female	FB 7-59	OPD
Kenya	29	female	FB 7-59	OPD
Kenya	9	female	FB 7-59	OPD
Kenya	13	Male	FB 7-59	OPD
Kenya	59	Male	FB 7-59	OPD
Kenya	6	Male	fb0-6	OPD
Kenya	20	female	FB 7-59	OPD
Kenya	8	Male	FB 7-59	OPD
Kenya	36	female	FB 7-59	OPD
Kenya	51	Male	FB 7-59	OPD
Kenya	18	Male	FB 7-59	OPD
Kenya	50	Male	FB 7-59	OPD

Kenya	2	female	no movement	OPD
Kenya	9	Male	FB 7-59	OPD
Kenya	10	Male	FB 7-59	OPD
Kenya	25	Male	FB 7-59	OPD
Kenya	40	female	FB 7-59	OPD
Kenya	8	female	FB 7-59	OPD
Kenya	23	Male	FB 7-59	OPD
Kenya	18	female	FB 7-59	OPD
Kenya	13	Male	FB 7-59	OPD
Kenya	6	Male	fb0-6	OPD
Kenya	10	Male	FB 7-59	OPD
Kenya	28	Male	FB 7-59	OPD
Kenya	46	Male	FB 7-59	OPD
Kenya	16	female	FB 7-59	OPD
Kenya	12	female	FB 7-59	OPD
Kenya	15	female	FB 7-59	OPD
Kenya	12	female	FB 7-59	OPD
Kenya	46	female	FB 7-59	OPD
Kenya	9	Male	FB 7-59	OPD
Kenya	16	Male	FB 7-59	OPD
Kenya	52	Male	FB 7-59	OPD
Kenya	16	Male	FB 7-59	OPD
Kenya	6	female	fb0-6	OPD
Kenya	6	female	fb0-6	OPD
Kenya	44	Male	FB 7-59	OPD
Kenya	9	Male	FB 7-59	OPD
Kenya	26	Male	FB 7-59	OPD
Kenya	5	female	fb0-6	OPD
Kenya	56	female	FB 7-59	OPD
Kenya	3	female	fb0-6	OPD
Kenya	12	Male	FB 7-59	OPD
Kenya	15	female	FB 7-59	OPD
Kenya	28	female	FB 7-59	OPD
Kenya	16	female	FB 7-59	OPD
Kenya	34	female	FB 7-59	OPD
Kenya	7	Male	FB 7-59	OPD
Kenya	13	Male	FB 7-59	OPD
Kenya	23	female	FB 7-59	OPD
Kenya	17	female	FB 7-59	OPD
Kenya	46	Male	FB 7-59	OPD
Kenya	39	Male	FB 7-59	OPD
Kenya	18	female	FB 7-59	OPD
Kenya	23	Male	FB 7-59	OPD
Kenya	20	female	not feeding	OPD
Kenya	14	female	FB 7-59	OPD
Kenya	35	Male	FB 7-59	OPD
Kenya	23	Male	FB 7-59	OPD
Kenya	20	Male	FB 7-59	OPD
Kenya	18	Male	FB 7-59	OPD
Kenya	8	female	FB 7-59	OPD

Kenya	15	female	FB 7-59	OPD
Kenya	9	female	FB 7-59	OPD
Kenya	20	female	FB 7-59	OPD
Kenya	7	Male	FB 7-59	OPD
Kenya	16	Male	FB 7-59	OPD
Kenya	9	Male	FB 7-59	OPD
Kenya	16	female	FB 7-59	OPD
Kenya	32	Male	FB 7-59	OPD
Kenya	35	Male	FB 7-59	OPD
Kenya	13	Male	FB 7-59	OPD
Kenya	45	Male	FB 7-59	OPD
Kenya	11	Male	FB 7-59	OPD
Kenya	9	female	FB 7-59	OPD
Kenya	38	Male	FB 7-59	OPD
Kenya	19	female	FB 7-59	OPD
Kenya	9	female	FB 7-59	OPD
Kenya	8	Male	FB 7-59	OPD
Kenya	17	Male	FB 7-59	OPD
Kenya	17	Male	FB 7-59	OPD
Kenya	52	Male	FB 7-59	OPD
Kenya	11	Male	FB 7-59	OPD
Kenya	28	female	FB 7-59	OPD
Kenya	42	Male	FB 7-59	OPD
Kenya	7	Male	FB 7-59	OPD
Kenya	3	Male	fb0-6	OPD
Kenya	24	female	FB 7-59	OPD
Kenya	13	Male	FB 7-59	OPD
Kenya	12	Male	FB 7-59	OPD
Kenya	11	female	FB 7-59	OPD
Kenya	44	Male	FB 7-59	OPD
Kenya	12	Male	FB 7-59	OPD
Kenya	44	female	FB 7-59	OPD
Kenya	16	Male	FB 7-59	OPD
Kenya	8	female	FB 7-59	OPD
Kenya	12	female	FB 7-59	OPD
Kenya	19	female	FB 7-59	OPD
Kenya	13	Male	FB 7-59	OPD
Kenya	24	female	FB 7-59	OPD
Kenya	20	female	FB 7-59	OPD
Kenya	27	Male	FB 7-59	OPD
Kenya	35	Male	FB 7-59	OPD
Kenya	14	Male	FB 7-59	OPD
Kenya	12	female	FB 7-59	OPD
Kenya	13	female	FB 7-59	OPD
Kenya	1	Male	fb0-6	OPD
Kenya	17	female	FB 7-59	OPD
Kenya	14	Male	FB 7-59	OPD
Kenya	26	Male	FB 7-59	OPD
Kenya	17	female	FB 7-59	OPD
Kenya	12	female	FB 7-59	OPD

Kenya	19	female	FB 7-59	OPD
Kenya	39	Male	FB 7-59	OPD
Kenya	30	Male	FB 7-59	OPD
Kenya	12	Male	FB 7-59	OPD
Kenya	7	female	FB 7-59	OPD
Kenya	11	Male	FB 7-59	OPD
Kenya	13	female	FB 7-59	OPD
Kenya	10	female	FB 7-59	OPD
Kenya	49	Male	FB 7-59	OPD
Kenya	21	female	FB 7-59	OPD
Kenya	51	female	FB 7-59	OPD
Kenya	28	female	FB 7-59	OPD
Kenya	11	Male	FB 7-59	OPD
Kenya	6	Male	fb0-6	OPD
Kenya	8	Male	FB 7-59	OPD
Kenya	54	Male	FB 7-59	OPD
Kenya	9	female	FB 7-59	OPD
Kenya	14	female	FB 7-59	OPD
Kenya	51	female	FB 7-59	OPD
Kenya	14	Male	FB 7-59	OPD
Kenya	12	female	FB 7-59	OPD
Kenya	7	Male	FB 7-59	OPD
Kenya	8	female	FB 7-59	OPD
Kenya	26	female	FB 7-59	OPD
Kenya	31	female	FB 7-59	OPD
Kenya	26	Male	FB 7-59	OPD
Kenya	23	female	FB 7-59	OPD
Kenya	14	Male	FB 7-59	OPD
Kenya	3	Male	fb0-6	OPD
Kenya	14	Male	FB 7-59	OPD
Kenya	15	female	FB 7-59	OPD
Kenya	9	Male	FB 7-59	OPD
Kenya	4	Male	fb0-6	OPD
Kenya	20	Male	FB 7-59	OPD
Kenya	3	Male	fb0-6	OPD
Kenya	17	female	FB 7-59	OPD
Kenya	7	female	FB 7-59	OPD
Kenya	43	female	FB 7-59	OPD
Kenya	9	female	FB 7-59	OPD
Kenya	44	female	FB 7-59	OPD
Kenya	8	female	FB 7-59	OPD
Kenya	9	female	FB 7-59	OPD
Kenya	20	female	FB 7-59	OPD
Kenya	16	Male	FB 7-59	OPD
Kenya	21	female	FB 7-59	OPD
Kenya	13	female	FB 7-59	OPD
Kenya	21	female	FB 7-59	OPD
Kenya	29	Male	FB 7-59	OPD
Kenya	10	Male	FB 7-59	OPD
Kenya	21	female	FB 7-59	OPD

Kenya	13	female	FB 7-59	OPD
Kenya	21	female	FB 7-59	OPD
Kenya	21	female	FB 7-59	OPD
Kenya	11	female	FB 7-59	OPD
Kenya	40	Male	FB 7-59	OPD
Kenya	1	Male	fb0-6	OPD
Kenya	27	Male	FB 7-59	OPD
Kenya	17	Male	FB 7-59	OPD
Kenya	3	Male	fb0-6	OPD
Kenya	23	Male	FB 7-59	OPD
Kenya	36	female	FB 7-59	OPD
Kenya	16	female	FB 7-59	OPD
Kenya	1	Male	fb0-6	OPD
Kenya	41	Male	FB 7-59	OPD
Kenya	8	female	FB 7-59	OPD
Kenya	28	Male	FB 7-59	OPD
Kenya	8	female	FB 7-59	OPD
Kenya	16	female	FB 7-59	OPD
Kenya	49	Male	FB 7-59	OPD
Kenya	6	female	fb0-6	OPD
Kenya	16	Male	FB 7-59	OPD
Kenya	24	Male	FB 7-59	OPD
Kenya	15	Male	FB 7-59	OPD
Kenya	34	Male	FB 7-59	OPD
Kenya	14	Male	FB 7-59	OPD
Kenya	16	Male	FB 7-59	OPD
Kenya	36	Male	FB 7-59	OPD
Kenya	26	Male	FB 7-59	OPD
Kenya	7	female	FB 7-59	OPD
Kenya	21	female	FB 7-59	OPD
Kenya	27	female	FB 7-59	OPD
Kenya	10	Male	FB 7-59	OPD
Kenya	31	Male	FB 7-59	OPD
Kenya	49	Male	FB 7-59	OPD
Kenya	8	female	FB 7-59	OPD
Kenya	1	female	fb0-6	OPD
Kenya	15	Male	FB 7-59	OPD
Kenya	8	Male	FB 7-59	OPD
Kenya	12	Male	FB 7-59	OPD
Kenya	30	female	FB 7-59	OPD
Kenya	21	Male	FB 7-59	OPD
Kenya	21	female	FB 7-59	OPD
Kenya	20	Male	FB 7-59	OPD
Kenya	4	Male	fb0-6	OPD
Kenya	12	Male	FB 7-59	OPD
Kenya	19	Male	FB 7-59	OPD
Kenya	51	female	FB 7-59	OPD
Kenya	7	female	FB 7-59	OPD
Kenya	21	female	FB 7-59	OPD
Kenya	25	Male	FB 7-59	OPD

Kenya	10	Male	FB 7-59	OPD
Kenya	10	Male	FB 7-59	OPD
Kenya	21	female	FB 7-59	OPD
Kenya	57	female	FB 7-59	OPD
Kenya	30	Male	FB 7-59	OPD
Kenya	22	female	FB 7-59	OPD
Kenya	52	Male	FB 7-59	OPD
Kenya	14	Male	FB 7-59	OPD
Kenya	21	Male	FB 7-59	OPD
Kenya	46	Male	FB 7-59	OPD
Kenya	36	Male	FB 7-59	OPD
Kenya	57	Male	FB 7-59	OPD
Kenya	49	female	FB 7-59	OPD
Kenya	15	female	FB 7-59	OPD
Kenya	21	female	FB 7-59	OPD
Kenya	4	female	fb0-6	OPD
Kenya	23	female	FB 7-59	OPD
Kenya	14	Male	FB 7-59	OPD
Kenya	39	Male	FB 7-59	OPD
Kenya	7	Male	FB 7-59	OPD
Kenya	40	Male	FB 7-59	OPD
Kenya	16	Male	FB 7-59	OPD
Kenya	10	female	FB 7-59	OPD
Kenya	11	Male	FB 7-59	OPD
Kenya	41	Male	FB 7-59	OPD
Kenya	17	Male	FB 7-59	OPD
Kenya	16	Male	FB 7-59	OPD
Kenya	10	female	FB 7-59	OPD
Kenya	2	female	fb0-6	OPD
Kenya	11	female	FB 7-59	OPD
Kenya	16	female	FB 7-59	OPD
Kenya	14	Male	FB 7-59	OPD
Kenya	8	Male	FB 7-59	OPD
Kenya	6	female	fb0-6	OPD
Kenya	26	female	FB 7-59	OPD
Kenya	43	female	FB 7-59	OPD
Kenya	15	female	FB 7-59	OPD
Kenya	19	female	FB 7-59	OPD
Kenya	14	Male	FB 7-59	OPD
Kenya	6	female	fb0-6	OPD
Kenya	10	female	FB 7-59	OPD
Kenya	8	female	FB 7-59	OPD
Kenya	56	female	FB 7-59	OPD
Kenya	16	Male	FB 7-59	OPD
Kenya	24	female	FB 7-59	OPD
Kenya	44	female	FB 7-59	OPD
Kenya	10	female	FB 7-59	OPD
Kenya	13	Male	FB 7-59	OPD
Kenya	18	Male	FB 7-59	OPD
Kenya	7	Male	FB 7-59	OPD

Kenya	10	Male	FB 7-59	OPD
Kenya	25	female	FB 7-59	OPD
Kenya	35	Male	FB 7-59	OPD
Kenya	35	Male	FB 7-59	OPD
Kenya	16	female	FB 7-59	OPD
Kenya	13	Male	FB 7-59	OPD
Kenya	21	female	FB 7-59	OPD
Kenya	13	Male	FB 7-59	OPD
Kenya	10	female	FB 7-59	OPD
Kenya	13	female	FB 7-59	OPD
Kenya	5	female	fb0-6	OPD
Kenya	19	Male	FB 7-59	OPD
Kenya	22	female	FB 7-59	OPD
Kenya	25	female	FB 7-59	OPD
Kenya	9	female	FB 7-59	OPD
Kenya	26	female	FB 7-59	OPD
Kenya	14	female	FB 7-59	OPD
Kenya	17	Male	FB 7-59	OPD
Kenya	17	Male	FB 7-59	OPD
Kenya	13	female	FB 7-59	OPD
Kenya	15	Male	FB 7-59	OPD
Kenya	34	Male	FB 7-59	OPD
Kenya	11	Male	FB 7-59	OPD
Kenya	10	Male	FB 7-59	OPD
Kenya	21	female	FB 7-59	OPD
Kenya	11	female	FB 7-59	OPD
Kenya	14	Male	FB 7-59	OPD
Kenya	8	Male	FB 7-59	OPD
Kenya	13	female	FB 7-59	OPD
Kenya	8	Male	FB 7-59	OPD
Kenya	11	female	FB 7-59	OPD
Kenya	4	female	fb0-6	OPD
Kenya	10	female	FB 7-59	OPD
Kenya	22	Male	FB 7-59	OPD
Kenya	13	Male	FB 7-59	OPD
Kenya	12	Male	FB 7-59	OPD
Kenya	22	female	FB 7-59	OPD
Kenya	24	Male	FB 7-59	OPD
Kenya	9	female	FB 7-59	OPD
Kenya	30	Male	FB 7-59	OPD
Kenya	1	female	fb0-6	OPD
Kenya	1	Male	fb0-6	OPD
Kenya	12	Male	FB 7-59	OPD
Kenya	36	female	FB 7-59	OPD
Kenya	16	female	FB 7-59	OPD
Kenya	38	female	FB 7-59	OPD
Kenya	9	Male	FB 7-59	OPD
Kenya	24	Male	FB 7-59	OPD
Kenya	17	female	FB 7-59	OPD
Kenya	40	female	FB 7-59	OPD

Kenya	10	Male	FB 7-59	OPD
Kenya	13	Male	FB 7-59	OPD
Kenya	9	female	FB 7-59	OPD
Kenya	13	Male	FB 7-59	OPD
Kenya	21	Male	FB 7-59	OPD
Kenya	25	Male	FB 7-59	OPD
Kenya	16	Male	FB 7-59	OPD
Kenya	18	Male	FB 7-59	OPD
Kenya	57	Male	FB 7-59	OPD
Kenya	33	female	FB 7-59	OPD
Kenya	42	female	FB 7-59	OPD
Kenya	12	Male	FB 7-59	OPD
Kenya	25	female	FB 7-59	OPD
Kenya	11	female	FB 7-59	OPD
Kenya	18	Male	FB 7-59	OPD
Kenya	18	female	FB 7-59	OPD
Kenya	36	female	FB 7-59	OPD
Kenya	7	female	FB 7-59	OPD
Kenya	20	female	FB 7-59	OPD
Kenya	17	Male	FB 7-59	OPD
Kenya	39	Male	FB 7-59	OPD
Kenya	15	Male	FB 7-59	OPD
Kenya	9	female	FB 7-59	OPD
Kenya	9	Male	FB 7-59	OPD
Kenya	9	Male	FB 7-59	OPD
Kenya	32	Male	FB 7-59	OPD
Kenya	18	female	FB 7-59	OPD
Kenya	14	Male	FB 7-59	OPD
Kenya	8	Male	FB 7-59	OPD
Kenya	18	Male	FB 7-59	OPD
Kenya	32	female	FB 7-59	OPD
Kenya	17	female	FB 7-59	OPD
Kenya	11	female	FB 7-59	OPD
Kenya	30	Male	FB 7-59	OPD
Kenya	30	female	FB 7-59	OPD
Kenya	19	female	FB 7-59	OPD
Kenya	23	Male	FB 7-59	OPD
Kenya	8	Male	FB 7-59	OPD
Kenya	16	female	FB 7-59	OPD
Kenya	24	female	FB 7-59	OPD
Kenya	8	female	FB 7-59	OPD
Kenya	36	female	FB 7-59	OPD
Kenya	19	Male	FB 7-59	OPD
Kenya	22	Male	FB 7-59	OPD
Kenya	21	female	FB 7-59	OPD
Kenya	22	female	FB 7-59	OPD
Kenya	21	Male	FB 7-59	OPD
Kenya	22	female	FB 7-59	OPD
Kenya	23	female	FB 7-59	OPD
Kenya	17	Male	FB 7-59	OPD

Kenya	23	Male	FB 7-59	OPD
Kenya	19	Male	FB 7-59	OPD
Kenya	17	Male	FB 7-59	OPD
Kenya	12	female	FB 7-59	OPD
Kenya	12	female	FB 7-59	OPD
Kenya	15	Male	FB 7-59	OPD
Kenya	37	Male	FB 7-59	OPD
Kenya	11	female	FB 7-59	OPD
Kenya	9	female	FB 7-59	OPD
Kenya	13	Male	FB 7-59	OPD
Kenya	19	Male	FB 7-59	OPD
Kenya	28	Male	FB 7-59	OPD
Kenya	9	female	FB 7-59	OPD
Kenya	10	Male	FB 7-59	OPD
Kenya	8	Male	FB 7-59	OPD
Kenya	38	Male	FB 7-59	OPD
Kenya	15	Male	FB 7-59	OPD
Kenya	26	Male	FB 7-59	OPD
Kenya	16	Male	FB 7-59	OPD
Kenya	14	female	FB 7-59	OPD
Kenya	18	Male	FB 7-59	OPD
Kenya	7	Male	FB 7-59	OPD
Kenya	51	Male	FB 7-59	OPD
Kenya	16	Male	FB 7-59	OPD
Kenya	10	Male	FB 7-59	OPD
Kenya	14	Male	FB 7-59	OPD
Kenya	10	Male	FB 7-59	OPD
Kenya	21	female	FB 7-59	OPD
Kenya	35	female	FB 7-59	OPD
Kenya	51	female	FB 7-59	OPD
Kenya	42	female	FB 7-59	OPD
Kenya	22	female	FB 7-59	OPD
Kenya	12	Male	FB 7-59	OPD
Kenya	22	Male	FB 7-59	OPD
Kenya	19	female	FB 7-59	OPD
Kenya	19	Male	FB 7-59	OPD
Kenya	19	Male	FB 7-59	OPD
Kenya	13	Male	FB 7-59	OPD
Kenya	10	Male	FB 7-59	OPD
Kenya	4	female	fb0-6	OPD
Kenya	22	female	FB 7-59	OPD
Kenya	9	female	FB 7-59	OPD
Kenya	7	Male	FB 7-59	OPD
Kenya	6	Male	fb0-6	OPD
Kenya	6	female	fb0-6	OPD
Kenya	10	Male	FB 7-59	OPD
Kenya	19	Male	FB 7-59	OPD
Kenya	13	Male	FB 7-59	OPD
Kenya	10	female	FB 7-59	OPD
Kenya	6	Male	fb0-6	OPD

Kenya	9	female	FB 7-59	OPD
Kenya	19	Male	FB 7-59	OPD
Kenya	13	female	FB 7-59	OPD
Kenya	19	Male	FB 7-59	OPD
Kenya	10	Male	FB 7-59	OPD
Kenya	10	Male	FB 7-59	OPD
Kenya	8	female	FB 7-59	OPD
Kenya	8	Male	FB 7-59	OPD
Kenya	2	female	fb0-6	OPD
Kenya	30	Male	FB 7-59	OPD
Kenya	30	Male	FB 7-59	OPD
Kenya	50	Male	FB 7-59	OPD
Kenya	7	female	FB 7-59	OPD
Kenya	13	Male	FB 7-59	OPD
Kenya	16	female	FB 7-59	OPD
Kenya	21	Male	FB 7-59	OPD
Kenya	18	Male	FB 7-59	OPD
Kenya	10	female	FB 7-59	OPD
Kenya	7	female	FB 7-59	OPD
Kenya	38	female	FB 7-59	OPD
Kenya	17	Male	FB 7-59	OPD
Kenya	18	Male	FB 7-59	OPD
Kenya	15	Male	FB 7-59	OPD
Kenya	25	female	FB 7-59	OPD
Kenya	16	Male	FB 7-59	OPD
Kenya	14	Male	FB 7-59	OPD
Kenya	27	Male	FB 7-59	OPD
Kenya	9	Male	FB 7-59	OPD
Kenya	30	Male	FB 7-59	OPD
Kenya	7	Male	FB 7-59	OPD
Kenya	58	Male	FB 7-59	OPD
Kenya	15	female	FB 7-59	OPD
Kenya	41	Male	FB 7-59	OPD
Kenya	8	Male	FB 7-59	OPD
Kenya	28	female	FB 7-59	OPD
Ibadan	4	female	fever	OPD
Ibadan	3	Male	fever	OPD
Ibadan	1	Male	>1 CSI	OPD
Ibadan	5	Male	>1 CSI	OPD
Ibadan	1	Male	>1 CSI	OPD
Ibadan	2	female	>1 CSI	OPD
Ibadan	3	female	fever	OPD
Ibadan	2	female	fever	OPD
Ibadan	3	Male	>1 CSI	OPD
Ibadan	2	Male	fever	OPD
Ibadan	1	Male	>1 CSI	OPD
Ibadan	2	Male	>1 CSI	OPD
Ibadan	2	Male	poor feeding	OPD
Ibadan	2	Male	poor feeding	OPD
Ibadan	2	Male	poor feeding	OPD

Ibadan	4	female	fever	OPD
Ibadan	4	Male	fever	OPD
Ibadan	6	Male	fever	OPD
Ibadan	3	Male	fever	OPD
Ibadan	2	Male	fever	OPD
Ibadan	1	female	>1 CSI	OPD
Ibadan	4	female	fever	OPD
Ibadan	2	Male	fever	OPD
Ibadan	5	female	>1 CSI	OPD
Ibadan	5	female	fever	OPD
Ibadan	3	female	>1 CSI	OPD
Ibadan	1	Male	LCI	OPD
Ibadan	2	female	fever	OPD
Ibadan	3	female	poor feeding	OPD
Ibadan	1	Male	>1 CSI	OPD
Ibadan	1	female	Hypo	OPD
Ibadan	1	Male	Hypo	OPD
Ibadan	6	Male	fever	OPD
Ibadan	4	female	fever	OPD
Ibadan	2	Male	fever	OPD
Ibadan	5	female	fever	OPD
Ibadan	1	Male	fever	OPD
Ibadan	4	female	fever	OPD
Ibadan	2	female	fever	OPD
Ibadan	1	Male	fever	OPD
Ibadan	3	Male	fever	OPD
Ibadan	1	female	fever	OPD
Ibadan	6	Male	fever	OPD
Ibadan	6	Male	fever	OPD
Ibadan	6	Male	fever	OPD
Ibadan	6	female	fever	OPD
Ibadan	2	Male	fever	OPD
Ibadan	2	Male	fever	OPD
Ibadan	1	female	fever	OPD
Ibadan	1	Male	Hypo	OPD
Ibadan	1	Male	Hypo	OPD
Ibadan	3	Male	>1 CSI	OPD
Ibadan	4	Male	poor feeding	OPD
Ibadan	3	female	poor feeding	OPD
Ibadan	3	Male	fever	OPD
Ibadan	1	Male	LCI	OPD
Ibadan	3	female	LCI	OPD
Ibadan	2	female	fever	OPD
Ibadan	3	Male	fever	OPD
Ibadan	3	female	fever	OPD
Ibadan	1	female	fever	OPD
Ibadan	3	Male	fever	OPD
Ibadan	2	female	fever	OPD
Ibadan	2	Male	fever	OPD
Ibadan	6	female	poor feeding	OPD

Ibadan	3	Male	fever	OPD
Ibadan	3	female	fever	OPD
Ibadan	1	female	LCI	OPD
Ibadan	3	Male	fever	OPD
Ibadan	3	female	LCI	OPD
Ibadan	6	female	fever	OPD
Ibadan	1	female	fever	OPD
Ibadan	2	Male	fever	OPD
Ibadan	4	Male	fever	OPD
Ibadan	3	female	fever	OPD
Ibadan	4	female	LCI	OPD
Ibadan	2	Male	LCI	OPD
Ibadan	1	Male	LCI	OPD
Ibadan	1	Male	fever	OPD
Ibadan	1	Male	fever	OPD
Ibadan	2	female	fever	OPD
Ibadan	5	Male	fever	OPD
Ibadan	2	female	fever	OPD
Ibadan	1	female	fever	OPD
Ibadan	1	female	>1 CSI	OPD
Ibadan	1	Male	fever	OPD
Ibadan	2	Male	fever	OPD
Ibadan	2	Male	fever	OPD
Ibadan	2	female	fever	OPD
Ibadan	2	Male	fever	OPD
Ibadan	1	female	fever	OPD
Ibadan	3	female	fever	OPD
Ibadan	3	Male	LCI	OPD
Ibadan	3	female	LCI	OPD
Ibadan	2	Male	fever	OPD
Ibadan	2	female	fever	OPD
Ibadan	4	female	fever	OPD
Ibadan	3	Male	fever	OPD
Ibadan	5	Male	poor feeding	OPD
Ibadan	5	female	fever	OPD
Ibadan	4	Male	fever	OPD
Ibadan	1	female	fever	OPD
Ibadan	3	female	fever	OPD
Ibadan	2	female	fever	OPD
Ibadan	1	Male	fever	OPD
Ibadan	2	Male	fever	OPD
Ibadan	2	Male	>1 CSI	OPD
Ibadan	2	female	fever	OPD
Ibadan	2	female	fever	OPD
Ibadan	1	female	fever	OPD
Ibadan	2	female	fever	OPD
Ibadan	3	female	fever	OPD
Ibadan	2	female	fever	OPD
Ibadan	1	Male	fever	OPD
Ibadan	1	Male	fever	OPD

Ibadan	3	female	fever	OPD
Ibadan	4	Male	fever	OPD
Ibadan	3	female	fever	OPD
Ibadan	5	Male	fever	OPD
Ibadan	1	Male	fever	OPD
Ibadan	2	Male	fever	OPD
Ibadan	4	Male	fever	OPD
Ibadan	4	Male	fever	OPD
Ibadan	4	female	fever	OPD
Ibadan	4	female	fever	OPD
Ibadan	2	female	fever	OPD
Ibadan	2	female	fever	OPD
Ibadan	1	female	fever	OPD
Ibadan	2	female	fever	OPD
Ibadan	2	Male	fever	OPD
Ibadan	4	Male	fever	OPD
Ibadan	5	female	fever	OPD
Ibadan	3	female	>1 CSI	OPD
Ibadan	4	female	fever	OPD
Ibadan	3	Male	fever	OPD
Ibadan	4	Male	LCI	OPD
Ibadan	4	female	fever	OPD
Ibadan	1	Male	fever	OPD
Ibadan	1	Male	fever	OPD
Ibadan	3	female	fever	OPD
Ibadan	1	female	fever	OPD
Ibadan	6	Male	fever	OPD
Ibadan	4	female	fever	OPD
Ibadan	2	female	fever	OPD
Ibadan	1	female	fever	OPD
Ibadan	4	Male	LCI	OPD
Ibadan	4	female	fever	OPD
Ibadan	6	Male	LCI	OPD
Ibadan	2	Male	>1 CSI	OPD
Ibadan	6	female	LCI	OPD
Ibadan	1	female	LCI	OPD
Ibadan	1	Male	LCI	OPD
Ibadan	3	female	LCI	OPD
Ibadan	3	female	fever	OPD
Ibadan	3	Male	LCI	OPD
Ibadan	1	Male	LCI	OPD
Ibadan	3	female	fever	OPD
Ibadan	3	female	>1 CSI	OPD
Ibadan	1	Male	Hypo	OPD
Ibadan	1	female	>1 CSI	OPD
Ibadan	3	female	poor feeding	OPD
Ibadan	2	Male	LCI	OPD
Ibadan	6	Male	LCI	OPD
Ibadan	6	female	LCI	OPD
Ibadan	5	Male	LCI	OPD

Ibadan	4	Male	LCI	OPD
Ibadan	6	Male	LCI	OPD
Ibadan	4	Male	LCI	OPD
Ibadan	6	female	>1 CSI	OPD
Ibadan	3	Male	fever	OPD
Ibadan	6	Male	>1 CSI	OPD
Ibadan	3	Male	fever	OPD
Ibadan	2	Male	fever	OPD
Ibadan	3	female	fever	OPD
Ibadan	6	female	fever	OPD
Ibadan	1	female	fever	OPD
Ibadan	5	female	LCI	OPD
Ibadan	3	Male	poor feeding	OPD
Ibadan	2	female	fever	OPD
Ibadan	5	Male	fever	OPD
Ibadan	3	Male	>1 CSI	OPD
Ibadan	4	Male	poor feeding	OPD
Ibadan	5	Male	>1 CSI	OPD
Ibadan	2	female	fever	OPD
Ibadan	2	Male	LCI	OPD
Ibadan	6	Male	LCI	OPD
Ibadan	6	Male	LCI	OPD
Ibadan	4	female	LCI	OPD
Ibadan	2	Male	>1 CSI	OPD
Ibadan	1	female	poor feeding	OPD
Ibadan	2	Male	LCI	OPD
Ibadan	1	female	movement on stimula	OPD
Ibadan	4	female	fever	OPD
Ibadan	1	Male	Hypo	OPD
Ibadan	1	Male	movement on stimula	OPD
Ibadan	6	Male	poor feeding	OPD
Ibadan	4	Male	LCI	OPD
Ibadan	1	female	>1 CSI	OPD
Ibadan	4	female	fever	OPD
Ibadan	1	Male	poor feeding	OPD
Ibadan	1	Male	fever	OPD
Ibadan	1	Male	fever	OPD
Ibadan	4	Male	LCI	OPD
Ibadan	1	Male	LCI	OPD
Ibadan	4	female	LCI	OPD
Ibadan	6	Male	LCI	OPD
Ibadan	6	Male	fever	OPD
Ibadan	1	Male	fever	OPD
Ibadan	2	female	fever	OPD
Ibadan	3	female	LCI	OPD
Ibadan	4	Male	fever	OPD
Ibadan	5	Male	fever	OPD
Ibadan	1	female	fever	OPD
Ibadan	3	Male	fever	OPD
Ibadan	5	female	fever	OPD

Ibadan	3	female	fever	OPD
Ibadan	1	female	LCI	OPD
Ibadan	1	Male	LCI	OPD
Ibadan	3	female	fever	OPD
Ibadan	1	Male	LCI	OPD
Ibadan	4	female	fever	OPD
Ibadan	4	female	fever	OPD
Ibadan	1	female	fever	OPD
Ibadan	2	Male	fever	OPD
Ibadan	2	Male	fever	OPD
Ibadan	2	Male	fever	OPD
Ibadan	2	Male	poor feeding	OPD
Ibadan	2	female	fever	OPD
Ibadan	2	Male	fever	OPD
Ibadan	2	female	fever	OPD
Ibadan	2	female	fever	OPD
Ibadan	1	female	fever	OPD
Ibadan	1	Male	fever	OPD
Ibadan	1	female	fever	OPD
Ibadan	2	Male	LCI	OPD
Ibadan	2	Male	fever	OPD
Ibadan	4	Male	LCI	OPD
Ibadan	4	Male	poor feeding	OPD
Ibadan	4	Male	>1 CSI	OPD
Ibadan	5	female	fever	OPD
Ibadan	3	Male	fever	OPD
Ibadan	3	female	fever	OPD
Ibadan	6	Male	LCI	OPD
Ibadan	2	Male	fever	OPD
Ibadan	4	female	fever	OPD
Ibadan	1	female	LCI	OPD
Ibadan	1	Male	fever	OPD
Ibadan	1	Male	fever	OPD
Ibadan	4	Male	fever	OPD
Ibadan	2	female	fever	OPD
Ibadan	1	female	fever	OPD
Ibadan	1	Male	fever	OPD
Ibadan	1	female	fever	OPD
Ibadan	3	female	fever	OPD
Ibadan	1	female	LCI	OPD
Ibadan	4	female	fever	OPD
Ibadan	5	Male	fever	OPD
Ibadan	6	Male	fever	OPD
Ibadan	4	female	fever	OPD
Ibadan	6	female	fever	OPD
Ibadan	3	female	fever	OPD
Ibadan	2	Male	fever	OPD
Ibadan	1	female	fever	OPD
Ibadan	3	Male	fever	OPD
Ibadan	3	Male	fever	OPD

Ibadan	4	female	LCI	OPD
Ibadan	2	Male	fever	OPD
Ibadan	5	female	fever	OPD
Ibadan	5	Male	LCI	OPD
Ibadan	2	female	fever	OPD
Ibadan	1	Male	LCI	OPD
Ibadan	5	female	fever	OPD
Ibadan	1	Male	fever	OPD
Ibadan	3	Male	fever	OPD
Ibadan	1	female	fever	OPD
Ibadan	6	female	fever	OPD
Ibadan	1	female	LCI	OPD
Ibadan	3	female	fever	OPD
Ibadan	3	female	fever	OPD
Ibadan	1	Male	fever	OPD
Ibadan	14	Male	fever	OPD
Ibadan	27	Male	fever	OPD
Ibadan	23	female	LCI	OPD
Ibadan	30	Male	fever	OPD
Ibadan	13	Male	fever	OPD
Ibadan	35	female	>1 CSI	OPD
Ibadan	47	female	LCI	OPD
Ibadan	37	female	LCI	OPD
Ibadan	15	Male	poor feeding	OPD
Ibadan	28	Male	fever	OPD
Ibadan	12	female	fever	OPD
Ibadan	16	female	fever	OPD
Ibadan	23	female	fever	OPD
Ibadan	8	Male	fever	OPD
Ibadan	18	Male	LCI	OPD
Ibadan	13	female	fever	OPD
Ibadan	52	female	fever	OPD
Ibadan	16	Male	fever	OPD
Ibadan	12	Male	LCI	OPD
Ibadan	12	female	fever	OPD
Ibadan	36	female	fever	OPD
Ibadan	28	female	LCI	OPD
Ibadan	28	Male	fever	OPD
Ibadan	49	Male	fever	OPD
Ibadan	7	Male	fever	OPD
Ibadan	13	Male	Hypo	OPD
Ibadan	9	female	>1 CSI	OPD
Ibadan	13	female	fever	OPD
Ibadan	18	Male	LCI	OPD
Ibadan	36	Male	fever	OPD
Ibadan	19	female	LCI	OPD
Ibadan	19	Male	LCI	OPD
Ibadan	39	female	fever	OPD
Ibadan	59	female	fever	OPD
Ibadan	24	Male	LCI	OPD

Ibadan	13	Male	LCI	OPD
Ibadan	41	Male	>1 CSI	OPD
Ibadan	28	Male	>1 CSI	OPD
Ibadan	41	female	fever	OPD
Ibadan	38	female	fever	OPD
Ibadan	21	Male	>1 CSI	OPD
Ibadan	7	female	>1 CSI	OPD
Ibadan	46	Male	fever	OPD
Ibadan	45	female	fever	OPD
Ibadan	18	Male	poor feeding	OPD
Ibadan	31	female	LCI	OPD
Ibadan	54	Male	poor feeding	OPD
Ibadan	21	female	LCI	OPD
Ibadan	28	Male	LCI	OPD
Ibadan	22	Male	LCI	OPD
Ibadan	24	Male	LCI	OPD
Ibadan	52	Male	>1 CSI	OPD
Ibadan	19	Male	LCI	OPD
Ibadan	48	Male	LCI	OPD
Ibadan	21	Male	LCI	OPD
Ibadan	12	female	poor feeding	OPD
Ibadan	20	Male	>1 CSI	OPD
Ibadan	27	Male	fever	OPD
Ibadan	24	Male	LCI	OPD
Ibadan	39	Male	fever	OPD
Ibadan	22	Male	fever	OPD
Ibadan	26	female	>1 CSI	OPD
Ibadan	27	female	>1 CSI	OPD
Ibadan	25	Male	fever	OPD
Ibadan	28	Male	>1 CSI	OPD
Ibadan	12	Male	fever	OPD
Ibadan	12	Male	fever	OPD
Ibadan	13	Male	fever	OPD
Ibadan	42	Male	fever	OPD
Ibadan	9	Male	fever	OPD
Ibadan	41	female	fever	OPD
Ibadan	30	Male	fever	OPD
Ibadan	48	female	fever	OPD
Ibadan	14	female	fever	OPD
Ibadan	7	female	fever	OPD
Ibadan	9	Male	fever	OPD
Ibadan	58	female	LCI	OPD
Ibadan	58	Male	fever	OPD
Ibadan	48	Male	fever	OPD
Ibadan	10	Male	fever	OPD
Ibadan	37	Male	poor feeding	OPD
Ibadan	48	female	LCI	OPD
Ibadan	29	female	poor feeding	OPD
Ibadan	35	Male	fever	OPD
Ibadan	28	female	LCI	OPD

Ibadan	35	female	fever	OPD
Ibadan	24	Male	>1 CSI	OPD
Ibadan	59	female	>1 CSI	OPD
Ibadan	33	female	fever	OPD
Ibadan	37	Male	LCI	OPD
Ibadan	43	Male	LCI	OPD
Ibadan	20	female	LCI	OPD
Ibadan	18	Male	fever	OPD
Ibadan	26	Male	fever	OPD
Ibadan	59	female	fever	OPD
Ibadan	14	Male	LCI	OPD
Ibadan	13	female	LCI	OPD
Ibadan	15	Male	poor feeding	OPD
Ibadan	25	Male	poor feeding	OPD
Ibadan	29	Male	LCI	OPD
Ibadan	10	Male	>1 CSI	OPD
Ibadan	29	female	LCI	OPD
Ibadan	29	female	LCI	OPD
Ibadan	17	Male	LCI	OPD
Ibadan	39	female	LCI	OPD
Ibadan	15	female	poor feeding	OPD
Ibadan	7	female	>1 CSI	OPD
Ibadan	7	female	fever	OPD
Ibadan	44	Male	LCI	OPD
Ibadan	19	Male	LCI	OPD
Ibadan	54	Male	LCI	OPD
Ibadan	10	Male	LCI	OPD
Ibadan	8	Male	fever	OPD
Ibadan	19	female	LCI	OPD
Ibadan	43	Male	fever	OPD
Ibadan	35	female	>1 CSI	OPD
Ibadan	9	female	fever	OPD
Ibadan	18	Male	LCI	OPD
Ibadan	38	Male	LCI	OPD
Ibadan	53	female	fever	OPD
Ibadan	11	Male	fever	OPD
Ibadan	20	Male	LCI	OPD
Ibadan	18	Male	fever	OPD
Ibadan	10	female	fever	OPD
Ibadan	43	female	fever	OPD
Ibadan	21	Male	fever	OPD
Ibadan	14	Male	fever	OPD
Ibadan	20	female	LCI	OPD
Ibadan	17	Male	LCI	OPD
Ibadan	14	Male	fever	OPD
Ibadan	39	female	fever	OPD
Ibadan	8	Male	fever	OPD
Ibadan	47	female	LCI	OPD
Ibadan	23	Male	fever	OPD
Ibadan	15	Male	fever	OPD

Ibadan	32	female	fever	OPD
Ibadan	17	female	fever	OPD
Ibadan	7	female	fever	OPD
Ibadan	26	Male	fever	OPD
Ibadan	29	female	LCI	OPD
Ibadan	32	Male	fever	OPD
Ibadan	49	Male	LCI	OPD
Ibadan	11	Male	>1 CSI	OPD
Ibadan	19	Male	LCI	OPD
Ibadan	18	Male	LCI	OPD
Ibadan	28	Male	LCI	OPD
Ibadan	14	female	fever	OPD
Ibadan	34	Male	LCI	OPD
Ibadan	25	Male	>1 CSI	OPD
Ibadan	9	Male	fever	OPD
Ibadan	25	Male	LCI	OPD
Ibadan	28	Male	LCI	OPD
Ibadan	6	Male	fever	OPD
Ibadan	15	female	fever	OPD
Ibadan	14	female	fever	OPD
Ibadan	9	Male	fever	OPD
Ibadan	29	female	fever	OPD
Ibadan	13	Male	fever	OPD
Ibadan	22	Male	fever	OPD
Ibadan	23	Male	fever	OPD
Ibadan	43	female	LCI	OPD
Ibadan	43	female	LCI	OPD
Ibadan	21	Male	LCI	OPD
Ibadan	19	female	LCI	OPD
Ibadan	42	Male	LCI	OPD
Ibadan	42	Male	LCI	OPD
Ibadan	30	Male	>1 CSI	OPD
Ibadan	20	Male	LCI	OPD
Ibadan	24	Male	>1 CSI	OPD
Ibadan	14	Male	LCI	OPD
Ibadan	7	Male	fever	OPD
Ibadan	10	Male	fever	OPD
Ibadan	42	female	LCI	OPD
Ibadan	7	Male	fever	OPD
Ibadan	32	Male	LCI	OPD
Ibadan	11	Male	fever	OPD
Ibadan	26	female	fever	OPD
Ibadan	24	female	LCI	OPD
Ibadan	16	female	LCI	OPD
Ibadan	21	female	LCI	OPD
Ibadan	41	female	LCI	OPD
Ibadan	29	female	>1 CSI	OPD
Ibadan	52	female	LCI	OPD
Ibadan	24	female	LCI	OPD
Ibadan	38	female	LCI	OPD

Ibadan	56	Male	fever	OPD
Ibadan	28	Male	fever	OPD
Ibadan	59	Male	fever	OPD
Ibadan	14	female	fever	OPD
Ibadan	9	Male	LCI	OPD
Ibadan	48	female	fever	OPD
Ibadan	14	Male	LCI	OPD
Ibadan	18	female	fever	OPD
Ibadan	36	female	fever	OPD
Ibadan	8	Male	fever	OPD
Ibadan	19	female	fever	OPD
Ibadan	29	Male	fever	OPD
Ibadan	11	Male	LCI	OPD
Ibadan	41	female	fever	OPD
Ibadan	16	Male	>1 CSI	OPD
Ibadan	21	Male	LCI	OPD
Ibadan	20	Male	LCI	OPD
Ibadan	33	Male	LCI	OPD
Ibadan	33	Male	LCI	OPD
Ibadan	13	female	>1 CSI	OPD
Ibadan	20	female	LCI	OPD
Ibadan	10	Male	fever	OPD
Ibadan	58	female	>1 CSI	OPD
Ibadan	54	Male	LCI	OPD
Ibadan	41	female	LCI	OPD
Ibadan	19	female	LCI	OPD
Ibadan	19	Male	LCI	OPD
Ibadan	34	female	LCI	OPD
Ibadan	35	Male	LCI	OPD
Ibadan	56	Male	>1 CSI	OPD
Ibadan	7	Male	fever	OPD
Ibadan	38	Male	LCI	OPD
Ibadan	10	Male	poor feeding	OPD
Ibadan	46	female	fever	OPD
Ibadan	41	female	LCI	OPD
Ibadan	20	female	LCI	OPD
Ibadan	21	Male	LCI	OPD
Ibadan	21	Male	LCI	OPD
Ibadan	23	Male	LCI	OPD
Ibadan	9	Male	fever	OPD
Ibadan	14	female	LCI	OPD
Ibadan	30	female	LCI	OPD
Ibadan	28	female	>1 CSI	OPD
Ibadan	55	Male	LCI	OPD
Ibadan	29	female	LCI	OPD
Ibadan	35	Male	LCI	OPD
Ibadan	7	female	LCI	OPD
Ibadan	29	Male	LCI	OPD
Ibadan	42	female	>1 CSI	OPD
Ibadan	41	Male	>1 CSI	OPD

Ibadan	21	Male	LCI	OPD
Ibadan	41	Male	LCI	OPD
Ibadan	15	Male	LCI	OPD
Ibadan	18	Male	LCI	OPD
Ibadan	11	female	LCI	OPD
Ibadan	7	female	fever	OPD
Ibadan	48	female	fever	OPD
Ibadan	52	Male	fever	OPD
Ibadan	24	Male	>1 CSI	OPD
Ibadan	13	female	LCI	OPD
Ibadan	21	female	LCI	OPD
Ibadan	24	Male	fever	OPD
Ibadan	20	Male	fever	OPD
Ibadan	37	Male	>1 CSI	OPD
Ibadan	18	Male	LCI	OPD
Ibadan	53	Male	LCI	OPD
Ibadan	21	Male	LCI	OPD
Ibadan	17	Male	LCI	OPD
Ibadan	14	female	LCI	OPD
Ibadan	15	Male	LCI	OPD
Ibadan	42	female	LCI	OPD
Ibadan	35	Male	fever	OPD
Ibadan	31	female	movement on stimula	OPD
Ibadan	31	female	poor feeding	OPD
Ibadan	10	Male	poor feeding	OPD
Ibadan	21	Male	poor feeding	OPD
Ibadan	44	Male	>1 CSI	OPD
Ibadan	29	Male	LCI	OPD
Ibadan	41	female	movement on stimula	OPD
Ibadan	15	female	LCI	OPD
Ibadan	24	female	fever	OPD
Ibadan	58	Male	LCI	OPD
Ibadan	27	Male	LCI	OPD
Ibadan	21	female	fever	OPD
Ibadan	32	Male	poor feeding	OPD
Ibadan	46	female	>1 CSI	OPD
Ibadan	10	Male	fever	OPD
Ibadan	29	Male	>1 CSI	OPD
Ibadan	35	Male	fever	OPD
Ibadan	59	female	poor feeding	OPD
Ibadan	59	female	movement on stimula	OPD
Ibadan	31	female	poor feeding	OPD
Ibadan	22	female	>1 CSI	OPD
Ibadan	46	female	movement on stimula	OPD
Ibadan	23	female	fever	OPD
Ibadan	33	female	LCI	OPD
Ibadan	43	Male	fever	OPD
Ibadan	28	Male	LCI	OPD
Ibadan	59	Male	movement on stimula	OPD
Ibadan	9	Male	fever	OPD

Ibadan	37	Male	movement on stimula	OPD
Ibadan	44	female	>1 CSI	OPD
Ibadan	18	female	fever	OPD
Ibadan	16	female	>1 CSI	OPD
Ibadan	34	Male	fever	OPD
Ibadan	20	female	LCI	OPD
Ibadan	30	Male	>1 CSI	OPD
Ibadan	27	Male	LCI	OPD
Ibadan	34	Male	>1 CSI	OPD
Ibadan	42	Male	fever	OPD
Ibadan	11	Male	>1 CSI	OPD
Ibadan	36	Male	fever	OPD
Ibadan	21	female	fever	OPD
Ibadan	51	female	fever	OPD
Ibadan	49	Male	fever	OPD
Ibadan	10	Male	fever	OPD
Ibadan	42	female	fever	OPD
Ibadan	10	female	fever	OPD
Ibadan	21	Male	LCI	OPD
Ibadan	9	Male	fever	OPD
Ibadan	12	female	LCI	OPD
Ibadan	37	female	LCI	OPD
Ibadan	32	Male	fever	OPD
Ibadan	13	Male	fever	OPD
Ibadan	11	Male	fever	OPD
Ibadan	8	Male	fever	OPD
Ibadan	12	female	fever	OPD
Ibadan	38	Male	fever	OPD
Ibadan	9	Male	fever	OPD
Ibadan	10	female	fever	OPD
Ibadan	29	female	LCI	OPD
Ibadan	17	female	fever	OPD
Ibadan	18	Male	LCI	OPD
Ibadan	37	female	movement on stimula	OPD
Ibadan	23	Male	LCI	OPD
Ibadan	11	Male	LCI	OPD
Ibadan	8	Male	fever	OPD
Ibadan	48	female	fever	OPD
Ibadan	47	Male	LCI	OPD
Ibadan	16	female	LCI	OPD
Ibadan	11	female	fever	OPD
Ibadan	24	Male	fever	OPD
Ibadan	47	female	fever	OPD
Ibadan	52	Male	fever	OPD
Ibadan	48	female	>1 CSI	OPD
Ibadan	19	Male	fever	OPD
Ibadan	51	female	fever	OPD
Ibadan	8	Male	fever	OPD
Ibadan	7	female	fever	OPD
Ibadan	11	Male	fever	OPD

Ibadan	44	female	fever	OPD
Ibadan	50	Male	LCI	OPD
Ibadan	16	female	fever	OPD
Ibadan	7	Male	fever	OPD
Ibadan	40	female	fever	OPD
Ibadan	7	female	fever	OPD
Ibadan	8	female	fever	OPD
Ibadan	21	Male	LCI	OPD
Ibadan	13	Male	fever	OPD
Ibadan	19	Male	LCI	OPD
Ibadan	20	Male	fever	OPD
Ibadan	7	female	fever	OPD
Ibadan	8	female	fever	OPD
Ibadan	32	female	fever	OPD
Ibadan	22	Male	LCI	OPD
Ibadan	27	Male	fever	OPD
Ibadan	47	Male	fever	OPD
Ibadan	5	Male	fb0-6	OPD
Ibadan	2	Male	fb0-6	OPD
Ibadan	1	Male	fb0-6	OPD
Ibadan	1	Male	fb0-6	OPD
Ibadan	1	female	fb0-6	OPD
Ibadan	5	female	fb0-6	OPD
Ibadan	3	Male	fb0-6	OPD
Ibadan	3	female	fb0-6	OPD
Ibadan	3	Male	fb0-6	OPD
Ibadan	2	Male	fb0-6	OPD
Ibadan	2	female	fb0-6	OPD
Ibadan	6	Male	fb0-6	OPD
Ibadan	5	Male	fb0-6	OPD
Ibadan	4	female	fb0-6	OPD
Ibadan	6	Male	fb0-6	OPD
Ibadan	5	Male	fb0-6	OPD
Ibadan	1	female	fb0-6	OPD
Ibadan	0	Male	fb0-6	OPD
Ibadan	1	Male	fb0-6	OPD
Ibadan	3	female	fb0-6	OPD
Ibadan	3	Male	fb0-6	OPD
Ibadan	2	Male	fb0-6	OPD
Ibadan	6	Male	fb0-6	OPD
Ibadan	1	Male	fb0-6	OPD
Ibadan	3	Male	fb0-6	OPD
Ibadan	1	female	fb0-6	OPD
Ibadan	1	female	fb0-6	OPD
Ibadan	3	female	fb0-6	OPD
Ibadan	6	female	fb0-6	OPD
Ibadan	2	female	fb0-6	OPD
Ibadan	4	Male	fb0-6	OPD
Ibadan	6	female	fb0-6	OPD
Ibadan	3	female	fb0-6	OPD

Ibadan	1	Male	fb0-6	OPD
Ibadan	1	female	fb0-6	OPD
Ibadan	3	female	fb0-6	OPD
Ibadan	1	Male	fb0-6	OPD
Ibadan	1	Male	fb0-6	OPD
Ibadan	5	Male	fb0-6	OPD
Ibadan	4	Male	fb0-6	OPD
Ibadan	6	Male	fb0-6	OPD
Ibadan	6	female	fb0-6	OPD
Ibadan	3	female	fb0-6	OPD
Ibadan	3	female	fb0-6	OPD
Ibadan	3	Male	fb0-6	OPD
Ibadan	3	Male	fb0-6	OPD
Ibadan	6	Male	fb0-6	OPD
Ibadan	2	Male	fb0-6	OPD
Ibadan	2	Male	fb0-6	OPD
Ibadan	3	Male	fb0-6	OPD
Ibadan	4	Male	fb0-6	OPD
Ibadan	2	Male	fb0-6	OPD
Ibadan	3	Male	fb0-6	OPD
Ibadan	6	female	fb0-6	OPD
Ibadan	6	Male	fb0-6	OPD
Ibadan	2	female	fb0-6	OPD
Ibadan	3	Male	fb0-6	OPD
Ibadan	4	female	fb0-6	OPD
Ibadan	5	Male	fb0-6	OPD
Ibadan	1	Male	fb0-6	OPD
Ibadan	2	female	fb0-6	OPD
Ibadan	4	female	fb0-6	OPD
Ibadan	4	female	fb0-6	OPD
Ibadan	1	Male	fb0-6	OPD
Ibadan	5	female	fb0-6	OPD
Ibadan	1	Male	fb0-6	OPD
Ibadan	1	Male	fb0-6	OPD
Ibadan	2	Male	fb0-6	OPD
Ibadan	6	female	fb0-6	OPD
Ibadan	3	female	fb0-6	OPD
Ibadan	1	female	fb0-6	OPD
Ibadan	3	female	fb0-6	OPD
Ibadan	2	female	fb0-6	OPD
Ibadan	6	Male	fb0-6	OPD
Ibadan	3	female	fb0-6	OPD
Ibadan	1	Male	fb0-6	OPD
Ibadan	3	Male	fb0-6	OPD
Ibadan	1	Male	fb0-6	OPD
Ibadan	3	Male	fb0-6	OPD
Ibadan	6	female	fb0-6	OPD
Ibadan	6	Male	fb0-6	OPD
Ibadan	2	Male	fb0-6	OPD
Ibadan	3	Male	fb0-6	OPD

Ibadan	6 female	fb0-6	OPD
Ibadan	4 female	fb0-6	OPD
Ibadan	2 female	fb0-6	OPD
Ibadan	2 Male	fb0-6	OPD
Ibadan	2 female	fb0-6	OPD
Ibadan	5 Male	fb0-6	OPD
Ibadan	2 Male	fb0-6	OPD
Ibadan	5 Male	fb0-6	OPD
Ibadan	5 Male	fb0-6	OPD
Ibadan	2 Male	fb0-6	OPD
Ibadan	5 female	fb0-6	OPD
Ibadan	1 Male	fb0-6	OPD
Ibadan	6 Male	fb0-6	OPD
Ibadan	1 female	fb0-6	OPD
Ibadan	4 Male	fb0-6	OPD
Ibadan	6 Male	fb0-6	OPD
Ibadan	3 female	fb0-6	OPD
Ibadan	4 Male	fb0-6	OPD
Ibadan	1 female	fb0-6	OPD
Ibadan	4 Male	fb0-6	OPD
Ibadan	4 female	fb0-6	OPD
Ibadan	6 female	fb0-6	OPD
Ibadan	1 female	fb0-6	OPD
Ibadan	6 female	fb0-6	OPD
Ibadan	2 female	fb0-6	OPD
Ibadan	4 Male	fb0-6	OPD
Ibadan	1 Male	fb0-6	OPD
Ibadan	1 Male	fb0-6	OPD
Ibadan	3 female	fb0-6	OPD
Ibadan	1 Male	fb0-6	OPD
Ibadan	2 female	fb0-6	OPD
Ibadan	1 female	fb0-6	OPD
Ibadan	4 Male	fb0-6	OPD
Ibadan	1 female	fb0-6	OPD
Ibadan	6 Male	fb0-6	OPD
Ibadan	1 Male	fb0-6	OPD
Ibadan	1 female	fb0-6	OPD
Ibadan	3 Male	fb0-6	OPD
Ibadan	2 female	fb0-6	OPD
Ibadan	2 Male	fb0-6	OPD
Ibadan	5 female	fb0-6	OPD
Ibadan	2 female	fb0-6	OPD
Ibadan	2 female	fb0-6	OPD
Ibadan	5 Male	fb0-6	OPD
Ibadan	2 female	fb0-6	OPD
Ibadan	4 Male	fb0-6	OPD
Ibadan	2 female	fb0-6	OPD
Ibadan	1 Male	fb0-6	OPD
Ibadan	2 female	fb0-6	OPD
Ibadan	2 female	fb0-6	OPD

Ibadan	2	Male	fb0-6	OPD
Ibadan	5	Male	fb0-6	OPD
Ibadan	5	female	fb0-6	OPD
Ibadan	2	female	fb0-6	OPD
Ibadan	1	Male	fb0-6	OPD
Ibadan	3	female	fb0-6	OPD
Ibadan	2	Male	fb0-6	OPD
Ibadan	1	Male	fb0-6	OPD
Ibadan	6	female	fb0-6	OPD
Ibadan	2	female	fb0-6	OPD
Ibadan	3	female	fb0-6	OPD
Ibadan	1	Male	fb0-6	OPD
Ibadan	1	Male	fb0-6	OPD
Ibadan	5	female	fb0-6	OPD
Ibadan	6	female	fb0-6	OPD
Ibadan	5	Male	fb0-6	OPD
Ibadan	4	female	fb0-6	OPD
Ibadan	2	Male	fb0-6	OPD
Ibadan	1	Male	fb0-6	OPD
Ibadan	5	female	fb0-6	OPD
Ibadan	2	female	fb0-6	OPD
Ibadan	1	Male	fb0-6	OPD
Ibadan	3	Male	fb0-6	OPD
Ibadan	3	female	fb0-6	OPD
Ibadan	4	Male	fb0-6	OPD
Ibadan	4	female	fb0-6	OPD
Ibadan	4	Male	fb0-6	OPD
Ibadan	39	Male	FB 7-59	OPD
Ibadan	35	Male	FB 7-59	OPD
Ibadan	49	Male	FB 7-59	OPD
Ibadan	9	Male	FB 7-59	OPD
Ibadan	26	Male	FB 7-59	OPD
Ibadan	32	Male	FB 7-59	OPD
Ibadan	29	female	FB 7-59	OPD
Ibadan	8	female	FB 7-59	OPD
Ibadan	15	Male	FB 7-59	OPD
Ibadan	13	female	FB 7-59	OPD
Ibadan	7	female	FB 7-59	OPD
Ibadan	9	female	FB 7-59	OPD
Ibadan	9	Male	FB 7-59	OPD
Ibadan	59	Male	FB 7-59	OPD
Ibadan	16	Male	FB 7-59	OPD
Ibadan	17	female	FB 7-59	OPD
Ibadan	18	female	FB 7-59	OPD
Ibadan	9	female	FB 7-59	OPD
Ibadan	13	Male	FB 7-59	OPD
Ibadan	20	female	FB 7-59	OPD
Ibadan	28	Male	FB 7-59	OPD
Ibadan	21	female	FB 7-59	OPD
Ibadan	33	Male	FB 7-59	OPD

Ibadan	35	Male	FB 7-59	OPD
Ibadan	10	female	FB 7-59	OPD
Ibadan	26	Male	FB 7-59	OPD
Ibadan	14	female	FB 7-59	OPD
Ibadan	13	Male	FB 7-59	OPD
Ibadan	25	Male	FB 7-59	OPD
Ibadan	23	Male	FB 7-59	OPD
Ibadan	9	female	FB 7-59	OPD
Ibadan	22	female	FB 7-59	OPD
Ibadan	16	Male	FB 7-59	OPD
Ibadan	10	female	FB 7-59	OPD
Ibadan	45	female	FB 7-59	OPD
Ibadan	9	female	FB 7-59	OPD
Ibadan	8	Male	FB 7-59	OPD
Ibadan	22	female	FB 7-59	OPD
Ibadan	11	female	FB 7-59	OPD
Ibadan	16	female	FB 7-59	OPD
Ibadan	16	Male	FB 7-59	OPD
Ibadan	12	female	FB 7-59	OPD
Ibadan	25	Male	FB 7-59	OPD
Ibadan	40	Male	FB 7-59	OPD
Ibadan	36	female	FB 7-59	OPD
Ibadan	47	Male	FB 7-59	OPD
Ibadan	15	Male	FB 7-59	OPD
Ibadan	22	Male	FB 7-59	OPD
Ibadan	7	female	FB 7-59	OPD
Ibadan	30	Male	FB 7-59	OPD
Ibadan	21	female	FB 7-59	OPD
Ibadan	9	Male	FB 7-59	OPD
Ibadan	45	female	FB 7-59	OPD
Ibadan	39	female	FB 7-59	OPD
Ibadan	30	Male	FB 7-59	OPD
Ibadan	22	Male	FB 7-59	OPD
Ibadan	21	Male	FB 7-59	OPD
Ibadan	35	female	FB 7-59	OPD
Ibadan	21	female	FB 7-59	OPD
Ibadan	28	female	FB 7-59	OPD
Ibadan	46	Male	FB 7-59	OPD
Ibadan	31	female	FB 7-59	OPD
Ibadan	13	Male	FB 7-59	OPD
Ibadan	39	female	FB 7-59	OPD
Ibadan	15	female	FB 7-59	OPD
Ibadan	27	Male	FB 7-59	OPD
Ibadan	19	Male	FB 7-59	OPD
Ibadan	14	Male	FB 7-59	OPD
Ibadan	29	female	FB 7-59	OPD
Ibadan	22	Male	FB 7-59	OPD
Ibadan	15	Male	FB 7-59	OPD
Ibadan	34	Male	FB 7-59	OPD
Ibadan	15	female	FB 7-59	OPD

Ibadan	10	Male	FB 7-59	OPD
Ibadan	15	female	FB 7-59	OPD
Ibadan	19	Male	FB 7-59	OPD
Ibadan	14	female	FB 7-59	OPD
Ibadan	29	female	FB 7-59	OPD
Ibadan	9	Male	FB 7-59	OPD
Ibadan	38	female	FB 7-59	OPD
Ibadan	12	female	FB 7-59	OPD
Ibadan	18	Male	FB 7-59	OPD
Ibadan	28	female	FB 7-59	OPD
Ibadan	16	Male	FB 7-59	OPD
Ibadan	9	female	FB 7-59	OPD
Ibadan	9	Male	FB 7-59	OPD
Ibadan	22	female	FB 7-59	OPD
Ibadan	36	female	FB 7-59	OPD
Ibadan	16	female	FB 7-59	OPD
Ibadan	15	Male	FB 7-59	OPD
Ibadan	25	female	FB 7-59	OPD
Ibadan	8	Male	FB 7-59	OPD
Ibadan	17	female	FB 7-59	OPD
Ibadan	15	female	FB 7-59	OPD
Ibadan	42	Male	FB 7-59	OPD
Ibadan	11	Male	FB 7-59	OPD
Ibadan	7	Male	FB 7-59	OPD
Ibadan	8	Male	FB 7-59	OPD
Ibadan	20	female	FB 7-59	OPD
Ibadan	8	Male	FB 7-59	OPD
Ibadan	8	female	FB 7-59	OPD
Ibadan	39	female	FB 7-59	OPD
Ibadan	8	Male	FB 7-59	OPD
Ibadan	34	Male	FB 7-59	OPD
Ibadan	35	Male	FB 7-59	OPD
Ibadan	9	female	FB 7-59	OPD
Ibadan	13	female	FB 7-59	OPD
Ibadan	16	Male	FB 7-59	OPD
Ibadan	31	female	FB 7-59	OPD
Ibadan	19	Male	FB 7-59	OPD
Ibadan	16	female	FB 7-59	OPD
Ibadan	9	Male	FB 7-59	OPD
Ibadan	28	Male	FB 7-59	OPD
Ibadan	9	female	FB 7-59	OPD
Ibadan	52	female	FB 7-59	OPD
Ibadan	25	Male	FB 7-59	OPD
Ibadan	23	female	FB 7-59	OPD
Ibadan	22	Male	FB 7-59	OPD
Ibadan	47	Male	FB 7-59	OPD
Ibadan	14	Male	FB 7-59	OPD
Ibadan	7	Male	FB 7-59	OPD
Ibadan	14	Male	FB 7-59	OPD
Ibadan	15	Male	FB 7-59	OPD

Ibadan	16	Male	FB 7-59	OPD
Ibadan	11	Male	FB 7-59	OPD
Ibadan	7	female	FB 7-59	OPD
Ibadan	16	female	FB 7-59	OPD
Ibadan	7	Male	FB 7-59	OPD
Ibadan	35	female	FB 7-59	OPD
Ibadan	13	Male	FB 7-59	OPD
Ibadan	11	Male	FB 7-59	OPD
Ibadan	7	female	FB 7-59	OPD
Ibadan	29	female	FB 7-59	OPD
Ibadan	27	Male	FB 7-59	OPD
Ibadan	35	Male	FB 7-59	OPD
Ibadan	16	female	FB 7-59	OPD
Ibadan	20	female	FB 7-59	OPD
Ibadan	9	female	FB 7-59	OPD
Ibadan	13	female	FB 7-59	OPD
Ibadan	8	female	FB 7-59	OPD
Ibadan	30	female	FB 7-59	OPD
Ibadan	8	female	FB 7-59	OPD
Ibadan	9	Male	FB 7-59	OPD
Ibadan	8	female	FB 7-59	OPD
Ibadan	9	Male	FB 7-59	OPD
Ibadan	20	Male	FB 7-59	OPD
Ibadan	28	Male	FB 7-59	OPD
Ibadan	19	Male	FB 7-59	OPD
Ibadan	14	Male	FB 7-59	OPD
Ibadan	44	Male	FB 7-59	OPD
Ibadan	3	Male	fb0-6	OPD
Ibadan	25	Male	FB 7-59	OPD
Ibadan	19	female	FB 7-59	OPD
Ibadan	19	female	FB 7-59	OPD
Ibadan	24	Male	FB 7-59	OPD
Ibadan	30	Male	FB 7-59	OPD
Ibadan	19	Male	FB 7-59	OPD
Ibadan	7	Male	FB 7-59	OPD
Ibadan	16	female	FB 7-59	OPD
Ibadan	21	female	FB 7-59	OPD
Ile Ife	5	female	LCI	OPD
Ile Ife	6	female	LCI	OPD
Ile Ife	5	Male	LCI	OPD
Ile Ife	6	female	fever	OPD
Ile Ife	3	Male	LCI	OPD
Ile Ife	5	female	LCI	OPD
Ile Ife	4	female	LCI	OPD
Ile Ife	2	female	LCI	OPD
Ile Ife	6	female	LCI	OPD
Ile Ife	5	female	LCI	OPD
Ile Ife	3	female	LCI	OPD
Ile Ife	5	Male	>1 CSI	OPD
Ile Ife	2	Male	fever	OPD

Ile Ife	2	Male	fever	OPD
Ile Ife	4	female	LCI	OPD
Ile Ife	1	Male	LCI	OPD
Ile Ife	3	female	>1 CSI	OPD
Ile Ife	6	Male	LCI	OPD
Ile Ife	3	Male	fever	OPD
Ile Ife	4	female	fever	OPD
Ile Ife	6	Male	LCI	OPD
Ile Ife	3	Male	>1 CSI	OPD
Ile Ife	3	Male	>1 CSI	OPD
Ile Ife	3	female	LCI	OPD
Ile Ife	2	Male	LCI	OPD
Ile Ife	6	female	fever	OPD
Ile Ife	5	female	LCI	OPD
Ile Ife	1	Male	fever	OPD
Ile Ife	4	Male	>1 CSI	OPD
Ile Ife	6	female	LCI	OPD
Ile Ife	4	Male	LCI	OPD
Ile Ife	3	female	LCI	OPD
Ile Ife	6	female	fever	OPD
Ile Ife	2	female	fever	OPD
Ile Ife	4	female	fever	OPD
Ile Ife	3	female	LCI	OPD
Ile Ife	3	Male	LCI	OPD
Ile Ife	2	female	>1 CSI	OPD
Ile Ife	2	Male	>1 CSI	OPD
Ile Ife	1	female	fever	OPD
Ile Ife	5	female	LCI	OPD
Ile Ife	5	Male	fever	OPD
Ile Ife	5	Male	LCI	OPD
Ile Ife	2	Male	fever	OPD
Ile Ife	5	Male	LCI	OPD
Ile Ife	2	female	fever	OPD
Ile Ife	6	female	LCI	OPD
Ile Ife	4	female	fever	OPD
Ile Ife	2	Male	fever	OPD
Ile Ife	3	female	LCI	OPD
Ile Ife	1	female	fever	OPD
Ile Ife	4	Male	LCI	OPD
Ile Ife	6	female	LCI	OPD
Ile Ife	6	female	>1 CSI	OPD
Ile Ife	3	female	LCI	OPD
Ile Ife	6	female	LCI	OPD
Ile Ife	1	Male	fever	OPD
Ile Ife	3	Male	fever	OPD
Ile Ife	1	female	fever	OPD
Ile Ife	6	female	fever	OPD
Ile Ife	4	Male	fever	OPD
Ile Ife	4	female	fever	OPD
Ile Ife	2	Male	fever	OPD

Ile Ife	4	Male	LCI	OPD
Ile Ife	6	Male	fever	OPD
Ile Ife	4	Male	fever	OPD
Ile Ife	5	female	LCI	OPD
Ile Ife	4	female	fever	OPD
Ile Ife	1	Male	fever	OPD
Ile Ife	2	female	LCI	OPD
Ile Ife	3	female	LCI	OPD
Ile Ife	2	Male	>1 CSI	OPD
Ile Ife	3	Male	fever	OPD
Ile Ife	6	female	LCI	OPD
Ile Ife	2	female	fever	OPD
Ile Ife	3	female	LCI	OPD
Ile Ife	1	Male	fever	OPD
Ile Ife	2	Male	fever	OPD
Ile Ife	6	female	LCI	OPD
Ile Ife	2	Male	>1 CSI	OPD
Ile Ife	3	female	LCI	OPD
Ile Ife	3	Male	LCI	OPD
Ile Ife	6	Male	fever	OPD
Ile Ife	6	female	LCI	OPD
Ile Ife	1	Male	fever	OPD
Ile Ife	1	Male	fever	OPD
Ile Ife	2	Male	LCI	OPD
Ile Ife	6	female	LCI	OPD
Ile Ife	5	female	fever	OPD
Ile Ife	2	female	fever	OPD
Ile Ife	2	female	fever	OPD
Ile Ife	2	Male	>1 CSI	OPD
Ile Ife	5	Male	LCI	OPD
Ile Ife	2	Male	fever	OPD
Ile Ife	1	Male	LCI	OPD
Ile Ife	5	Male	LCI	OPD
Ile Ife	4	Male	fever	OPD
Ile Ife	2	female	LCI	OPD
Ile Ife	5	Male	fever	OPD
Ile Ife	5	Male	LCI	OPD
Ile Ife	2	Male	LCI	OPD
Ile Ife	2	Male	fever	OPD
Ile Ife	2	Male	fever	OPD
Ile Ife	2	female	LCI	OPD
Ile Ife	6	female	LCI	OPD
Ile Ife	5	female	LCI	OPD
Ile Ife	6	female	fever	OPD
Ile Ife	1	Male	LCI	OPD
Ile Ife	3	Male	LCI	OPD
Ile Ife	2	Male	fever	OPD
Ile Ife	5	Male	LCI	OPD
Ile Ife	5	female	Hypo	OPD
Ile Ife	1	female	LCI	OPD

Ile Ife	5	female	fever	OPD
Ile Ife	6	Male	LCI	OPD
Ile Ife	2	Male	fever	OPD
Ile Ife	5	Male	LCI	OPD
Ile Ife	3	female	fever	OPD
Ile Ife	2	female	LCI	OPD
Ile Ife	4	Male	LCI	OPD
Ile Ife	1	Male	LCI	OPD
Ile Ife	1	female	LCI	OPD
Ile Ife	2	Male	fever	OPD
Ile Ife	5	female	LCI	OPD
Ile Ife	1	female	Hypo	OPD
Ile Ife	3	female	LCI	OPD
Ile Ife	1	Male	LCI	OPD
Ile Ife	5	Male	LCI	OPD
Ile Ife	3	Male	LCI	OPD
Ile Ife	2	Male	LCI	OPD
Ile Ife	4	female	LCI	OPD
Ile Ife	5	Male	LCI	OPD
Ile Ife	4	female	LCI	OPD
Ile Ife	2	female	LCI	OPD
Ile Ife	2	Male	LCI	OPD
Ile Ife	2	Male	LCI	OPD
Ile Ife	1	Male	>1 CSI	OPD
Ile Ife	6	Male	LCI	OPD
Ile Ife	3	female	fever	OPD
Ile Ife	3	female	LCI	OPD
Ile Ife	1	female	Hypo	OPD
Ile Ife	1	Male	Hypo	OPD
Ile Ife	1	female	Hypo	OPD
Ile Ife	3	Male	LCI	OPD
Ile Ife	1	Male	fever	OPD
Ile Ife	5	Male	fever	OPD
Ile Ife	2	female	fever	OPD
Ile Ife	2	female	fever	OPD
Ile Ife	2	female	fever	OPD
Ile Ife	1	Male	Hypo	OPD
Ile Ife	2	female	fever	OPD
Ile Ife	6	Male	fever	OPD
Ile Ife	2	female	fever	OPD
Ile Ife	4	Male	fever	OPD
Ile Ife	2	female	fever	OPD
Ile Ife	1	Male	fever	OPD
Ile Ife	6	female	LCI	OPD
Ile Ife	3	Male	LCI	OPD
Ile Ife	3	Male	fever	OPD
Ile Ife	2	Male	fever	OPD
Ile Ife	6	female	LCI	OPD
Ile Ife	2	Male	LCI	OPD
Ile Ife	2	female	fever	OPD

Ile Ife	3	Male	fever	OPD
Ile Ife	2	female	fever	OPD
Ile Ife	2	Male	fever	OPD
Ile Ife	3	Male	>1 CSI	OPD
Ile Ife	3	Male	fever	OPD
Ile Ife	1	female	fever	OPD
Ile Ife	2	female	fever	OPD
Ile Ife	1	Male	fever	OPD
Ile Ife	1	Male	fever	OPD
Ile Ife	4	Male	fever	OPD
Ile Ife	2	Male	fever	OPD
Ile Ife	2	female	fever	OPD
Ile Ife	3	Male	fever	OPD
Ile Ife	4	Male	fever	OPD
Ile Ife	5	female	fever	OPD
Ile Ife	2	female	fever	OPD
Ile Ife	2	female	fever	OPD
Ile Ife	5	Male	LCI	OPD
Ile Ife	3	female	LCI	OPD
Ile Ife	6	Male	LCI	OPD
Ile Ife	4	female	LCI	OPD
Ile Ife	5	female	>1 CSI	OPD
Ile Ife	1	Male	fever	OPD
Ile Ife	5	female	fever	OPD
Ile Ife	3	Male	LCI	OPD
Ile Ife	1	Male	fever	OPD
Ile Ife	4	Male	LCI	OPD
Ile Ife	5	female	fever	OPD
Ile Ife	3	female	fever	OPD
Ile Ife	4	female	poor feeding	OPD
Ile Ife	1	Male	fever	OPD
Ile Ife	2	Male	LCI	OPD
Ile Ife	1	female	fever	OPD
Ile Ife	4	Male	LCI	OPD
Ile Ife	1	female	LCI	OPD
Ile Ife	1	female	LCI	OPD
Ile Ife	2	Male	>1 CSI	OPD
Ile Ife	5	Male	>1 CSI	OPD
Ile Ife	2	female	LCI	OPD
Ile Ife	2	female	LCI	OPD
Ile Ife	1	female	fever	OPD
Ile Ife	6	Male	LCI	OPD
Ile Ife	1	female	Hypo	OPD
Ile Ife	1	female	LCI	OPD
Ile Ife	3	female	fever	OPD
Ile Ife	2	female	fever	OPD
Ile Ife	1	female	fever	OPD
Ile Ife	3	Male	LCI	OPD
Ile Ife	2	female	>1 CSI	OPD
Ile Ife	1	Male	LCI	OPD

Ile Ife	4	female	LCI	OPD
Ile Ife	4	Male	LCI	OPD
Ile Ife	4	female	LCI	OPD
Ile Ife	1	female	LCI	OPD
Ile Ife	2	female	fever	OPD
Ile Ife	2	Male	LCI	OPD
Ile Ife	6	Male	LCI	OPD
Ile Ife	2	Male	LCI	OPD
Ile Ife	5	female	fever	OPD
Ile Ife	1	Male	>1 CSI	OPD
Ile Ife	1	female	>1 CSI	OPD
Ile Ife	2	female	LCI	OPD
Ile Ife	4	Male	>1 CSI	OPD
Ile Ife	3	Male	LCI	OPD
Ile Ife	1	Male	LCI	OPD
Ile Ife	1	Male	fever	OPD
Ile Ife	2	female	LCI	OPD
Ile Ife	1	Male	LCI	OPD
Ile Ife	1	Male	LCI	OPD
Ile Ife	1	female	LCI	OPD
Ile Ife	1	female	fever	OPD
Ile Ife	4	female	LCI	OPD
Ile Ife	1	Male	LCI	OPD
Ile Ife	2	Male	LCI	OPD
Ile Ife	1	female	fever	OPD
Ile Ife	5	Male	LCI	OPD
Ile Ife	4	female	LCI	OPD
Ile Ife	3	female	fever	OPD
Ile Ife	2	Male	fever	OPD
Ile Ife	4	Male	LCI	OPD
Ile Ife	2	Male	LCI	OPD
Ile Ife	1	female	LCI	OPD
Ile Ife	5	female	LCI	OPD
Ile Ife	1	female	fever	OPD
Ile Ife	1	female	fever	OPD
Ile Ife	5	female	LCI	OPD
Ile Ife	2	Male	fever	OPD
Ile Ife	3	female	fever	OPD
Ile Ife	2	female	fever	OPD
Ile Ife	1	Male	fever	OPD
Ile Ife	2	female	fever	OPD
Ile Ife	5	female	LCI	OPD
Ile Ife	3	female	LCI	OPD
Ile Ife	5	female	LCI	OPD
Ile Ife	4	female	fever	OPD
Ile Ife	6	female	fever	OPD
Ile Ife	2	Male	fever	OPD
Ile Ife	5	Male	>1 CSI	OPD
Ile Ife	1	female	fever	OPD
Ile Ife	2	Male	LCI	OPD

Ile Ife	3	Male	fever	OPD
Ile Ife	34	Male	LCI	OPD
Ile Ife	42	Male	LCI	OPD
Ile Ife	7	Male	LCI	OPD
Ile Ife	29	female	LCI	OPD
Ile Ife	14	female	LCI	OPD
Ile Ife	44	female	fever	OPD
Ile Ife	35	female	LCI	OPD
Ile Ife	24	Male	fever	OPD
Ile Ife	11	female	LCI	OPD
Ile Ife	41	female	LCI	OPD
Ile Ife	28	Male	fever	OPD
Ile Ife	11	female	fever	OPD
Ile Ife	7	female	fever	OPD
Ile Ife	12	female	LCI	OPD
Ile Ife	11	female	LCI	OPD
Ile Ife	20	female	LCI	OPD
Ile Ife	26	Male	LCI	OPD
Ile Ife	42	Male	LCI	OPD
Ile Ife	21	Male	fever	OPD
Ile Ife	46	Male	LCI	OPD
Ile Ife	23	female	LCI	OPD
Ile Ife	8	Male	LCI	OPD
Ile Ife	40	Male	fever	OPD
Ile Ife	25	female	LCI	OPD
Ile Ife	19	female	fever	OPD
Ile Ife	14	female	LCI	OPD
Ile Ife	22	female	LCI	OPD
Ile Ife	14	Male	LCI	OPD
Ile Ife	19	female	LCI	OPD
Ile Ife	9	Male	LCI	OPD
Ile Ife	46	Male	fever	OPD
Ile Ife	14	Male	LCI	OPD
Ile Ife	20	female	LCI	OPD
Ile Ife	14	female	LCI	OPD
Ile Ife	19	Male	fever	OPD
Ile Ife	35	Male	LCI	OPD
Ile Ife	17	Male	LCI	OPD
Ile Ife	26	Male	LCI	OPD
Ile Ife	8	female	fever	OPD
Ile Ife	30	female	poor feeding	OPD
Ile Ife	9	female	fever	OPD
Ile Ife	14	Male	LCI	OPD
Ile Ife	38	Male	LCI	OPD
Ile Ife	23	Male	LCI	OPD
Ile Ife	24	Male	fever	OPD
Ile Ife	13	Male	>1 CSI	OPD
Ile Ife	50	Male	LCI	OPD
Ile Ife	14	Male	LCI	OPD
Ile Ife	14	Male	LCI	OPD

Ile Ife	43	Male	LCI	OPD
Ile Ife	28	female	LCI	OPD
Ile Ife	7	Male	LCI	OPD
Ile Ife	21	Male	LCI	OPD
Ile Ife	11	female	LCI	OPD
Ile Ife	35	female	LCI	OPD
Ile Ife	19	female	LCI	OPD
Ile Ife	9	Male	LCI	OPD
Ile Ife	15	female	LCI	OPD
Ile Ife	26	female	LCI	OPD
Ile Ife	22	Male	LCI	OPD
Ile Ife	27	female	LCI	OPD
Ile Ife	15	Male	LCI	OPD
Ile Ife	31	Male	LCI	OPD
Ile Ife	12	female	LCI	OPD
Ile Ife	51	female	LCI	OPD
Ile Ife	22	Male	LCI	OPD
Ile Ife	23	female	LCI	OPD
Ile Ife	8	Male	LCI	OPD
Ile Ife	26	Male	fever	OPD
Ile Ife	14	female	fever	OPD
Ile Ife	21	female	poor feeding	OPD
Ile Ife	42	female	LCI	OPD
Ile Ife	8	female	LCI	OPD
Ile Ife	23	Male	LCI	OPD
Ile Ife	34	Male	LCI	OPD
Ile Ife	9	Male	>1 CSI	OPD
Ile Ife	8	Male	LCI	OPD
Ile Ife	14	female	LCI	OPD
Ile Ife	26	female	LCI	OPD
Ile Ife	20	female	LCI	OPD
Ile Ife	10	Male	LCI	OPD
Ile Ife	30	Male	LCI	OPD
Ile Ife	27	female	fever	OPD
Ile Ife	8	Male	LCI	OPD
Ile Ife	21	female	LCI	OPD
Ile Ife	19	Male	LCI	OPD
Ile Ife	22	Male	LCI	OPD
Ile Ife	21	Male	LCI	OPD
Ile Ife	33	Male	LCI	OPD
Ile Ife	8	Male	LCI	OPD
Ile Ife	9	female	LCI	OPD
Ile Ife	24	Male	LCI	OPD
Ile Ife	7	Male	LCI	OPD
Ile Ife	11	Male	LCI	OPD
Ile Ife	16	Male	LCI	OPD
Ile Ife	45	Male	fever	OPD
Ile Ife	18	female	fever	OPD
Ile Ife	12	Male	LCI	OPD
Ile Ife	13	female	LCI	OPD

Ile Ife	12	female	LCI	OPD
Ile Ife	54	female	fever	OPD
Ile Ife	20	female	fever	OPD
Ile Ife	52	female	fever	OPD
Ile Ife	35	Male	fever	OPD
Ile Ife	45	female	LCI	OPD
Ile Ife	30	female	LCI	OPD
Ile Ife	32	female	LCI	OPD
Ile Ife	47	Male	fever	OPD
Ile Ife	11	Male	fever	OPD
Ile Ife	21	female	fever	OPD
Ile Ife	13	Male	LCI	OPD
Ile Ife	47	female	fever	OPD
Ile Ife	13	female	fever	OPD
Ile Ife	30	Male	fever	OPD
Ile Ife	23	female	fever	OPD
Ile Ife	7	Male	fever	OPD
Ile Ife	8	female	LCI	OPD
Ile Ife	23	female	fever	OPD
Ile Ife	7	female	fever	OPD
Ile Ife	8	Male	fever	OPD
Ile Ife	55	Male	LCI	OPD
Ile Ife	15	female	fever	OPD
Ile Ife	14	Male	fever	OPD
Ile Ife	7	Male	fever	OPD
Ile Ife	9	Male	fever	OPD
Ile Ife	19	Male	fever	OPD
Ile Ife	24	Male	LCI	OPD
Ile Ife	8	female	fever	OPD
Ile Ife	11	Male	LCI	OPD
Ile Ife	34	Male	fever	OPD
Ile Ife	38	Male	fever	OPD
Ile Ife	18	Male	fever	OPD
Ile Ife	20	Male	LCI	OPD
Ile Ife	26	female	fever	OPD
Ile Ife	14	Male	fever	OPD
Ile Ife	24	Male	LCI	OPD
Ile Ife	16	Male	LCI	OPD
Ile Ife	20	female	LCI	OPD
Ile Ife	11	female	LCI	OPD
Ile Ife	7	female	fever	OPD
Ile Ife	25	Male	LCI	OPD
Ile Ife	16	Male	fever	OPD
Ile Ife	22	female	fever	OPD
Ile Ife	21	female	LCI	OPD
Ile Ife	37	Male	LCI	OPD
Ile Ife	10	female	fever	OPD
Ile Ife	7	female	fever	OPD
Ile Ife	17	Male	fever	OPD
Ile Ife	14	Male	LCI	OPD

Ile Ife	23	female	LCI	OPD
Ile Ife	8	female	LCI	OPD
Ile Ife	51	female	LCI	OPD
Ile Ife	33	Male	LCI	OPD
Ile Ife	17	Male	LCI	OPD
Ile Ife	11	Male	LCI	OPD
Ile Ife	9	Male	LCI	OPD
Ile Ife	39	female	LCI	OPD
Ile Ife	14	female	LCI	OPD
Ile Ife	16	female	LCI	OPD
Ile Ife	30	female	>1 CSI	OPD
Ile Ife	28	Male	LCI	OPD
Ile Ife	34	Male	>1 CSI	OPD
Ile Ife	7	female	fever	OPD
Ile Ife	10	Male	fever	OPD
Ile Ife	37	female	LCI	OPD
Ile Ife	7	female	LCI	OPD
Ile Ife	35	female	fever	OPD
Ile Ife	20	Male	>1 CSI	OPD
Ile Ife	21	Male	LCI	OPD
Ile Ife	9	Male	LCI	OPD
Ile Ife	25	Male	fever	OPD
Ile Ife	28	Male	LCI	OPD
Ile Ife	28	female	fever	OPD
Ile Ife	10	Male	>1 CSI	OPD
Ile Ife	25	female	LCI	OPD
Ile Ife	43	female	>1 CSI	OPD
Ile Ife	32	female	fever	OPD
Ile Ife	27	female	fever	OPD
Ile Ife	7	Male	LCI	OPD
Ile Ife	23	Male	LCI	OPD
Ile Ife	38	female	LCI	OPD
Ile Ife	7	Male	LCI	OPD
Ile Ife	10	Male	LCI	OPD
Ile Ife	22	female	LCI	OPD
Ile Ife	7	Male	fever	OPD
Ile Ife	14	female	LCI	OPD
Ile Ife	46	female	LCI	OPD
Ile Ife	15	Male	LCI	OPD
Ile Ife	19	Male	LCI	OPD
Ile Ife	8	Male	LCI	OPD
Ile Ife	32	female	LCI	OPD
Ile Ife	9	female	>1 CSI	OPD
Ile Ife	30	female	>1 CSI	OPD
Ile Ife	44	Male	LCI	OPD
Ile Ife	42	female	LCI	OPD
Ile Ife	29	female	LCI	OPD
Ile Ife	24	Male	LCI	OPD
Ile Ife	8	Male	LCI	OPD
Ile Ife	35	Male	LCI	OPD

Ile Ife	23	female	LCI	OPD
Ile Ife	23	female	LCI	OPD
Ile Ife	37	female	fever	OPD
Ile Ife	23	Male	LCI	OPD
Ile Ife	16	Male	LCI	OPD
Ile Ife	34	female	LCI	OPD
Ile Ife	17	Male	LCI	OPD
Ile Ife	13	Male	LCI	OPD
Ile Ife	32	Male	LCI	OPD
Ile Ife	8	female	LCI	OPD
Ile Ife	14	female	LCI	OPD
Ile Ife	12	female	LCI	OPD
Ile Ife	27	Male	LCI	OPD
Ile Ife	13	Male	LCI	OPD
Ile Ife	29	female	LCI	OPD
Ile Ife	33	Male	LCI	OPD
Ile Ife	15	female	fever	OPD
Ile Ife	28	Male	fever	OPD
Ile Ife	11	Male	LCI	OPD
Ile Ife	25	Male	LCI	OPD
Ile Ife	14	Male	LCI	OPD
Ile Ife	9	Male	LCI	OPD
Ile Ife	16	Male	LCI	OPD
Ile Ife	21	Male	LCI	OPD
Ile Ife	8	female	LCI	OPD
Ile Ife	35	Male	LCI	OPD
Ile Ife	17	Male	fever	OPD
Ile Ife	18	female	LCI	OPD
Ile Ife	22	Male	LCI	OPD
Ile Ife	22	female	LCI	OPD
Ile Ife	17	female	LCI	OPD
Ile Ife	12	Male	fever	OPD
Ile Ife	19	Male	LCI	OPD
Ile Ife	35	female	LCI	OPD
Ile Ife	26	Male	LCI	OPD
Ile Ife	19	female	LCI	OPD
Ile Ife	23	Male	fever	OPD
Ile Ife	14	female	LCI	OPD
Ile Ife	51	Male	LCI	OPD
Ile Ife	7	Male	fever	OPD
Ile Ife	11	Male	fever	OPD
Ile Ife	18	female	fever	OPD
Ile Ife	9	Male	fever	OPD
Ile Ife	18	female	fever	OPD
Ile Ife	48	Male	LCI	OPD
Ile Ife	17	Male	LCI	OPD
Ile Ife	10	female	fever	OPD
Ile Ife	16	female	fever	OPD
Ile Ife	15	Male	LCI	OPD
Ile Ife	14	Male	LCI	OPD

Ile Ife	9	female	LCI	OPD
Ile Ife	18	female	LCI	OPD
Ile Ife	56	Male	fever	OPD
Ile Ife	8	Male	fever	OPD
Ile Ife	17	female	LCI	OPD
Ile Ife	40	Male	LCI	OPD
Ile Ife	58	female	LCI	OPD
Ile Ife	14	female	poor feeding	OPD
Ile Ife	10	female	LCI	OPD
Ile Ife	12	Male	LCI	OPD
Ile Ife	34	Male	LCI	OPD
Ile Ife	19	female	LCI	OPD
Ile Ife	9	female	LCI	OPD
Ile Ife	24	Male	LCI	OPD
Ile Ife	9	Male	LCI	OPD
Ile Ife	11	female	fever	OPD
Ile Ife	11	female	LCI	OPD
Ile Ife	21	female	LCI	OPD
Ile Ife	9	Male	LCI	OPD
Ile Ife	17	female	LCI	OPD
Ile Ife	21	Male	LCI	OPD
Ile Ife	13	Male	LCI	OPD
Ile Ife	19	Male	LCI	OPD
Ile Ife	12	female	fever	OPD
Ile Ife	17	Male	fever	OPD
Ile Ife	15	female	LCI	OPD
Ile Ife	11	Male	LCI	OPD
Ile Ife	8	female	fever	OPD
Ile Ife	12	Male	LCI	OPD
Ile Ife	25	female	LCI	OPD
Ile Ife	19	female	LCI	OPD
Ile Ife	19	female	LCI	OPD
Ile Ife	20	Male	LCI	OPD
Ile Ife	35	Male	LCI	OPD
Ile Ife	13	female	LCI	OPD
Ile Ife	18	Male	LCI	OPD
Ile Ife	38	female	LCI	OPD
Ile Ife	26	Male	LCI	OPD
Ile Ife	15	female	LCI	OPD
Ile Ife	31	Male	>1 CSI	OPD
Ile Ife	34	female	LCI	OPD
Ile Ife	35	female	LCI	OPD
Ile Ife	35	Male	LCI	OPD
Ile Ife	10	female	LCI	OPD
Ile Ife	38	female	LCI	OPD
Ile Ife	42	female	LCI	OPD
Ile Ife	14	female	LCI	OPD
Ile Ife	27	female	LCI	OPD
Ile Ife	13	female	>1 CSI	OPD
Ile Ife	42	female	LCI	OPD

Ile Ife	46	female	LCI	OPD
Ile Ife	36	Male	>1 CSI	OPD
Ile Ife	45	female	fever	OPD
Ile Ife	8	female	LCI	OPD
Ile Ife	7	Male	LCI	OPD
Ile Ife	39	Male	LCI	OPD
Ile Ife	8	female	LCI	OPD
Ile Ife	51	Male	LCI	OPD
Ile Ife	46	Male	fever	OPD
Ile Ife	35	Male	LCI	OPD
Ile Ife	8	female	LCI	OPD
Ile Ife	17	Male	LCI	OPD
Ile Ife	19	Male	LCI	OPD
Ile Ife	14	Male	LCI	OPD
Ile Ife	11	female	LCI	OPD
Ile Ife	9	Male	>1 CSI	OPD
Ile Ife	30	Male	LCI	OPD
Ile Ife	22	female	LCI	OPD
Ile Ife	26	Male	LCI	OPD
Ile Ife	12	female	fever	OPD
Ile Ife	14	female	LCI	OPD
Ile Ife	19	female	LCI	OPD
Ile Ife	10	Male	fever	OPD
Ile Ife	29	Male	LCI	OPD
Ile Ife	57	Male	LCI	OPD
Ile Ife	27	female	LCI	OPD
Ile Ife	13	female	LCI	OPD
Ile Ife	30	Male	LCI	OPD
Ile Ife	8	Male	LCI	OPD
Ile Ife	8	female	LCI	OPD
Ile Ife	35	Male	LCI	OPD
Ile Ife	7	female	LCI	OPD
Ile Ife	38	female	LCI	OPD
Ile Ife	24	Male	LCI	OPD
Ile Ife	25	Male	LCI	OPD
Ile Ife	12	Male	fever	OPD
Ile Ife	10	female	LCI	OPD
Ile Ife	27	Male	LCI	OPD
Ile Ife	35	Male	LCI	OPD
Ile Ife	29	Male	LCI	OPD
Ile Ife	33	Male	LCI	OPD
Ile Ife	22	Male	LCI	OPD
Ile Ife	42	Male	LCI	OPD
Ile Ife	22	Male	fever	OPD
Ile Ife	19	female	LCI	OPD
Ile Ife	48	Male	LCI	OPD
Ile Ife	28	Male	LCI	OPD
Ile Ife	9	female	LCI	OPD
Ile Ife	21	Male	LCI	OPD
Ile Ife	7	female	fever	OPD

Ile Ife	8	Male	LCI	OPD
Ile Ife	17	female	LCI	OPD
Ile Ife	32	female	LCI	OPD
Ile Ife	12	Male	LCI	OPD
Ile Ife	16	Male	LCI	OPD
Ile Ife	13	female	LCI	OPD
Ile Ife	20	female	LCI	OPD
Ile Ife	9	female	fever	OPD
Ile Ife	23	Male	LCI	OPD
Ile Ife	23	female	LCI	OPD
Ile Ife	38	Male	LCI	OPD
Ile Ife	16	Male	>1 CSI	OPD
Ile Ife	56	female	fever	OPD
Ile Ife	26	female	LCI	OPD
Ile Ife	9	Male	LCI	OPD
Ile Ife	33	Male	LCI	OPD
Ile Ife	38	female	LCI	OPD
Ile Ife	33	female	LCI	OPD
Ile Ife	48	Male	LCI	OPD
Ile Ife	25	female	LCI	OPD
Ile Ife	13	female	LCI	OPD
Ile Ife	33	Male	>1 CSI	OPD
Ile Ife	11	female	LCI	OPD
Ile Ife	11	female	>1 CSI	OPD
Ile Ife	10	female	LCI	OPD
Ile Ife	35	Male	LCI	OPD
Ile Ife	13	female	LCI	OPD
Ile Ife	15	Male	LCI	OPD
Ile Ife	48	Male	fever	OPD
Ile Ife	24	female	LCI	OPD
Ile Ife	15	female	LCI	OPD
Ile Ife	23	Male	LCI	OPD
Ile Ife	15	Male	LCI	OPD
Ile Ife	7	Male	fever	OPD
Ile Ife	6	Male	fb0-6	OPD
Ile Ife	4	female	fb0-6	OPD
Ile Ife	5	Male	fb0-6	OPD
Ile Ife	6	Male	fb0-6	OPD
Ile Ife	2	Male	fb0-6	OPD
Ile Ife	6	female	fb0-6	OPD
Ile Ife	6	female	fb0-6	OPD
Ile Ife	6	Male	fb0-6	OPD
Ile Ife	5	female	fb0-6	OPD
Ile Ife	6	female	fb0-6	OPD
Ile Ife	5	female	fb0-6	OPD
Ile Ife	2	Male	fb0-6	OPD
Ile Ife	6	Male	fb0-6	OPD
Ile Ife	3	female	fb0-6	OPD
Ile Ife	1	Male	fb0-6	OPD
Ile Ife	5	Male	fb0-6	OPD

Ile Ife	2	female	fb0-6	OPD
Ile Ife	1	Male	fb0-6	OPD
Ile Ife	5	Male	fb0-6	OPD
Ile Ife	2	female	fb0-6	OPD
Ile Ife	2	Male	fb0-6	OPD
Ile Ife	1	female	fb0-6	OPD
Ile Ife	2	Male	fb0-6	OPD
Ile Ife	1	Male	fb0-6	OPD
Ile Ife	3	Male	fb0-6	OPD
Ile Ife	1	Male	fb0-6	OPD
Ile Ife	6	female	fb0-6	OPD
Ile Ife	1	female	fb0-6	OPD
Ile Ife	4	female	fb0-6	OPD
Ile Ife	6	Male	fb0-6	OPD
Ile Ife	5	Male	fb0-6	OPD
Ile Ife	6	female	fb0-6	OPD
Ile Ife	3	Male	fb0-6	OPD
Ile Ife	2	Male	fb0-6	OPD
Ile Ife	3	female	fb0-6	OPD
Ile Ife	1	Male	fb0-6	OPD
Ile Ife	2	Male	fb0-6	OPD
Ile Ife	4	female	fb0-6	OPD
Ile Ife	2	female	fb0-6	OPD
Ile Ife	4	female	fb0-6	OPD
Ile Ife	3	female	fb0-6	OPD
Ile Ife	6	female	fb0-6	OPD
Ile Ife	4	Male	fb0-6	OPD
Ile Ife	6	female	fb0-6	OPD
Ile Ife	2	female	fb0-6	OPD
Ile Ife	6	female	fb0-6	OPD
Ile Ife	3	female	fb0-6	OPD
Ile Ife	2	female	fb0-6	OPD
Ile Ife	4	Male	fb0-6	OPD
Ile Ife	1	Male	fb0-6	OPD
Ile Ife	1	female	fb0-6	OPD
Ile Ife	6	Male	fb0-6	OPD
Ile Ife	2	Male	fb0-6	OPD
Ile Ife	4	Male	fb0-6	OPD
Ile Ife	3	female	fb0-6	OPD
Ile Ife	6	Male	fb0-6	OPD
Ile Ife	4	Male	fb0-6	OPD
Ile Ife	5	female	fb0-6	OPD
Ile Ife	5	female	fb0-6	OPD
Ile Ife	1	Male	fb0-6	OPD
Ile Ife	1	Male	fb0-6	OPD
Ile Ife	4	Male	fb0-6	OPD
Ile Ife	1	Male	fb0-6	OPD
Ile Ife	6	Male	fb0-6	OPD
Ile Ife	6	female	fb0-6	OPD
Ile Ife	4	female	fb0-6	OPD

Ile Ife	2	Male	fb0-6	OPD
Ile Ife	4	female	fb0-6	OPD
Ile Ife	5	female	fb0-6	OPD
Ile Ife	1	female	fb0-6	OPD
Ile Ife	2	Male	fb0-6	OPD
Ile Ife	5	Male	fb0-6	OPD
Ile Ife	4	Male	fb0-6	OPD
Ile Ife	2	Male	fb0-6	OPD
Ile Ife	4	female	fb0-6	OPD
Ile Ife	2	Male	fb0-6	OPD
Ile Ife	3	female	fb0-6	OPD
Ile Ife	5	Male	fb0-6	OPD
Ile Ife	4	female	fb0-6	OPD
Ile Ife	3	Male	fb0-6	OPD
Ile Ife	2	Male	fb0-6	OPD
Ile Ife	6	female	fb0-6	OPD
Ile Ife	2	Male	fb0-6	OPD
Ile Ife	6	female	fb0-6	OPD
Ile Ife	1	Male	fb0-6	OPD
Ile Ife	1	female	fb0-6	OPD
Ile Ife	5	Male	fb0-6	OPD
Ile Ife	5	female	fb0-6	OPD
Ile Ife	5	Male	fb0-6	OPD
Ile Ife	1	Male	fb0-6	OPD
Ile Ife	5	female	fb0-6	OPD
Ile Ife	2	Male	fb0-6	OPD
Ile Ife	1	female	fb0-6	OPD
Ile Ife	1	female	fb0-6	OPD
Ile Ife	4	female	fb0-6	OPD
Ile Ife	4	female	fb0-6	OPD
Ile Ife	3	female	fb0-6	OPD
Ile Ife	1	female	fb0-6	OPD
Ile Ife	2	Male	fb0-6	OPD
Ile Ife	5	Male	fb0-6	OPD
Ile Ife	1	female	fb0-6	OPD
Ile Ife	1	Male	fb0-6	OPD
Ile Ife	3	Male	fb0-6	OPD
Ile Ife	2	Male	fb0-6	OPD
Ile Ife	1	female	fb0-6	OPD
Ile Ife	2	Male	fb0-6	OPD
Ile Ife	2	Male	fb0-6	OPD
Ile Ife	5	Male	fb0-6	OPD
Ile Ife	2	female	fb0-6	OPD
Ile Ife	5	Male	fb0-6	OPD
Ile Ife	5	Male	fb0-6	OPD
Ile Ife	3	Male	fb0-6	OPD
Ile Ife	2	female	fb0-6	OPD
Ile Ife	3	Male	fb0-6	OPD
Ile Ife	4	female	fb0-6	OPD
Ile Ife	1	Male	fb0-6	OPD

Ile Ife	2	Male	fb0-6	OPD
Ile Ife	1	female	fb0-6	OPD
Ile Ife	1	female	fb0-6	OPD
Ile Ife	3	Male	fb0-6	OPD
Ile Ife	4	Male	fb0-6	OPD
Ile Ife	1	female	fb0-6	OPD
Ile Ife	4	Male	fb0-6	OPD
Ile Ife	5	female	fb0-6	OPD
Ile Ife	3	female	fb0-6	OPD
Ile Ife	3	female	fb0-6	OPD
Ile Ife	6	female	fb0-6	OPD
Ile Ife	3	female	fb0-6	OPD
Ile Ife	2	female	fb0-6	OPD
Ile Ife	6	female	fb0-6	OPD
Ile Ife	5	Male	fb0-6	OPD
Ile Ife	6	female	fb0-6	OPD
Ile Ife	5	female	fb0-6	OPD
Ile Ife	6	female	fb0-6	OPD
Ile Ife	6	female	fb0-6	OPD
Ile Ife	6	female	fb0-6	OPD
Ile Ife	5	Male	fb0-6	OPD
Ile Ife	4	female	fb0-6	OPD
Ile Ife	1	female	fb0-6	OPD
Ile Ife	6	Male	fb0-6	OPD
Ile Ife	5	female	fb0-6	OPD
Ile Ife	3	Male	fb0-6	OPD
Ile Ife	1	Male	fb0-6	OPD
Ile Ife	3	Male	fb0-6	OPD
Ile Ife	6	Male	fb0-6	OPD
Ile Ife	6	Male	fb0-6	OPD
Ile Ife	2	Male	fb0-6	OPD
Ile Ife	3	Male	fb0-6	OPD
Ile Ife	1	female	fb0-6	OPD
Ile Ife	1	Male	fb0-6	OPD
Ile Ife	6	Male	fb0-6	OPD
Ile Ife	3	female	fb0-6	OPD
Ile Ife	1	Male	fb0-6	OPD
Ile Ife	1	female	fb0-6	OPD
Ile Ife	6	female	fb0-6	OPD
Ile Ife	3	female	fb0-6	OPD
Ile Ife	4	female	fb0-6	OPD
Ile Ife	5	Male	fb0-6	OPD
Ile Ife	2	female	fb0-6	OPD
Ile Ife	4	female	fb0-6	OPD
Ile Ife	9	female	FB 7-59	OPD
Ile Ife	23	female	FB 7-59	OPD
Ile Ife	18	female	FB 7-59	OPD
Ile Ife	8	female	FB 7-59	OPD
Ile Ife	9	Male	FB 7-59	OPD
Ile Ife	31	Male	FB 7-59	OPD

Ile Ife	20	female	FB 7-59	OPD
Ile Ife	12	female	FB 7-59	OPD
Ile Ife	14	female	FB 7-59	OPD
Ile Ife	10	Male	FB 7-59	OPD
Ile Ife	12	Male	FB 7-59	OPD
Ile Ife	21	female	FB 7-59	OPD
Ile Ife	12	Male	FB 7-59	OPD
Ile Ife	11	female	FB 7-59	OPD
Ile Ife	8	Male	FB 7-59	OPD
Ile Ife	8	female	FB 7-59	OPD
Ile Ife	24	Male	FB 7-59	OPD
Ile Ife	10	Male	FB 7-59	OPD
Ile Ife	20	Male	FB 7-59	OPD
Ile Ife	20	female	FB 7-59	OPD
Ile Ife	7	Male	FB 7-59	OPD
Ile Ife	9	female	FB 7-59	OPD
Ile Ife	19	female	FB 7-59	OPD
Ile Ife	13	female	FB 7-59	OPD
Ile Ife	14	female	FB 7-59	OPD
Ile Ife	9	female	FB 7-59	OPD
Ile Ife	11	female	FB 7-59	OPD
Ile Ife	12	female	FB 7-59	OPD
Ile Ife	9	female	FB 7-59	OPD
Ile Ife	17	female	FB 7-59	OPD
Ile Ife	21	female	FB 7-59	OPD
Ile Ife	16	Male	FB 7-59	OPD
Ile Ife	21	Male	FB 7-59	OPD
Ile Ife	7	female	FB 7-59	OPD
Ile Ife	22	female	FB 7-59	OPD
Ile Ife	18	female	FB 7-59	OPD
Ile Ife	23	female	FB 7-59	OPD
Ile Ife	52	female	FB 7-59	OPD
Ile Ife	35	Male	FB 7-59	OPD
Ile Ife	9	female	FB 7-59	OPD
Ile Ife	31	Male	FB 7-59	OPD
Ile Ife	9	Male	FB 7-59	OPD
Ile Ife	7	Male	FB 7-59	OPD
Ile Ife	14	Male	FB 7-59	OPD
Ile Ife	9	Male	FB 7-59	OPD
Ile Ife	10	female	FB 7-59	OPD
Ile Ife	16	Male	FB 7-59	OPD
Ile Ife	24	female	FB 7-59	OPD
Ile Ife	16	Male	FB 7-59	OPD
Ile Ife	20	female	FB 7-59	OPD
Ile Ife	30	female	FB 7-59	OPD
Ile Ife	7	female	FB 7-59	OPD
Ile Ife	39	Male	FB 7-59	OPD
Ile Ife	15	female	FB 7-59	OPD
Ile Ife	26	Male	FB 7-59	OPD
Ile Ife	12	female	FB 7-59	OPD

Ile Ife	13	Male	FB 7-59	OPD
Ile Ife	15	female	FB 7-59	OPD
Ile Ife	33	Male	FB 7-59	OPD
Ile Ife	30	female	FB 7-59	OPD
Ile Ife	22	Male	FB 7-59	OPD
Ile Ife	23	female	FB 7-59	OPD
Ile Ife	12	Male	FB 7-59	OPD
Ile Ife	8	Male	FB 7-59	OPD
Ile Ife	18	female	FB 7-59	OPD
Ile Ife	20	female	FB 7-59	OPD
Ile Ife	12	Male	FB 7-59	OPD
Ile Ife	10	female	FB 7-59	OPD
Ile Ife	17	female	FB 7-59	OPD
Ile Ife	8	Male	FB 7-59	OPD
Ile Ife	12	Male	FB 7-59	OPD
Ile Ife	19	Male	FB 7-59	OPD
Ile Ife	8	Male	FB 7-59	OPD
Ile Ife	9	female	FB 7-59	OPD
Ile Ife	20	female	FB 7-59	OPD
Ile Ife	16	Male	FB 7-59	OPD
Ile Ife	20	Male	FB 7-59	OPD
Ile Ife	12	Male	FB 7-59	OPD
Ile Ife	19	Male	FB 7-59	OPD
Ile Ife	29	female	FB 7-59	OPD
Ile Ife	16	female	FB 7-59	OPD
Ile Ife	16	Male	FB 7-59	OPD
Ile Ife	9	female	FB 7-59	OPD
Ile Ife	26	Male	FB 7-59	OPD
Ile Ife	14	female	FB 7-59	OPD
Ile Ife	13	Male	FB 7-59	OPD
Ile Ife	31	Male	FB 7-59	OPD
Ile Ife	7	Male	FB 7-59	OPD
Ile Ife	22	Male	FB 7-59	OPD
Ile Ife	17	Male	FB 7-59	OPD
Ile Ife	15	female	FB 7-59	OPD
Ile Ife	8	Male	FB 7-59	OPD
Ile Ife	39	Male	FB 7-59	OPD
Ile Ife	9	Male	FB 7-59	OPD
Ile Ife	20	Male	FB 7-59	OPD
Ile Ife	20	female	FB 7-59	OPD
Ile Ife	11	female	FB 7-59	OPD
Ile Ife	8	Male	FB 7-59	OPD
Ile Ife	8	Male	FB 7-59	OPD
Ile Ife	7	Male	FB 7-59	OPD
Ile Ife	14	Male	FB 7-59	OPD
Ile Ife	7	female	FB 7-59	OPD
Ile Ife	20	female	FB 7-59	OPD
Ile Ife	19	female	FB 7-59	OPD
Ile Ife	11	Male	FB 7-59	OPD
Ile Ife	8	Male	FB 7-59	OPD

Ile Ife	10	female	FB 7-59	OPD
Ile Ife	10	female	FB 7-59	OPD
Ile Ife	12	female	FB 7-59	OPD
Ile Ife	22	female	FB 7-59	OPD
Ile Ife	17	female	FB 7-59	OPD
Ile Ife	9	female	FB 7-59	OPD
Ile Ife	11	female	FB 7-59	OPD
Ile Ife	19	female	FB 7-59	OPD
Ile Ife	37	Male	FB 7-59	OPD
Ile Ife	42	Male	FB 7-59	OPD
Ile Ife	11	Male	FB 7-59	OPD
Ile Ife	21	Male	FB 7-59	OPD
Ile Ife	8	female	FB 7-59	OPD
Ile Ife	11	female	FB 7-59	OPD
Ile Ife	20	female	FB 7-59	OPD
Ile Ife	8	Male	FB 7-59	OPD
Ile Ife	9	Male	FB 7-59	OPD
Ile Ife	22	female	FB 7-59	OPD
Ile Ife	9	Male	FB 7-59	OPD
Ile Ife	17	female	FB 7-59	OPD
Ile Ife	22	Male	FB 7-59	OPD
Ile Ife	13	Male	FB 7-59	OPD
Ile Ife	18	Male	FB 7-59	OPD
Ile Ife	53	Male	FB 7-59	OPD
Ile Ife	23	Male	FB 7-59	OPD
Ile Ife	25	Male	FB 7-59	OPD
Ile Ife	7	female	FB 7-59	OPD
Ile Ife	11	Male	FB 7-59	OPD
Ile Ife	29	Male	FB 7-59	OPD
Ile Ife	8	female	FB 7-59	OPD
Ile Ife	29	female	FB 7-59	OPD
Ile Ife	48	female	FB 7-59	OPD
Ile Ife	10	female	FB 7-59	OPD
Ile Ife	26	female	FB 7-59	OPD
Ile Ife	7	female	FB 7-59	OPD
Ile Ife	8	female	FB 7-59	OPD
Ile Ife	10	Male	FB 7-59	OPD
Ile Ife	9	female	FB 7-59	OPD
Ile Ife	12	Male	FB 7-59	OPD
Ile Ife	13	Male	FB 7-59	OPD
Ile Ife	11	Male	FB 7-59	OPD
Ile Ife	13	female	FB 7-59	OPD
Ile Ife	22	Male	FB 7-59	OPD
Ile Ife	13	female	FB 7-59	OPD
Ile Ife	19	Male	FB 7-59	OPD
Ile Ife	18	Male	FB 7-59	OPD
Ile Ife	16	female	FB 7-59	OPD
Ile Ife	7	Male	FB 7-59	OPD
Ile Ife	21	Male	FB 7-59	OPD
Ile Ife	10	Male	FB 7-59	OPD

Ile Ife	7	female	FB 7-59	OPD
Ile Ife	23	Male	FB 7-59	OPD
Ile Ife	23	female	FB 7-59	OPD
Ile Ife	10	female	FB 7-59	OPD
Zaria	3	Male	fever	OPD
Zaria	3	female	fever	OPD
Zaria	1	female	fever	OPD
Zaria	1	Male	fever	OPD
Zaria	1	Male	fever	OPD
Zaria	2	female	fever	OPD
Zaria	3	Male	fever	OPD
Zaria	5	female	fever	OPD
Zaria	1	female	Hypo	OPD
Zaria	1	female	movement on stimula	OPD
Zaria	2	female	movement on stimula	OPD
Zaria	3	Male	fever	OPD
Zaria	4	female	poor feeding	OPD
Zaria	1	female	>1 CSI	OPD
Zaria	1	Male	>1 CSI	OPD
Zaria	1	Male	Hypo	OPD
Zaria	5	female	fever	OPD
Zaria	3	female	Hypo	OPD
Zaria	3	female	fever	OPD
Zaria	5	Male	>1 CSI	OPD
Zaria	6	Male	>1 CSI	OPD
Zaria	2	Male	fever	OPD
Zaria	1	Male	fever	OPD
Zaria	3	female	Hypo	OPD
Zaria	2	female	fever	OPD
Zaria	2	Male	fever	OPD
Zaria	4	female	movement on stimula	OPD
Zaria	3	female	>1 CSI	OPD
Zaria	6	female	Hypo	OPD
Zaria	2	Male	fever	OPD
Zaria	2	Male	LCI	OPD
Zaria	1	Male	Hypo	OPD
Zaria	3	female	fever	OPD
Zaria	1	female	>1 CSI	OPD
Zaria	1	Male	Hypo	OPD
Zaria	6	Male	Hypo	OPD
Zaria	2	female	>1 CSI	OPD
Zaria	4	Male	fever	OPD
Zaria	1	female	Hypo	OPD
Zaria	1	Male	Hypo	OPD
Zaria	1	Male	fever	OPD
Zaria	1	Male	Hypo	OPD
Zaria	6	Male	poor feeding	OPD
Zaria	2	Male	poor feeding	OPD
Zaria	1	female	LCI	OPD
Zaria	1	female	Hypo	OPD

Zaria	6	Male	Hypo	OPD
Zaria	3	female	Hypo	OPD
Zaria	3	Male	>1 CSI	OPD
Zaria	3	female	fever	OPD
Zaria	2	female	fever	OPD
Zaria	1	Male	>1 CSI	OPD
Zaria	4	Male	LCI	OPD
Zaria	3	Male	>1 CSI	OPD
Zaria	1	female	poor feeding	OPD
Zaria	3	female	fever	OPD
Zaria	1	Male	LCI	OPD
Zaria	1	Male	>1 CSI	OPD
Zaria	4	female	LCI	OPD
Zaria	6	Male	LCI	OPD
Zaria	4	Male	fever	OPD
Zaria	6	Male	LCI	OPD
Zaria	4	Male	LCI	OPD
Zaria	3	female	>1 CSI	OPD
Zaria	2	Male	fever	OPD
Zaria	1	female	Hypo	OPD
Zaria	1	Male	fever	OPD
Zaria	2	Male	fever	OPD
Zaria	1	female	fever	OPD
Zaria	3	Male	fever	OPD
Zaria	1	Male	>1 CSI	OPD
Zaria	6	Male	fever	OPD
Zaria	2	female	fever	OPD
Zaria	1	Male	fever	OPD
Zaria	1	female	fever	OPD
Zaria	1	Male	fever	OPD
Zaria	2	female	fever	OPD
Zaria	3	Male	LCI	OPD
Zaria	1	Male	fever	OPD
Zaria	3	female	fever	OPD
Zaria	4	Male	fever	OPD
Zaria	2	Male	fever	OPD
Zaria	1	female	fever	OPD
Zaria	1	Male	fever	OPD
Zaria	2	female	fever	OPD
Zaria	3	Male	fever	OPD
Zaria	5	female	LCI	OPD
Zaria	3	Male	fever	OPD
Zaria	4	female	fever	OPD
Zaria	3	female	fever	OPD
Zaria	2	female	poor feeding	OPD
Zaria	4	Male	movement on stimula	OPD
Zaria	3	Male	>1 CSI	OPD
Zaria	3	Male	>1 CSI	OPD
Zaria	3	Male	fever	OPD
Zaria	1	female	Hypo	OPD

Zaria	1	female	fever	OPD
Zaria	5	Male	>1 CSI	OPD
Zaria	3	female	movement on stimula	OPD
Zaria	4	Male	LCI	OPD
Zaria	1	female	Hypo	OPD
Zaria	3	Male	fever	OPD
Zaria	1	Male	>1 CSI	OPD
Zaria	3	female	Hypo	OPD
Zaria	5	Male	LCI	OPD
Zaria	3	Male	>1 CSI	OPD
Zaria	2	Male	LCI	OPD
Zaria	2	female	Hypo	OPD
Zaria	1	female	Hypo	OPD
Zaria	5	female	fever	OPD
Zaria	6	female	LCI	OPD
Zaria	3	Male	>1 CSI	OPD
Zaria	3	female	Hypo	OPD
Zaria	5	Male	Hypo	OPD
Zaria	1	female	>1 CSI	OPD
Zaria	1	Male	Hypo	OPD
Zaria	1	female	Hypo	OPD
Zaria	4	female	LCI	OPD
Zaria	5	Male	LCI	OPD
Zaria	1	female	Hypo	OPD
Zaria	2	Male	poor feeding	OPD
Zaria	3	Male	movement on stimula	OPD
Zaria	6	female	fever	OPD
Zaria	2	female	>1 CSI	OPD
Zaria	1	female	Hypo	OPD
Zaria	4	female	poor feeding	OPD
Zaria	2	female	>1 CSI	OPD
Zaria	6	female	poor feeding	OPD
Zaria	2	female	poor feeding	OPD
Zaria	4	Male	fever	OPD
Zaria	4	female	>1 CSI	OPD
Zaria	1	female	>1 CSI	OPD
Zaria	1	Male	Hypo	OPD
Zaria	3	female	fever	OPD
Zaria	1	Male	fever	OPD
Zaria	1	female	Hypo	OPD
Zaria	2	Male	poor feeding	OPD
Zaria	1	Male	Hypo	OPD
Zaria	1	Male	poor feeding	OPD
Zaria	1	female	Hypo	OPD
Zaria	1	Male	LCI	OPD
Zaria	4	Male	fever	OPD
Zaria	5	female	fever	OPD
Zaria	3	Male	>1 CSI	OPD
Zaria	1	Male	LCI	OPD
Zaria	2	Male	fever	OPD

Zaria	3	Male	poor feeding	OPD
Zaria	6	female	fever	OPD
Zaria	5	Male	>1 CSI	OPD
Zaria	1	Male	LCI	OPD
Zaria	1	female	Hypo	OPD
Zaria	2	female	fever	OPD
Zaria	2	female	fever	OPD
Zaria	1	Male	Hypo	OPD
Zaria	2	Male	poor feeding	OPD
Zaria	1	female	>1 CSI	OPD
Zaria	1	Male	Hypo	OPD
Zaria	3	Male	poor feeding	OPD
Zaria	6	Male	>1 CSI	OPD
Zaria	1	Male	Hypo	OPD
Zaria	4	Male	poor feeding	OPD
Zaria	3	Male	Hypo	OPD
Zaria	3	female	Hypo	OPD
Zaria	1	female	>1 CSI	OPD
Zaria	1	female	Hypo	OPD
Zaria	1	female	>1 CSI	OPD
Zaria	2	female	Hypo	OPD
Zaria	1	Male	>1 CSI	OPD
Zaria	1	female	>1 CSI	OPD
Zaria	3	female	poor feeding	OPD
Zaria	4	Male	poor feeding	OPD
Zaria	1	Male	>1 CSI	OPD
Zaria	4	female	Hypo	OPD
Zaria	1	female	Hypo	OPD
Zaria	1	Male	Hypo	OPD
Zaria	4	Male	poor feeding	OPD
Zaria	5	female	LCI	OPD
Zaria	1	Male	Hypo	OPD
Zaria	1	female	fever	OPD
Zaria	6	female	>1 CSI	OPD
Zaria	1	female	Hypo	OPD
Zaria	3	Male	>1 CSI	OPD
Zaria	1	female	Hypo	OPD
Zaria	1	Male	poor feeding	OPD
Zaria	3	Male	LCI	OPD
Zaria	3	Male	poor feeding	OPD
Zaria	2	female	fever	OPD
Zaria	4	Male	fever	OPD
Zaria	6	Male	>1 CSI	OPD
Zaria	1	female	>1 CSI	OPD
Zaria	2	Male	fever	OPD
Zaria	3	female	>1 CSI	OPD
Zaria	5	Male	>1 CSI	OPD
Zaria	2	female	fever	OPD
Zaria	2	Male	LCI	OPD
Zaria	1	Male	Hypo	OPD

Zaria	3	Male	>1 CSI	OPD
Zaria	3	Male	fever	OPD
Zaria	3	female	>1 CSI	OPD
Zaria	2	female	fever	OPD
Zaria	2	Male	fever	OPD
Zaria	2	Male	fever	OPD
Zaria	2	Male	fever	OPD
Zaria	2	Male	fever	OPD
Zaria	3	female	fever	OPD
Zaria	2	female	LCI	OPD
Zaria	6	female	fever	OPD
Zaria	1	female	fever	OPD
Zaria	3	Male	poor feeding	OPD
Zaria	3	Male	poor feeding	OPD
Zaria	3	Male	LCI	OPD
Zaria	3	female	poor feeding	OPD
Zaria	3	Male	poor feeding	OPD
Zaria	2	Male	fever	OPD
Zaria	5	Male	poor feeding	OPD
Zaria	6	Male	LCI	OPD
Zaria	1	Male	fever	OPD
Zaria	3	female	fever	OPD
Zaria	2	Male	fever	OPD
Zaria	1	Male	LCI	OPD
Zaria	3	female	fever	OPD
Zaria	3	Male	LCI	OPD
Zaria	3	Male	poor feeding	OPD
Zaria	3	Male	fever	OPD
Zaria	6	Male	fever	OPD
Zaria	3	Male	poor feeding	OPD
Zaria	6	female	poor feeding	OPD
Zaria	3	female	fever	OPD
Zaria	5	female	fever	OPD
Zaria	3	Male	fever	OPD
Zaria	2	Male	fever	OPD
Zaria	2	Male	fever	OPD
Zaria	4	female	fever	OPD
Zaria	1	Male	fever	OPD
Zaria	4	Male	fever	OPD
Zaria	2	female	poor feeding	OPD
Zaria	1	female	fever	OPD
Zaria	5	female	>1 CSI	OPD
Zaria	3	female	fever	OPD
Zaria	8	female	fever	OPD
Zaria	20	Male	poor feeding	OPD
Zaria	13	Male	fever	OPD
Zaria	32	female	fever	OPD
Zaria	16	Male	>1 CSI	OPD
Zaria	7	Male	fever	OPD
Zaria	25	Male	fever	OPD

Zaria	21	female	fever	OPD
Zaria	14	female	>1 CSI	OPD
Zaria	44	female	fever	OPD
Zaria	14	Male	>1 CSI	OPD
Zaria	52	Male	poor feeding	OPD
Zaria	7	Male	>1 CSI	OPD
Zaria	11	female	fever	OPD
Zaria	28	female	fever	OPD
Zaria	16	Male	fever	OPD
Zaria	21	Male	poor feeding	OPD
Zaria	7	female	fever	OPD
Zaria	14	female	>1 CSI	OPD
Zaria	49	female	fever	OPD
Zaria	28	Male	fever	OPD
Zaria	48	female	fever	OPD
Zaria	11	female	fever	OPD
Zaria	6	female	fever	OPD
Zaria	19	Male	>1 CSI	OPD
Zaria	7	Male	movement on stimula	OPD
Zaria	1	female	LCI	OPD
Zaria	1	Male	LCI	OPD
Zaria	7	Male	fever	OPD
Zaria	36	Male	LCI	OPD
Zaria	48	female	>1 CSI	OPD
Zaria	40	Male	fever	OPD
Zaria	35	Male	fever	OPD
Zaria	21	Male	>1 CSI	OPD
Zaria	32	Male	poor feeding	OPD
Zaria	10	Male	poor feeding	OPD
Zaria	54	Male	fever	OPD
Zaria	13	female	Hypo	OPD
Zaria	47	Male	fever	OPD
Zaria	8	Male	fever	OPD
Zaria	59	Male	fever	OPD
Zaria	14	Male	LCI	OPD
Zaria	20	female	fever	OPD
Zaria	42	female	fever	OPD
Zaria	18	Male	fever	OPD
Zaria	1	female	fever	OPD
Zaria	17	female	poor feeding	OPD
Zaria	52	female	fever	OPD
Zaria	46	Male	fever	OPD
Zaria	10	Male	fever	OPD
Zaria	21	Male	fever	OPD
Zaria	15	Male	poor feeding	OPD
Zaria	10	female	LCI	OPD
Zaria	50	female	fever	OPD
Zaria	15	Male	fever	OPD
Zaria	47	female	fever	OPD
Zaria	21	Male	fever	OPD

Zaria	13	female	>1 CSI	OPD
Zaria	30	Male	fever	OPD
Zaria	31	Male	fever	OPD
Zaria	14	female	fever	OPD
Zaria	10	female	fever	OPD
Zaria	37	Male	poor feeding	OPD
Zaria	11	female	fever	OPD
Zaria	9	Male	poor feeding	OPD
Zaria	12	Male	fever	OPD
Zaria	1	female	LCI	OPD
Zaria	42	female	LCI	OPD
Zaria	47	female	poor feeding	OPD
Zaria	12	female	movement on stimula	OPD
Zaria	28	female	fever	OPD
Zaria	11	Male	>1 CSI	OPD
Zaria	21	female	fever	OPD
Zaria	55	Male	LCI	OPD
Zaria	20	Male	fever	OPD
Zaria	13	female	fever	OPD
Zaria	56	Male	poor feeding	OPD
Zaria	8	female	fever	OPD
Zaria	43	female	fever	OPD
Zaria	23	female	fever	OPD
Zaria	9	female	fever	OPD
Zaria	28	Male	fever	OPD
Zaria	31	female	fever	OPD
Zaria	38	Male	LCI	OPD
Zaria	18	Male	Hypo	OPD
Zaria	11	female	fever	OPD
Zaria	14	female	LCI	OPD
Zaria	45	Male	fever	OPD
Zaria	17	female	fever	OPD
Zaria	21	female	LCI	OPD
Zaria	49	Male	LCI	OPD
Zaria	8	female	fever	OPD
Zaria	13	Male	poor feeding	OPD
Zaria	7	Male	movement on stimula	OPD
Zaria	27	female	LCI	OPD
Zaria	11	female	fever	OPD
Zaria	49	female	poor feeding	OPD
Zaria	42	female	LCI	OPD
Zaria	9	female	fever	OPD
Zaria	8	Male	>1 CSI	OPD
Zaria	29	Male	fever	OPD
Zaria	40	female	fever	OPD
Zaria	7	female	LCI	OPD
Zaria	28	female	LCI	OPD
Zaria	10	Male	>1 CSI	OPD
Zaria	22	female	fever	OPD
Zaria	36	Male	LCI	OPD

Zaria	40	female	fever	OPD
Zaria	3	female	LCI	OPD
Zaria	28	Male	poor feeding	OPD
Zaria	13	Male	poor feeding	OPD
Zaria	19	Male	LCI	OPD
Zaria	12	female	>1 CSI	OPD
Zaria	53	Male	LCI	OPD
Zaria	41	female	movement on stimula	OPD
Zaria	7	Male	>1 CSI	OPD
Zaria	14	Male	fever	OPD
Zaria	21	female	LCI	OPD
Zaria	8	Male	>1 CSI	OPD
Zaria	15	Male	fever	OPD
Zaria	9	Male	fever	OPD
Zaria	2	female	LCI	OPD
Zaria	13	Male	fever	OPD
Zaria	11	female	fever	OPD
Zaria	15	Male	fever	OPD
Zaria	30	female	LCI	OPD
Zaria	15	Male	poor feeding	OPD
Zaria	14	female	LCI	OPD
Zaria	21	female	>1 CSI	OPD
Zaria	17	female	LCI	OPD
Zaria	8	female	fever	OPD
Zaria	7	Male	fever	OPD
Zaria	18	female	fever	OPD
Zaria	28	Male	LCI	OPD
Zaria	31	Male	LCI	OPD
Zaria	14	female	fever	OPD
Zaria	8	female	fever	OPD
Zaria	1	Male	>1 CSI	OPD
Zaria	24	Male	fever	OPD
Zaria	22	Male	poor feeding	OPD
Zaria	19	female	fever	OPD
Zaria	11	Male	fever	OPD
Zaria	45	Male	LCI	OPD
Zaria	12	female	fever	OPD
Zaria	8	Male	>1 CSI	OPD
Zaria	12	female	LCI	OPD
Zaria	14	Male	fever	OPD
Zaria	2	female	fever	OPD
Zaria	16	Male	fever	OPD
Zaria	15	Male	LCI	OPD
Zaria	15	Male	LCI	OPD
Zaria	15	female	poor feeding	OPD
Zaria	48	female	>1 CSI	OPD
Zaria	48	female	>1 CSI	OPD
Zaria	12	female	fever	OPD
Zaria	21	Male	fever	OPD
Zaria	24	female	LCI	OPD

Zaria	24	female	>1 CSI	OPD
Zaria	36	Male	LCI	OPD
Zaria	8	female	fever	OPD
Zaria	10	Male	>1 CSI	OPD
Zaria	16	female	fever	OPD
Zaria	19	Male	LCI	OPD
Zaria	38	female	poor feeding	OPD
Zaria	20	female	fever	OPD
Zaria	22	Male	movement on stimula	OPD
Zaria	23	Male	LCI	OPD
Zaria	13	female	poor feeding	OPD
Zaria	19	Male	>1 CSI	OPD
Zaria	14	female	>1 CSI	OPD
Zaria	30	Male	LCI	OPD
Zaria	10	Male	fever	OPD
Zaria	35	female	poor feeding	OPD
Zaria	10	female	>1 CSI	OPD
Zaria	15	female	LCI	OPD
Zaria	50	female	fever	OPD
Zaria	23	Male	poor feeding	OPD
Zaria	16	Male	>1 CSI	OPD
Zaria	17	Male	poor feeding	OPD
Zaria	11	female	LCI	OPD
Zaria	25	Male	fever	OPD
Zaria	7	Male	LCI	OPD
Zaria	42	female	>1 CSI	OPD
Zaria	19	Male	poor feeding	OPD
Zaria	7	female	LCI	OPD
Zaria	26	female	>1 CSI	OPD
Zaria	1	Male	Hypo	OPD
Zaria	19	Male	poor feeding	OPD
Zaria	23	Male	fever	OPD
Zaria	14	Male	>1 CSI	OPD
Zaria	17	Male	LCI	OPD
Zaria	14	female	poor feeding	OPD
Zaria	10	Male	fever	OPD
Zaria	11	Male	>1 CSI	OPD
Zaria	30	female	fever	OPD
Zaria	4	Male	fever	OPD
Zaria	10	Male	poor feeding	OPD
Zaria	18	Male	poor feeding	OPD
Zaria	16	Male	poor feeding	OPD
Zaria	10	Male	poor feeding	OPD
Zaria	12	Male	poor feeding	OPD
Zaria	25	Male	>1 CSI	OPD
Zaria	13	female	LCI	OPD
Zaria	15	Male	poor feeding	OPD
Zaria	14	female	poor feeding	OPD
Zaria	7	female	poor feeding	OPD
Zaria	15	Male	LCI	OPD

Zaria	36	female	poor feeding	OPD
Zaria	24	Male	LCI	OPD
Zaria	14	Male	fever	OPD
Zaria	19	Male	>1 CSI	OPD
Zaria	24	female	poor feeding	OPD
Zaria	10	female	fever	OPD
Zaria	27	Male	LCI	OPD
Zaria	30	Male	>1 CSI	OPD
Zaria	35	female	fever	OPD
Zaria	45	Male	fever	OPD
Zaria	10	Male	poor feeding	OPD
Zaria	33	female	>1 CSI	OPD
Zaria	8	Male	movement on stimula	OPD
Zaria	16	Male	poor feeding	OPD
Zaria	14	Male	poor feeding	OPD
Zaria	9	Male	poor feeding	OPD
Zaria	21	Male	>1 CSI	OPD
Zaria	11	female	poor feeding	OPD
Zaria	18	Male	poor feeding	OPD
Zaria	1	Male	LCI	OPD
Zaria	12	Male	poor feeding	OPD
Zaria	23	female	>1 CSI	OPD
Zaria	14	Male	poor feeding	OPD
Zaria	14	female	poor feeding	OPD
Zaria	23	Male	fever	OPD
Zaria	27	female	fever	OPD
Zaria	21	Male	LCI	OPD
Zaria	3	Male	>1 CSI	OPD
Zaria	9	female	LCI	OPD
Zaria	29	Male	poor feeding	OPD
Zaria	10	female	>1 CSI	OPD
Zaria	9	female	>1 CSI	OPD
Zaria	14	Male	poor feeding	OPD
Zaria	7	female	poor feeding	OPD
Zaria	22	Male	fever	OPD
Zaria	9	female	>1 CSI	OPD
Zaria	16	Male	>1 CSI	OPD
Zaria	20	female	LCI	OPD
Zaria	32	Male	>1 CSI	OPD
Zaria	41	female	>1 CSI	OPD
Zaria	3	Male	convulsions	OPD
Zaria	29	Male	LCI	OPD
Zaria	12	Male	LCI	OPD
Zaria	9	Male	>1 CSI	OPD
Zaria	9	Male	>1 CSI	OPD
Zaria	8	Male	LCI	OPD
Zaria	10	female	>1 CSI	OPD
Zaria	16	Male	>1 CSI	OPD
Zaria	35	Male	poor feeding	OPD
Zaria	9	female	LCI	OPD

Zaria	22	Male	LCI	OPD
Zaria	23	female	>1 CSI	OPD
Zaria	13	Male	>1 CSI	OPD
Zaria	8	female	fever	OPD
Zaria	14	Male	>1 CSI	OPD
Zaria	59	Male	fever	OPD
Zaria	41	female	fever	OPD
Zaria	41	female	>1 CSI	OPD
Zaria	14	female	>1 CSI	OPD
Zaria	10	female	fever	OPD
Zaria	9	Male	>1 CSI	OPD
Zaria	39	Male	LCI	OPD
Zaria	14	Male	fever	OPD
Zaria	20	female	fever	OPD
Zaria	15	female	LCI	OPD
Zaria	23	Male	LCI	OPD
Zaria	7	Male	fever	OPD
Zaria	7	Male	fever	OPD
Zaria	17	female	fever	OPD
Zaria	23	female	fever	OPD
Zaria	10	Male	LCI	OPD
Zaria	7	female	fever	OPD
Zaria	29	Male	poor feeding	OPD
Zaria	1	Male	fb0-6	OPD
Zaria	5	Male	fb0-6	OPD
Zaria	3	Male	fb0-6	OPD
Zaria	3	Male	fb0-6	OPD
Zaria	4	Male	fb0-6	OPD
Zaria	2	Male	fb0-6	OPD
Zaria	3	Male	fb0-6	OPD
Zaria	2	Male	fb0-6	OPD
Zaria	2	female	fb0-6	OPD
Zaria	1	female	fb0-6	OPD
Zaria	2	Male	fb0-6	OPD
Zaria	2	female	fb0-6	OPD
Zaria	3	Male	fb0-6	OPD
Zaria	2	female	fb0-6	OPD
Zaria	1	Male	fb0-6	OPD
Zaria	4	female	fb0-6	OPD
Zaria	5	Male	fb0-6	OPD
Zaria	1	female	fb0-6	OPD
Zaria	4	female	fb0-6	OPD
Zaria	3	female	fb0-6	OPD
Zaria	2	Male	fb0-6	OPD
Zaria	4	Male	fb0-6	OPD
Zaria	1	female	fb0-6	OPD
Zaria	2	female	fb0-6	OPD
Zaria	5	female	fb0-6	OPD
Zaria	2	Male	fb0-6	OPD
Zaria	6	female	fb0-6	OPD

Zaria	1	Male	fb0-6	OPD
Zaria	2	Male	fb0-6	OPD
Zaria	1	Male	fb0-6	OPD
Zaria	3	female	fb0-6	OPD
Zaria	3	female	fb0-6	OPD
Zaria	1	Male	fb0-6	OPD
Zaria	3	Male	fb0-6	OPD
Zaria	4	female	fb0-6	OPD
Zaria	1	Male	fb0-6	OPD
Zaria	2	Male	fb0-6	OPD
Zaria	3	female	fb0-6	OPD
Zaria	6	Male	fb0-6	OPD
Zaria	3	female	fb0-6	OPD
Zaria	5	female	fb0-6	OPD
Zaria	4	female	fb0-6	OPD
Zaria	3	female	fb0-6	OPD
Zaria	3	female	fb0-6	OPD
Zaria	2	Male	fb0-6	OPD
Zaria	4	Male	fb0-6	OPD
Zaria	1	Male	fb0-6	OPD
Zaria	5	Male	fb0-6	OPD
Zaria	1	female	fb0-6	OPD
Zaria	5	Male	fb0-6	OPD
Zaria	5	female	fb0-6	OPD
Zaria	5	Male	fb0-6	OPD
Zaria	4	Male	fb0-6	OPD
Zaria	3	female	fb0-6	OPD
Zaria	3	female	fb0-6	OPD
Zaria	6	female	fb0-6	OPD
Zaria	2	Male	fb0-6	OPD
Zaria	6	Male	fb0-6	OPD
Zaria	1	Male	fb0-6	OPD
Zaria	4	Male	fb0-6	OPD
Zaria	1	female	fb0-6	OPD
Zaria	4	female	fb0-6	OPD
Zaria	1	female	fb0-6	OPD
Zaria	2	Male	fb0-6	OPD
Zaria	6	female	fb0-6	OPD
Zaria	2	female	fb0-6	OPD
Zaria	6	female	fb0-6	OPD
Zaria	6	Male	fb0-6	OPD
Zaria	5	Male	fb0-6	OPD
Zaria	3	Male	fb0-6	OPD
Zaria	3	Male	fb0-6	OPD
Zaria	6	female	fb0-6	OPD
Zaria	3	Male	fb0-6	OPD
Zaria	3	Male	fb0-6	OPD
Zaria	2	Male	fb0-6	OPD
Zaria	3	Male	fb0-6	OPD
Zaria	4	female	fb0-6	OPD

Zaria	5	Male	fb0-6	OPD
Zaria	2	female	fb0-6	OPD
Zaria	4	female	fb0-6	OPD
Zaria	5	female	fb0-6	OPD
Zaria	1	Male	fb0-6	OPD
Zaria	3	Male	fb0-6	OPD
Zaria	4	Male	fb0-6	OPD
Zaria	2	female	fb0-6	OPD
Zaria	3	Male	fb0-6	OPD
Zaria	4	Male	fb0-6	OPD
Zaria	1	female	fb0-6	OPD
Zaria	1	female	fb0-6	OPD
Zaria	3	female	fb0-6	OPD
Zaria	5	Male	fb0-6	OPD
Zaria	4	female	fb0-6	OPD
Zaria	1	Male	fb0-6	OPD
Zaria	6	female	fb0-6	OPD
Zaria	4	Male	fb0-6	OPD
Zaria	1	Male	fb0-6	OPD
Zaria	2	Male	fb0-6	OPD
Zaria	2	female	fb0-6	OPD
Zaria	1	female	fb0-6	OPD
Zaria	5	Male	fb0-6	OPD
Zaria	2	female	fb0-6	OPD
Zaria	3	Male	fb0-6	OPD
Zaria	1	female	fb0-6	OPD
Zaria	1	Male	fb0-6	OPD
Zaria	5	female	fb0-6	OPD
Zaria	3	female	fb0-6	OPD
Zaria	1	Male	fb0-6	OPD
Zaria	4	female	fb0-6	OPD
Zaria	1	Male	fb0-6	OPD
Zaria	2	Male	fb0-6	OPD
Zaria	1	Male	fb0-6	OPD
Zaria	3	Male	fb0-6	OPD
Zaria	1	Male	fb0-6	OPD
Zaria	3	female	fb0-6	OPD
Zaria	1	Male	fb0-6	OPD
Zaria	1	Male	fb0-6	OPD
Zaria	1	Male	fb0-6	OPD
Zaria	1	Male	fb0-6	OPD
Zaria	2	female	fb0-6	OPD
Zaria	1	female	fb0-6	OPD
Zaria	5	Male	fb0-6	OPD
Zaria	1	female	fb0-6	OPD
Zaria	3	female	fb0-6	OPD
Zaria	4	Male	fb0-6	OPD
Zaria	2	female	fb0-6	OPD
Zaria	3	female	fb0-6	OPD
Zaria	6	female	fb0-6	OPD

Zaria	4	Male	fb0-6	OPD
Zaria	3	female	fb0-6	OPD
Zaria	6	Male	fb0-6	OPD
Zaria	4	Male	fb0-6	OPD
Zaria	3	female	fb0-6	OPD
Zaria	2	Male	fb0-6	OPD
Zaria	1	female	fb0-6	OPD
Zaria	1	Male	fb0-6	OPD
Zaria	3	female	fb0-6	OPD
Zaria	1	Male	fb0-6	OPD
Zaria	4	female	fb0-6	OPD
Zaria	3	Male	fb0-6	OPD
Zaria	1	female	fb0-6	OPD
Zaria	3	Male	fb0-6	OPD
Zaria	1	Male	fb0-6	OPD
Zaria	1	female	fb0-6	OPD
Zaria	4	Male	fb0-6	OPD
Zaria	2	female	fb0-6	OPD
Zaria	5	female	fb0-6	OPD
Zaria	3	female	fb0-6	OPD
Zaria	4	female	fb0-6	OPD
Zaria	1	female	fb0-6	OPD
Zaria	1	female	fb0-6	OPD
Zaria	3	female	fb0-6	OPD
Zaria	1	female	fb0-6	OPD
Zaria	3	female	fb0-6	OPD
Zaria	3	female	fb0-6	OPD
Zaria	1	Male	fb0-6	OPD
Zaria	2	female	fb0-6	OPD
Zaria	4	female	fb0-6	OPD
Zaria	2	female	fb0-6	OPD
Zaria	1	Male	fb0-6	OPD
Zaria	1	female	fb0-6	OPD
Zaria	55	Male	FB 7-59	OPD
Zaria	11	female	FB 7-59	OPD
Zaria	15	female	FB 7-59	OPD
Zaria	13	female	FB 7-59	OPD
Zaria	31	Male	FB 7-59	OPD
Zaria	13	Male	FB 7-59	OPD
Zaria	35	female	FB 7-59	OPD
Zaria	13	Male	FB 7-59	OPD
Zaria	12	female	FB 7-59	OPD
Zaria	3	Male	fb0-6	OPD
Zaria	9	Male	FB 7-59	OPD
Zaria	18	Male	FB 7-59	OPD
Zaria	7	female	FB 7-59	OPD
Zaria	14	Male	FB 7-59	OPD
Zaria	14	female	FB 7-59	OPD
Zaria	17	Male	FB 7-59	OPD
Zaria	18	Male	FB 7-59	OPD

Zaria	12	Male	FB 7-59	OPD
Zaria	13	female	FB 7-59	OPD
Zaria	11	female	FB 7-59	OPD
Zaria	22	female	FB 7-59	OPD
Zaria	11	female	FB 7-59	OPD
Zaria	51	Male	FB 7-59	OPD
Zaria	10	Male	FB 7-59	OPD
Zaria	14	Male	FB 7-59	OPD
Zaria	18	Male	FB 7-59	OPD
Zaria	19	female	FB 7-59	OPD
Zaria	12	Male	FB 7-59	OPD
Zaria	13	female	FB 7-59	OPD
Zaria	14	Male	FB 7-59	OPD
Zaria	10	female	FB 7-59	OPD
Zaria	13	Male	FB 7-59	OPD
Zaria	36	Male	FB 7-59	OPD
Zaria	21	Male	FB 7-59	OPD
Zaria	20	female	FB 7-59	OPD
Zaria	10	female	FB 7-59	OPD
Zaria	15	Male	FB 7-59	OPD
Zaria	7	Male	FB 7-59	OPD
Zaria	15	female	FB 7-59	OPD
Zaria	27	female	FB 7-59	OPD
Zaria	57	female	FB 7-59	OPD
Zaria	10	Male	FB 7-59	OPD
Zaria	7	female	FB 7-59	OPD
Zaria	7	Male	FB 7-59	OPD
Zaria	8	Male	FB 7-59	OPD
Zaria	39	Male	FB 7-59	OPD
Zaria	14	Male	FB 7-59	OPD
Zaria	15	Male	FB 7-59	OPD
Zaria	28	Male	FB 7-59	OPD
Zaria	10	Male	FB 7-59	OPD
Zaria	19	female	FB 7-59	OPD
Zaria	15	female	FB 7-59	OPD
Zaria	14	Male	FB 7-59	OPD
Zaria	10	female	FB 7-59	OPD
Zaria	12	Male	FB 7-59	OPD
Zaria	29	Male	FB 7-59	OPD
Zaria	14	female	FB 7-59	OPD
Zaria	25	Male	FB 7-59	OPD
Zaria	12	Male	FB 7-59	OPD
Zaria	18	Male	FB 7-59	OPD
Zaria	18	Male	FB 7-59	OPD
Zaria	23	Male	FB 7-59	OPD
Zaria	15	Male	FB 7-59	OPD
Zaria	46	Male	FB 7-59	OPD
Zaria	3	female	fb0-6	OPD
Zaria	12	Male	FB 7-59	OPD
Zaria	18	female	FB 7-59	OPD

Zaria	10	female	FB 7-59	OPD
Zaria	14	Male	FB 7-59	OPD
Zaria	44	female	FB 7-59	OPD
Zaria	22	female	FB 7-59	OPD
Zaria	59	Male	FB 7-59	OPD
Zaria	14	Male	FB 7-59	OPD
Zaria	22	Male	FB 7-59	OPD
Zaria	7	Male	FB 7-59	OPD
Zaria	3	Male	fb0-6	OPD
Zaria	23	Male	FB 7-59	OPD
Zaria	16	Male	FB 7-59	OPD
Zaria	10	female	FB 7-59	OPD
Zaria	9	Male	FB 7-59	OPD
Zaria	8	Male	FB 7-59	OPD
Zaria	10	Male	FB 7-59	OPD
Zaria	13	female	FB 7-59	OPD
Zaria	21	Male	FB 7-59	OPD
Zaria	59	female	FB 7-59	OPD
Zaria	18	female	FB 7-59	OPD
Zaria	14	Male	FB 7-59	OPD
Zaria	11	female	FB 7-59	OPD
Zaria	30	Male	FB 7-59	OPD
Zaria	13	Male	FB 7-59	OPD
Zaria	28	female	FB 7-59	OPD
Zaria	16	Male	FB 7-59	OPD
Zaria	29	Male	FB 7-59	OPD
Zaria	21	Male	FB 7-59	OPD
Zaria	35	Male	FB 7-59	OPD
Zaria	11	Male	FB 7-59	OPD
Zaria	53	female	FB 7-59	OPD
Zaria	14	Male	FB 7-59	OPD
Zaria	35	Male	FB 7-59	OPD
Zaria	15	Male	FB 7-59	OPD
Zaria	8	Male	FB 7-59	OPD
Zaria	18	Male	FB 7-59	OPD
Zaria	17	Male	FB 7-59	OPD
Zaria	26	female	FB 7-59	OPD
Zaria	15	female	FB 7-59	OPD
Zaria	56	Male	FB 7-59	OPD
Zaria	12	Male	FB 7-59	OPD
Zaria	14	female	FB 7-59	OPD
Zaria	21	Male	FB 7-59	OPD
Zaria	23	female	FB 7-59	OPD
Zaria	57	Male	FB 7-59	OPD
Zaria	14	female	FB 7-59	OPD
Zaria	12	female	FB 7-59	OPD
Zaria	9	Male	FB 7-59	OPD
Zaria	22	Male	FB 7-59	OPD
Zaria	11	female	FB 7-59	OPD
Zaria	27	female	FB 7-59	OPD

Zaria	46	female	FB 7-59	OPD
Zaria	11	female	FB 7-59	OPD
Zaria	23	female	FB 7-59	OPD
Zaria	31	female	FB 7-59	OPD
Zaria	14	female	FB 7-59	OPD
Zaria	14	Male	FB 7-59	OPD
Zaria	12	female	FB 7-59	OPD
Zaria	21	female	FB 7-59	OPD
Zaria	12	Male	FB 7-59	OPD
Zaria	28	female	FB 7-59	OPD
Zaria	9	Male	FB 7-59	OPD
Zaria	8	Male	FB 7-59	OPD
Zaria	14	female	FB 7-59	OPD
Zaria	16	Male	FB 7-59	OPD
Zaria	15	Male	FB 7-59	OPD
Zaria	42	Male	FB 7-59	OPD
Zaria	21	female	FB 7-59	OPD
Zaria	10	Male	FB 7-59	OPD
Zaria	1	female	fb0-6	OPD
Zaria	39	Male	FB 7-59	OPD
Zaria	22	Male	FB 7-59	OPD
Zaria	28	Male	FB 7-59	OPD
Zaria	6	Male	fb0-6	OPD
Zaria	24	female	FB 7-59	OPD
Zaria	14	Male	FB 7-59	OPD
Zaria	8	Male	FB 7-59	OPD
Zaria	10	Male	FB 7-59	OPD
Zaria	15	female	FB 7-59	OPD
Zaria	7	female	FB 7-59	OPD
Zaria	7	Male	FB 7-59	OPD
Zaria	14	female	FB 7-59	OPD
Zaria	14	Male	FB 7-59	OPD
Zaria	14	female	FB 7-59	OPD
Zaria	17	Male	FB 7-59	OPD
Zaria	28	female	FB 7-59	OPD
Zaria	10	Male	FB 7-59	OPD
Zaria	15	Male	FB 7-59	OPD
Zaria	21	Male	FB 7-59	OPD
Zaria	9	Male	FB 7-59	OPD
Zaria	28	female	FB 7-59	OPD
Zaria	14	female	FB 7-59	OPD
Zaria	56	Male	FB 7-59	OPD
DRC	2	female	fever	OPD
DRC	5	female	>1 CSI	OPD
DRC	3	Male	fever	OPD
DRC	3	Male	fever	OPD
DRC	6	Male	fever	OPD
DRC	4	female	poor feeding	OPD
DRC	2	female	fever	OPD
DRC	1	Male	Hypo	OPD

DRC	2	Male	fever	OPD
DRC	1	Male	Hypo	OPD
DRC	2	female	fever	OPD
DRC	3	female	fever	OPD
DRC	2	female	>1 CSI	OPD
DRC	5	Male	fever	OPD
DRC	3	Male	fever	OPD
DRC	2	Male	>1 CSI	OPD
DRC	2	female	fever	OPD
DRC	5	female	fever	OPD
DRC	3	female	fever	OPD
DRC	6	Male	fever	OPD
DRC	1	Male	fever	OPD
DRC	1	Male	fever	OPD
DRC	1	Male	fever	OPD
DRC	3	Male	fever	OPD
DRC	2	female	fever	OPD
DRC	5	Male	fever	OPD
DRC	1	Male	fever	OPD
DRC	4	female	LCI	OPD
DRC	1	female	Hypo	OPD
DRC	1	female	fever	OPD
DRC	1	Male	LCI	OPD
DRC	1	Male	>1 CSI	OPD
DRC	3	Male	fever	OPD
DRC	1	female	fever	OPD
DRC	3	female	fever	OPD
DRC	5	female	fever	OPD
DRC	6	female	fever	OPD
DRC	2	Male	fever	OPD
DRC	3	Male	fever	OPD
DRC	1	Male	Hypo	OPD
DRC	2	Male	fever	OPD
DRC	5	Male	poor feeding	OPD
DRC	2	female	fever	OPD
DRC	2	female	fever	OPD
DRC	4	female	fever	OPD
DRC	5	Male	poor feeding	OPD
DRC	1	Male	>1 CSI	OPD
DRC	1	Male	movement on stimula	OPD
DRC	1	female	Hypo	OPD
DRC	2	Male	fever	OPD
DRC	1	Male	LCI	OPD
DRC	1	Male	>1 CSI	OPD
DRC	1	female	fever	OPD
DRC	3	female	fever	OPD
DRC	6	female	fever	OPD
DRC	2	Male	fever	OPD
DRC	3	female	fever	OPD
DRC	1	Male	LCI	OPD

DRC	2	female	fever	OPD
DRC	2	Male	fever	OPD
DRC	1	female	LCI	OPD
DRC	6	female	fever	OPD
DRC	6	Male	fever	OPD
DRC	2	Male	fever	OPD
DRC	4	Male	fever	OPD
DRC	2	female	fever	OPD
DRC	3	female	fever	OPD
DRC	3	female	>1 CSI	OPD
DRC	1	Male	Hypo	OPD
DRC	6	Male	fever	OPD
DRC	2	Male	fever	OPD
DRC	3	Male	fever	OPD
DRC	3	Male	fever	OPD
DRC	3	Male	fever	OPD
DRC	5	female	fever	OPD
DRC	1	Male	fever	OPD
DRC	2	female	poor feeding	OPD
DRC	1	Male	Hypo	OPD
DRC	4	female	fever	OPD
DRC	2	Male	fever	OPD
DRC	1	Male	>1 CSI	OPD
DRC	3	Male	fever	OPD
DRC	1	Male	poor feeding	OPD
DRC	2	female	fever	OPD
DRC	2	Male	fever	OPD
DRC	5	Male	>1 CSI	OPD
DRC	3	Male	fever	OPD
DRC	6	female	fever	OPD
DRC	3	Male	fever	OPD
DRC	2	Male	fever	OPD
DRC	3	Male	fever	OPD
DRC	2	female	Hypo	OPD
DRC	4	Male	LCI	OPD
DRC	3	Male	poor feeding	OPD
DRC	2	female	fever	OPD
DRC	6	Male	poor feeding	OPD
DRC	2	female	fever	OPD
DRC	2	Male	fever	OPD
DRC	3	female	fever	OPD
DRC	5	female	fever	OPD
DRC	6	Male	>1 CSI	OPD
DRC	4	female	fever	OPD
DRC	1	Male	fever	OPD
DRC	3	Male	poor feeding	OPD
DRC	1	Male	fever	OPD
DRC	2	Male	fever	OPD
DRC	1	Male	poor feeding	OPD
DRC	3	Male	Hypo	OPD

DRC	4	female	fever	OPD
DRC	5	Male	poor feeding	OPD
DRC	2	Male	fever	OPD
DRC	3	female	fever	OPD
DRC	1	female	poor feeding	OPD
DRC	1	Male	fever	OPD
DRC	1	Male	Hypo	OPD
DRC	1	Male	Hypo	OPD
DRC	3	female	fever	OPD
DRC	1	Male	Hypo	OPD
DRC	5	Male	fever	OPD
DRC	4	Male	Hypo	OPD
DRC	1	Male	Hypo	OPD
DRC	3	Male	fever	OPD
DRC	3	Male	Hypo	OPD
DRC	2	female	Hypo	OPD
DRC	2	female	Hypo	OPD
DRC	2	female	Hypo	OPD
DRC	4	female	fever	OPD
DRC	2	Male	>1 CSI	OPD
DRC	1	female	Hypo	OPD
DRC	1	Male	>1 CSI	OPD
DRC	5	female	LCI	OPD
DRC	2	Male	fever	OPD
DRC	2	Male	fever	OPD
DRC	2	Male	poor feeding	OPD
DRC	2	Male	poor feeding	OPD
DRC	3	female	fever	OPD
DRC	3	female	fever	OPD
DRC	1	Male	>1 CSI	OPD
DRC	1	Male	fever	OPD
DRC	2	female	>1 CSI	OPD
DRC	1	Male	poor feeding	OPD
DRC	1	female	>1 CSI	OPD
DRC	4	female	fever	OPD
DRC	2	Male	fever	OPD
DRC	3	Male	fever	OPD
DRC	4	Male	fever	OPD
DRC	3	Male	fever	OPD
DRC	2	female	fever	OPD
DRC	4	Male	>1 CSI	OPD
DRC	2	Male	>1 CSI	OPD
DRC	3	female	fever	OPD
DRC	5	Male	fever	OPD
DRC	5	Male	>1 CSI	OPD
DRC	3	Male	fever	OPD
DRC	3	female	fever	OPD
DRC	2	female	fever	OPD
DRC	2	female	>1 CSI	OPD
DRC	4	female	fever	OPD

DRC	2	female	fever	OPD
DRC	4	female	fever	OPD
DRC	4	Male	LCI	OPD
DRC	3	female	fever	OPD
DRC	3	Male	fever	OPD
DRC	3	Male	fever	OPD
DRC	2	female	fever	OPD
DRC	5	female	fever	OPD
DRC	2	Male	>1 CSI	OPD
DRC	2	female	fever	OPD
DRC	2	female	>1 CSI	OPD
DRC	3	female	Hypo	OPD
DRC	1	female	Hypo	OPD
DRC	3	female	>1 CSI	OPD
DRC	3	female	fever	OPD
DRC	5	Male	fever	OPD
DRC	2	female	fever	OPD
DRC	2	Male	fever	OPD
DRC	1	female	fever	OPD
DRC	3	Male	fever	OPD
DRC	1	Male	fever	OPD
DRC	1	Male	fever	OPD
DRC	1	female	>1 CSI	OPD
DRC	2	Male	fever	OPD
DRC	2	Male	fever	OPD
DRC	1	Male	Hypo	OPD
DRC	1	female	>1 CSI	OPD
DRC	2	Male	Hypo	OPD
DRC	1	female	Hypo	OPD
DRC	2	female	fever	OPD
DRC	1	female	Hypo	OPD
DRC	6	female	LCI	OPD
DRC	4	Male	fever	OPD
DRC	3	female	fever	OPD
DRC	2	female	fever	OPD
DRC	2	female	fever	OPD
DRC	2	female	fever	OPD
DRC	3	Male	fever	OPD
DRC	1	female	>1 CSI	OPD
DRC	1	Male	Hypo	OPD
DRC	11	Male	>1 CSI	OPD
DRC	21	female	LCI	OPD
DRC	34	female	fever	OPD
DRC	16	female	fever	OPD
DRC	21	female	LCI	OPD
DRC	21	female	LCI	OPD
DRC	12	female	movement on stimula	OPD
DRC	9	Male	LCI	OPD
DRC	7	female	fever	OPD
DRC	26	Male	LCI	OPD

DRC	7	female	fever	OPD
DRC	25	Male	fever	OPD
DRC	28	Male	>1 CSI	OPD
DRC	42	female	fever	OPD
DRC	15	female	LCI	OPD
DRC	14	Male	fever	OPD
DRC	28	Male	>1 CSI	OPD
DRC	7	female	LCI	OPD
DRC	25	female	fever	OPD
DRC	14	Male	>1 CSI	OPD
DRC	15	Male	LCI	OPD
DRC	58	female	>1 CSI	OPD
DRC	17	Male	fever	OPD
DRC	11	female	poor feeding	OPD
DRC	10	Male	fever	OPD
DRC	18	female	fever	OPD
DRC	16	Male	LCI	OPD
DRC	22	female	fever	OPD
DRC	7	female	fever	OPD
DRC	49	Male	fever	OPD
DRC	26	Male	LCI	OPD
DRC	47	Male	fever	OPD
DRC	54	female	fever	OPD
DRC	34	Male	fever	OPD
DRC	44	Male	LCI	OPD
DRC	53	female	fever	OPD
DRC	14	Male	fever	OPD
DRC	26	female	fever	OPD
DRC	54	female	fever	OPD
DRC	14	Male	LCI	OPD
DRC	8	female	fever	OPD
DRC	9	Male	fever	OPD
DRC	41	Male	fever	OPD
DRC	3	Male	fever	OPD
DRC	10	female	fever	OPD
DRC	9	Male	poor feeding	OPD
DRC	28	female	fever	OPD
DRC	19	Male	fever	OPD
DRC	17	female	fever	OPD
DRC	36	female	fever	OPD
DRC	10	female	LCI	OPD
DRC	11	female	fever	OPD
DRC	35	female	fever	OPD
DRC	5	female	LCI	OPD
DRC	12	Male	>1 CSI	OPD
DRC	55	female	>1 CSI	OPD
DRC	34	Male	LCI	OPD
DRC	50	female	fever	OPD
DRC	9	female	fever	OPD
DRC	28	female	fever	OPD

DRC	41	Male	LCI	OPD
DRC	23	female	LCI	OPD
DRC	44	Male	LCI	OPD
DRC	42	Male	fever	OPD
DRC	20	Male	>1 CSI	OPD
DRC	50	Male	LCI	OPD
DRC	46	Male	LCI	OPD
DRC	16	Male	fever	OPD
DRC	19	female	LCI	OPD
DRC	18	Male	fever	OPD
DRC	17	Male	LCI	OPD
DRC	8	Male	poor feeding	OPD
DRC	8	Male	fever	OPD
DRC	19	female	>1 CSI	OPD
DRC	48	Male	poor feeding	OPD
DRC	36	Male	fever	OPD
DRC	51	female	>1 CSI	OPD
DRC	15	Male	fever	OPD
DRC	10	female	poor feeding	OPD
DRC	7	female	fever	OPD
DRC	12	Male	fever	OPD
DRC	43	Male	LCI	OPD
DRC	27	Male	fever	OPD
DRC	10	female	fever	OPD
DRC	27	female	LCI	OPD
DRC	18	Male	fever	OPD
DRC	16	Male	>1 CSI	OPD
DRC	24	Male	LCI	OPD
DRC	43	Male	fever	OPD
DRC	7	female	convulsions	OPD
DRC	46	Male	LCI	OPD
DRC	39	Male	fever	OPD
DRC	27	female	LCI	OPD
DRC	35	female	LCI	OPD
DRC	7	female	Hypo	OPD
DRC	14	female	fever	OPD
DRC	10	female	poor feeding	OPD
DRC	29	Male	LCI	OPD
DRC	9	Male	fever	OPD
DRC	39	female	fever	OPD
DRC	48	Male	fever	OPD
DRC	51	female	LCI	OPD
DRC	21	female	fever	OPD
DRC	15	female	LCI	OPD
DRC	24	Male	>1 CSI	OPD
DRC	33	female	fever	OPD
DRC	14	Male	>1 CSI	OPD
DRC	21	Male	poor feeding	OPD
DRC	14	Male	fever	OPD
DRC	12	Male	poor feeding	OPD

DRC	10	Male	LCI	OPD
DRC	10	female	fever	OPD
DRC	11	Male	fever	OPD
DRC	15	female	LCI	OPD
DRC	59	female	fever	OPD
DRC	12	Male	fever	OPD
DRC	17	female	fever	OPD
DRC	46	female	fever	OPD
DRC	42	female	fever	OPD
DRC	27	Male	fever	OPD
DRC	29	Male	fever	OPD
DRC	42	female	LCI	OPD
DRC	13	Male	fever	OPD
DRC	32	Male	fever	OPD
DRC	21	female	fever	OPD
DRC	8	female	fever	OPD
DRC	53	female	fever	OPD
DRC	15	Male	LCI	OPD
DRC	47	Male	LCI	OPD
DRC	18	Male	LCI	OPD
DRC	10	female	fever	OPD
DRC	12	Male	LCI	OPD
DRC	8	Male	>1 CSI	OPD
DRC	30	female	fever	OPD
DRC	15	female	poor feeding	OPD
DRC	10	Male	LCI	OPD
DRC	8	Male	>1 CSI	OPD
DRC	10	Male	>1 CSI	OPD
DRC	12	female	LCI	OPD
DRC	14	female	fever	OPD
DRC	15	female	fever	OPD
DRC	50	female	poor feeding	OPD
DRC	31	female	fever	OPD
DRC	28	female	LCI	OPD
DRC	35	female	LCI	OPD
DRC	11	female	LCI	OPD
DRC	16	female	fever	OPD
DRC	6	female	fever	OPD
DRC	30	female	LCI	OPD
DRC	8	Male	LCI	OPD
DRC	32	Male	LCI	OPD
DRC	29	Male	>1 CSI	OPD
DRC	29	Male	fever	OPD
DRC	45	Male	>1 CSI	OPD
DRC	46	female	>1 CSI	OPD
DRC	35	Male	fever	OPD
DRC	9	Male	LCI	OPD
DRC	14	female	LCI	OPD
DRC	36	female	fever	OPD
DRC	10	Male	fever	OPD

DRC	4	Male	fever	OPD
DRC	19	female	fever	OPD
DRC	28	female	fever	OPD
DRC	14	Male	fever	OPD
DRC	8	Male	fever	OPD
DRC	8	Male	LCI	OPD
DRC	15	Male	fever	OPD
DRC	13	Male	fever	OPD
DRC	25	female	>1 CSI	OPD
DRC	23	Male	>1 CSI	OPD
DRC	24	female	fever	OPD
DRC	20	Male	fever	OPD
DRC	42	Male	fever	OPD
DRC	7	female	poor feeding	OPD
DRC	5	female	fever	OPD
DRC	39	female	fever	OPD
DRC	24	female	fever	OPD
DRC	16	female	>1 CSI	OPD
DRC	8	female	fever	OPD
DRC	59	female	fever	OPD
DRC	16	female	>1 CSI	OPD
DRC	49	Male	fever	OPD
DRC	7	female	fever	OPD
DRC	8	Male	>1 CSI	OPD
DRC	39	female	LCI	OPD
DRC	14	Male	fever	OPD
DRC	29	Male	LCI	OPD
DRC	16	female	LCI	OPD
DRC	8	female	fever	OPD
DRC	12	Male	LCI	OPD
DRC	13	female	LCI	OPD
DRC	21	female	LCI	OPD
DRC	42	Male	fever	OPD
DRC	21	female	LCI	OPD
DRC	53	female	fever	OPD
DRC	17	Male	LCI	OPD
DRC	51	Male	fever	OPD
DRC	3	female	LCI	OPD
DRC	32	female	LCI	OPD
DRC	9	Male	fever	OPD
DRC	9	Male	Hypo	OPD
DRC	19	female	fever	OPD
DRC	15	female	poor feeding	OPD
DRC	12	Male	LCI	OPD
DRC	6	female	>1 CSI	OPD
DRC	14	Male	LCI	OPD
DRC	3	Male	fever	OPD
DRC	14	Male	fever	OPD
DRC	1	Male	fever	OPD
DRC	26	female	fever	OPD

DRC	18	female	fever	OPD
DRC	1	Male	fever	OPD
DRC	16	female	fever	OPD
DRC	13	female	fever	OPD
DRC	7	female	Hypo	OPD
DRC	35	female	fever	OPD
DRC	12	Male	LCI	OPD
DRC	29	female	fever	OPD
DRC	56	female	>1 CSI	OPD
DRC	10	Male	fever	OPD
DRC	18	female	LCI	OPD
DRC	4	Male	fever	OPD
DRC	16	Male	LCI	OPD
DRC	58	female	>1 CSI	OPD
DRC	15	Male	Hypo	OPD
DRC	52	female	fever	OPD
DRC	32	Male	LCI	OPD
DRC	27	female	>1 CSI	OPD
DRC	23	female	>1 CSI	OPD
DRC	10	Male	>1 CSI	OPD
DRC	43	female	fever	OPD
DRC	33	female	fever	OPD
DRC	18	Male	LCI	OPD
DRC	20	Male	LCI	OPD
DRC	12	Male	>1 CSI	OPD
DRC	10	Male	fever	OPD
DRC	45	female	fever	OPD
DRC	27	Male	fever	OPD
DRC	40	Male	LCI	OPD
DRC	15	Male	poor feeding	OPD
DRC	10	female	LCI	OPD
DRC	16	Male	>1 CSI	OPD
DRC	9	Male	fever	OPD
DRC	17	Male	fever	OPD
DRC	15	Male	fever	OPD
DRC	21	female	fever	OPD
DRC	10	female	poor feeding	OPD
DRC	19	Male	LCI	OPD
DRC	33	female	fever	OPD
DRC	47	Male	fever	OPD
DRC	19	Male	fever	OPD
DRC	11	Male	fever	OPD
DRC	17	Male	LCI	OPD
DRC	12	Male	fever	OPD
DRC	30	female	fever	OPD
DRC	57	Male	>1 CSI	OPD
DRC	7	female	poor feeding	OPD
DRC	45	female	>1 CSI	OPD
DRC	56	female	fever	OPD
DRC	21	Male	LCI	OPD

DRC	35	Male	LCI	OPD
DRC	58	female	fever	OPD
DRC	1	Male	LCI	OPD
DRC	12	female	fever	OPD
DRC	13	female	fever	OPD
DRC	3	Male	fever	OPD
DRC	17	Male	>1 CSI	OPD
DRC	14	Male	LCI	OPD
DRC	15	Male	fever	OPD
DRC	1	Male	fever	OPD
DRC	16	Male	>1 CSI	OPD
DRC	18	Male	fever	OPD
DRC	14	Male	LCI	OPD
DRC	54	Male	LCI	OPD
DRC	49	female	fever	OPD
DRC	38	Male	LCI	OPD
DRC	21	female	fever	OPD
DRC	15	Male	fever	OPD
DRC	10	female	>1 CSI	OPD
DRC	38	Male	fever	OPD
DRC	14	Male	fever	OPD
DRC	24	female	LCI	OPD
DRC	51	Male	fever	OPD
DRC	15	female	fever	OPD
DRC	22	female	fever	OPD
DRC	15	female	LCI	OPD
DRC	12	Male	fever	OPD
DRC	12	female	fever	OPD
DRC	22	Male	fever	OPD
DRC	26	female	fever	OPD
DRC	20	female	fever	OPD
DRC	11	female	LCI	OPD
DRC	32	Male	fever	OPD
DRC	13	female	fever	OPD
DRC	46	Male	fever	OPD
DRC	7	female	fever	OPD
DRC	7	Male	poor feeding	OPD
DRC	53	Male	fever	OPD
DRC	30	female	LCI	OPD
DRC	15	female	fever	OPD
DRC	22	female	fever	OPD
DRC	5	female	fever	OPD
DRC	10	female	LCI	OPD
DRC	27	female	>1 CSI	OPD
DRC	28	female	LCI	OPD
DRC	28	Male	LCI	OPD
DRC	15	female	fever	OPD
DRC	31	Male	LCI	OPD
DRC	10	Male	LCI	OPD
DRC	11	female	LCI	OPD

DRC	2	female	fever	OPD
DRC	19	Male	fever	OPD
DRC	17	Male	fever	OPD
DRC	15	Male	fever	OPD
DRC	50	female	fever	OPD
DRC	3	female	fever	OPD
DRC	19	Male	poor feeding	OPD
DRC	29	Male	LCI	OPD
DRC	49	female	fever	OPD
DRC	12	female	LCI	OPD
DRC	7	Male	LCI	OPD
DRC	32	female	fever	OPD
DRC	7	female	fever	OPD
DRC	2	Male	fever	OPD
DRC	9	Male	fever	OPD
DRC	29	female	fever	OPD
DRC	11	Male	fever	OPD
DRC	17	female	>1 CSI	OPD
DRC	8	female	LCI	OPD
DRC	2	Male	fever	OPD
DRC	11	Male	poor feeding	OPD
DRC	20	Male	LCI	OPD
DRC	33	Male	fever	OPD
DRC	33	Male	fever	OPD
DRC	34	female	fever	OPD
DRC	14	Male	fever	OPD
DRC	41	female	fever	OPD
DRC	18	female	LCI	OPD
DRC	20	female	fever	OPD
DRC	8	Male	LCI	OPD
DRC	29	female	>1 CSI	OPD
DRC	22	female	fever	OPD
DRC	14	Male	LCI	OPD
DRC	46	Male	fever	OPD
DRC	14	Male	fever	OPD
DRC	46	female	fever	OPD
DRC	11	Male	LCI	OPD
DRC	20	female	>1 CSI	OPD
DRC	23	Male	LCI	OPD
DRC	9	Male	fever	OPD
DRC	46	female	fever	OPD
DRC	37	female	fever	OPD
DRC	24	Male	fever	OPD
DRC	45	Male	fever	OPD
DRC	12	female	fever	OPD
DRC	27	female	fever	OPD
DRC	41	Male	poor feeding	OPD
DRC	6	Male	fever	OPD
DRC	20	female	>1 CSI	OPD
DRC	3	Male	fever	OPD

DRC	18	female	LCI	OPD
DRC	21	Male	fever	OPD
DRC	37	female	fever	OPD
DRC	8	Male	fever	OPD
DRC	10	Male	LCI	OPD
DRC	22	female	LCI	OPD
DRC	19	female	LCI	OPD
DRC	48	Male	>1 CSI	OPD
DRC	13	Male	fever	OPD
DRC	20	Male	>1 CSI	OPD
DRC	8	female	fever	OPD
DRC	10	female	fever	OPD
DRC	9	Male	fever	OPD
DRC	9	Male	fever	OPD
DRC	8	Male	fever	OPD
DRC	9	female	fever	OPD
DRC	2	Male	fb0-6	OPD
DRC	1	Male	fb0-6	OPD
DRC	2	female	fb0-6	OPD
DRC	1	Male	fb0-6	OPD
DRC	3	Male	fb0-6	OPD
DRC	2	Male	fb0-6	OPD
DRC	3	Male	fb0-6	OPD
DRC	1	female	fb0-6	OPD
DRC	5	Male	fb0-6	OPD
DRC	1	Male	fb0-6	OPD
DRC	1	Male	fb0-6	OPD
DRC	6	Male	fb0-6	OPD
DRC	3	Male	fb0-6	OPD
DRC	4	Male	fb0-6	OPD
DRC	1	Male	fb0-6	OPD
DRC	2	Male	fb0-6	OPD
DRC	3	female	fb0-6	OPD
DRC	1	Male	fb0-6	OPD
DRC	1	Male	fb0-6	OPD
DRC	2	Male	fb0-6	OPD
DRC	4	female	fb0-6	OPD
DRC	4	Male	fb0-6	OPD
DRC	2	Male	fb0-6	OPD
DRC	2	Male	fb0-6	OPD
DRC	2	Male	fb0-6	OPD
DRC	4	Male	fb0-6	OPD
DRC	3	female	fb0-6	OPD
DRC	1	Male	fb0-6	OPD
DRC	1	Male	fb0-6	OPD
DRC	6	female	fb0-6	OPD
DRC	2	Male	fb0-6	OPD
DRC	6	Male	fb0-6	OPD
DRC	2	Male	fb0-6	OPD
DRC	6	female	fb0-6	OPD

DRC	5 Male	fb0-6	OPD
DRC	1 Male	fb0-6	OPD
DRC	1 female	fb0-6	OPD
DRC	3 Male	fb0-6	OPD
DRC	2 female	fb0-6	OPD
DRC	1 female	fb0-6	OPD
DRC	3 Male	fb0-6	OPD
DRC	3 female	fb0-6	OPD
DRC	3 Male	fb0-6	OPD
DRC	6 Male	fb0-6	OPD
DRC	4 Male	fb0-6	OPD
DRC	2 Male	fb0-6	OPD
DRC	1 Male	fb0-6	OPD
DRC	3 female	fb0-6	OPD
DRC	3 Male	fb0-6	OPD
DRC	4 Male	fb0-6	OPD
DRC	2 Male	fb0-6	OPD
DRC	3 Male	fb0-6	OPD
DRC	1 Male	fb0-6	OPD
DRC	4 Male	fb0-6	OPD
DRC	4 Male	fb0-6	OPD
DRC	4 female	fb0-6	OPD
DRC	4 female	fb0-6	OPD
DRC	3 female	fb0-6	OPD
DRC	3 Male	fb0-6	OPD
DRC	1 female	fb0-6	OPD
DRC	6 female	fb0-6	OPD
DRC	2 female	fb0-6	OPD
DRC	5 female	fb0-6	OPD
DRC	2 female	fb0-6	OPD
DRC	2 Male	fb0-6	OPD
DRC	5 female	fb0-6	OPD
DRC	3 female	fb0-6	OPD
DRC	2 Male	fb0-6	OPD
DRC	1 Male	fb0-6	OPD
DRC	2 Male	fb0-6	OPD
DRC	2 Male	fb0-6	OPD
DRC	3 female	fb0-6	OPD
DRC	1 female	fb0-6	OPD
DRC	4 female	fb0-6	OPD
DRC	2 female	fb0-6	OPD
DRC	3 female	fb0-6	OPD
DRC	3 female	fb0-6	OPD
DRC	1 Male	fb0-6	OPD
DRC	2 Male	fb0-6	OPD
DRC	2 female	fb0-6	OPD
DRC	1 Male	fb0-6	OPD
DRC	3 Male	fb0-6	OPD
DRC	1 Male	fb0-6	OPD
DRC	2 Male	fb0-6	OPD

DRC	2	Male	fb0-6	OPD
DRC	2	female	fb0-6	OPD
DRC	1	Male	fb0-6	OPD
DRC	1	Male	fb0-6	OPD
DRC	3	Male	fb0-6	OPD
DRC	3	female	fb0-6	OPD
DRC	2	female	fb0-6	OPD
DRC	2	female	fb0-6	OPD
DRC	6	Male	fb0-6	OPD
DRC	2	Male	fb0-6	OPD
DRC	1	Male	fb0-6	OPD
DRC	2	female	fb0-6	OPD
DRC	4	Male	fb0-6	OPD
DRC	2	female	fb0-6	OPD
DRC	3	female	fb0-6	OPD
DRC	2	Male	fb0-6	OPD
DRC	5	Male	fb0-6	OPD
DRC	1	Male	fb0-6	OPD
DRC	4	female	fb0-6	OPD
DRC	4	Male	fb0-6	OPD
DRC	2	Male	fb0-6	OPD
DRC	3	Male	fb0-6	OPD
DRC	2	female	fb0-6	OPD
DRC	3	Male	fb0-6	OPD
DRC	2	female	fb0-6	OPD
DRC	1	Male	fb0-6	OPD
DRC	2	female	fb0-6	OPD
DRC	4	Male	fb0-6	OPD
DRC	6	Male	fb0-6	OPD
DRC	3	Male	fb0-6	OPD
DRC	4	female	fb0-6	OPD
DRC	3	Male	fb0-6	OPD
DRC	3	Male	fb0-6	OPD
DRC	1	Male	fb0-6	OPD
DRC	5	Male	fb0-6	OPD
DRC	3	Male	fb0-6	OPD
DRC	2	female	fb0-6	OPD
DRC	2	female	fb0-6	OPD
DRC	3	Male	fb0-6	OPD
DRC	6	Male	fb0-6	OPD
DRC	3	female	fb0-6	OPD
DRC	3	Male	fb0-6	OPD
DRC	3	Male	fb0-6	OPD
DRC	3	female	fb0-6	OPD
DRC	3	female	fb0-6	OPD
DRC	3	Male	fb0-6	OPD
DRC	2	Male	fb0-6	OPD
DRC	3	Male	fb0-6	OPD
DRC	2	female	fb0-6	OPD
DRC	3	Male	fb0-6	OPD

DRC	2	Male	fb0-6	OPD
DRC	2	Male	fb0-6	OPD
DRC	3	Male	fb0-6	OPD
DRC	5	female	fb0-6	OPD
DRC	1	Male	fb0-6	OPD
DRC	5	Male	fb0-6	OPD
DRC	1	female	fb0-6	OPD
DRC	3	Male	fb0-6	OPD
DRC	1	female	fb0-6	OPD
DRC	3	Male	fb0-6	OPD
DRC	2	Male	fb0-6	OPD
DRC	3	Male	fb0-6	OPD
DRC	2	female	fb0-6	OPD
DRC	4	Male	fb0-6	OPD
DRC	1	female	fb0-6	OPD
DRC	2	female	fb0-6	OPD
DRC	3	Male	fb0-6	OPD
DRC	2	female	fb0-6	OPD
DRC	5	female	fb0-6	OPD
DRC	6	Male	fb0-6	OPD
DRC	2	Male	fb0-6	OPD
DRC	1	Male	fb0-6	OPD
DRC	1	Male	fb0-6	OPD
DRC	14	female	FB 7-59	OPD
DRC	29	female	FB 7-59	OPD
DRC	18	Male	FB 7-59	OPD
DRC	15	Male	FB 7-59	OPD
DRC	13	Male	FB 7-59	OPD
DRC	7	female	FB 7-59	OPD
DRC	14	female	FB 7-59	OPD
DRC	19	Male	FB 7-59	OPD
DRC	8	female	FB 7-59	OPD
DRC	9	female	FB 7-59	OPD
DRC	44	female	FB 7-59	OPD
DRC	14	female	FB 7-59	OPD
DRC	15	Male	FB 7-59	OPD
DRC	18	female	FB 7-59	OPD
DRC	19	female	FB 7-59	OPD
DRC	18	Male	FB 7-59	OPD
DRC	19	Male	FB 7-59	OPD
DRC	34	female	FB 7-59	OPD
DRC	23	female	FB 7-59	OPD
DRC	22	Male	FB 7-59	OPD
DRC	24	Male	FB 7-59	OPD
DRC	8	Male	FB 7-59	OPD
DRC	10	Male	FB 7-59	OPD
DRC	10	female	FB 7-59	OPD
DRC	14	female	FB 7-59	OPD
DRC	1	Male	fb0-6	OPD
DRC	8	female	FB 7-59	OPD

DRC	34	female	FB 7-59	OPD
DRC	10	female	FB 7-59	OPD
DRC	13	female	FB 7-59	OPD
DRC	18	female	FB 7-59	OPD
DRC	23	Male	FB 7-59	OPD
DRC	18	female	FB 7-59	OPD
DRC	20	Male	FB 7-59	OPD
DRC	17	female	FB 7-59	OPD
DRC	29	female	FB 7-59	OPD
DRC	17	Male	FB 7-59	OPD
DRC	15	female	FB 7-59	OPD
DRC	11	Male	FB 7-59	OPD
DRC	18	female	FB 7-59	OPD
DRC	14	Male	FB 7-59	OPD
DRC	23	Male	FB 7-59	OPD
DRC	7	Male	FB 7-59	OPD
DRC	23	female	FB 7-59	OPD
DRC	7	Male	FB 7-59	OPD
DRC	17	female	FB 7-59	OPD
DRC	15	Male	FB 7-59	OPD
DRC	9	Male	FB 7-59	OPD
DRC	15	Male	FB 7-59	OPD
DRC	15	female	FB 7-59	OPD
DRC	22	female	FB 7-59	OPD
DRC	43	female	FB 7-59	OPD
DRC	14	Male	FB 7-59	OPD
DRC	2	Male	fb0-6	OPD
DRC	14	Male	FB 7-59	OPD
DRC	9	Male	FB 7-59	OPD
DRC	15	female	FB 7-59	OPD
DRC	8	Male	FB 7-59	OPD
DRC	25	Male	FB 7-59	OPD
DRC	8	Male	FB 7-59	OPD
DRC	35	Male	FB 7-59	OPD
DRC	21	Male	FB 7-59	OPD
DRC	14	female	FB 7-59	OPD
DRC	16	Male	FB 7-59	OPD
DRC	15	Male	FB 7-59	OPD
DRC	23	Male	FB 7-59	OPD
DRC	15	female	FB 7-59	OPD
DRC	14	Male	FB 7-59	OPD
DRC	21	Male	FB 7-59	OPD
DRC	15	Male	FB 7-59	OPD
DRC	19	female	FB 7-59	OPD
DRC	12	female	FB 7-59	OPD
DRC	11	female	FB 7-59	OPD
DRC	14	female	FB 7-59	OPD
DRC	37	Male	FB 7-59	OPD
DRC	12	female	FB 7-59	OPD
DRC	9	Male	FB 7-59	OPD

DRC	21	Male	FB 7-59	OPD
DRC	3	Male	fb0-6	OPD
DRC	38	Male	FB 7-59	OPD
DRC	46	female	FB 7-59	OPD
DRC	21	female	FB 7-59	OPD
DRC	14	female	FB 7-59	OPD
DRC	16	female	FB 7-59	OPD
DRC	10	female	FB 7-59	OPD
DRC	13	female	FB 7-59	OPD
DRC	22	Male	FB 7-59	OPD
DRC	13	female	FB 7-59	OPD
DRC	13	Male	FB 7-59	OPD
DRC	6	Male	fb0-6	OPD
DRC	14	Male	FB 7-59	OPD
DRC	12	female	FB 7-59	OPD
DRC	11	Male	FB 7-59	OPD
DRC	12	female	FB 7-59	OPD
DRC	22	Male	FB 7-59	OPD
DRC	3	Male	fb0-6	OPD
DRC	10	female	FB 7-59	OPD
DRC	9	Male	FB 7-59	OPD
DRC	13	Male	FB 7-59	OPD
DRC	7	female	FB 7-59	OPD
DRC	17	female	FB 7-59	OPD
DRC	10	Male	FB 7-59	OPD
DRC	22	female	FB 7-59	OPD
DRC	37	Male	FB 7-59	OPD
DRC	27	female	FB 7-59	OPD
DRC	18	Male	FB 7-59	OPD
DRC	17	female	FB 7-59	OPD
DRC	14	female	FB 7-59	OPD
DRC	20	Male	FB 7-59	OPD
DRC	8	Male	FB 7-59	OPD
DRC	10	female	FB 7-59	OPD
DRC	18	Male	FB 7-59	OPD
DRC	12	Male	FB 7-59	OPD
DRC	23	Male	FB 7-59	OPD
DRC	16	female	FB 7-59	OPD
DRC	16	Male	FB 7-59	OPD
DRC	21	Male	FB 7-59	OPD
DRC	12	female	FB 7-59	OPD
DRC	8	Male	FB 7-59	OPD
DRC	22	female	FB 7-59	OPD
DRC	14	female	FB 7-59	OPD
DRC	21	female	FB 7-59	OPD
DRC	47	female	FB 7-59	OPD
DRC	12	Male	FB 7-59	OPD
DRC	8	Male	FB 7-59	OPD
DRC	13	Male	FB 7-59	OPD
DRC	9	female	FB 7-59	OPD

DRC	16	Male	FB 7-59	OPD
DRC	25	Male	FB 7-59	OPD
DRC	6	female	fb0-6	OPD
DRC	8	Male	FB 7-59	OPD
DRC	17	female	FB 7-59	OPD
DRC	44	Male	FB 7-59	OPD
DRC	10	female	FB 7-59	OPD
DRC	7	Male	FB 7-59	OPD
DRC	12	Male	FB 7-59	OPD
DRC	23	female	FB 7-59	OPD
DRC	27	Male	FB 7-59	OPD
DRC	10	Male	FB 7-59	OPD
DRC	9	Male	FB 7-59	OPD
DRC	9	Male	FB 7-59	OPD
DRC	35	female	FB 7-59	OPD
DRC	14	Male	FB 7-59	OPD
DRC	15	female	FB 7-59	OPD
DRC	3	Male	fb0-6	OPD
DRC	7	female	FB 7-59	OPD
DRC	7	female	FB 7-59	OPD
DRC	14	Male	FB 7-59	OPD
DRC	21	female	FB 7-59	OPD
DRC	37	Male	FB 7-59	OPD
DRC	14	Male	FB 7-59	OPD
DRC	10	female	FB 7-59	OPD
DRC	13	female	FB 7-59	OPD
DRC	6	Male	fb0-6	OPD
DRC	18	female	FB 7-59	OPD
DRC	21	Male	FB 7-59	OPD
DRC	13	female	FB 7-59	OPD
DRC	44	female	FB 7-59	OPD
DRC	13	Male	FB 7-59	OPD
DRC	30	female	FB 7-59	OPD
DRC	24	female	FB 7-59	OPD
DRC	21	female	FB 7-59	OPD
DRC	28	Male	FB 7-59	OPD
DRC	25	Male	FB 7-59	OPD
DRC	8	Male	FB 7-59	OPD
DRC	45	Male	FB 7-59	OPD
DRC	14	Male	FB 7-59	OPD
DRC	17	Male	FB 7-59	OPD
DRC	14	Male	FB 7-59	OPD
DRC	12	female	FB 7-59	OPD
DRC	13	female	FB 7-59	OPD
DRC	15	Male	FB 7-59	OPD
DRC	26	female	FB 7-59	OPD
DRC	13	female	FB 7-59	OPD
DRC	13	Male	FB 7-59	OPD
DRC	22	female	FB 7-59	OPD
DRC	15	Male	FB 7-59	OPD

DRC	8	Male	FB 7-59	OPD
DRC	6	Male	fb0-6	OPD
DRC	7	female	FB 7-59	OPD
DRC	35	Male	FB 7-59	OPD
DRC	7	female	FB 7-59	OPD
DRC	18	female	FB 7-59	OPD
DRC	9	Male	FB 7-59	OPD
DRC	20	female	FB 7-59	OPD
DRC	44	Male	FB 7-59	OPD
DRC	13	Male	FB 7-59	OPD
DRC	8	Male	FB 7-59	OPD
DRC	21	female	FB 7-59	OPD
DRC	28	female	FB 7-59	OPD
DRC	7	Male	FB 7-59	OPD
DRC	18	Male	FB 7-59	OPD
DRC	8	Male	FB 7-59	OPD
DRC	13	female	FB 7-59	OPD
DRC	18	female	FB 7-59	OPD
DRC	11	Male	FB 7-59	OPD
DRC	46	female	FB 7-59	OPD
DRC	28	Male	FB 7-59	OPD
DRC	9	Male	FB 7-59	OPD
DRC	24	Male	FB 7-59	OPD
DRC	16	Male	FB 7-59	OPD
DRC	8	Male	FB 7-59	OPD
DRC	22	Male	FB 7-59	OPD
DRC	9	Male	FB 7-59	OPD
DRC	14	Male	FB 7-59	OPD
DRC	19	female	FB 7-59	OPD
DRC	14	Male	FB 7-59	OPD
DRC	49	female	FB 7-59	OPD
DRC	25	Male	FB 7-59	OPD
DRC	15	female	FB 7-59	OPD
DRC	15	Male	FB 7-59	OPD
DRC	9	Male	FB 7-59	OPD
DRC	12	female	FB 7-59	OPD
DRC	18	female	FB 7-59	OPD
DRC	2	Male	fb0-6	OPD
DRC	7	Male	FB 7-59	OPD
DRC	22	Male	FB 7-59	OPD
DRC	13	female	FB 7-59	OPD
DRC	14	Male	FB 7-59	OPD
DRC	21	Male	FB 7-59	OPD
DRC	26	Male	FB 7-59	OPD
DRC	28	female	FB 7-59	OPD
DRC	7	Male	FB 7-59	OPD
DRC	10	female	FB 7-59	OPD
DRC	15	female	FB 7-59	OPD
DRC	3	Male	fb0-6	OPD
DRC	27	Male	FB 7-59	OPD

DRC	8	Male	FB 7-59	OPD
DRC	29	Male	FB 7-59	OPD
DRC	22	female	FB 7-59	OPD
DRC	11	Male	FB 7-59	OPD
DRC	7	Male	FB 7-59	OPD
DRC	9	female	FB 7-59	OPD
DRC	10	female	FB 7-59	OPD
DRC	7	Male	FB 7-59	OPD
DRC	25	female	FB 7-59	OPD
DRC	23	female	FB 7-59	OPD
DRC	14	female	FB 7-59	OPD
DRC	34	female	FB 7-59	OPD
DRC	14	female	FB 7-59	OPD
DRC	12	female	FB 7-59	OPD
DRC	10	Male	FB 7-59	OPD
DRC	8	female	FB 7-59	OPD
DRC	4	female	fb0-6	OPD
DRC	16	female	FB 7-59	OPD
DRC	29	female	FB 7-59	OPD
DRC	9	Male	FB 7-59	OPD
DRC	28	Male	FB 7-59	OPD
DRC	31	Male	FB 7-59	OPD
DRC	21	Male	FB 7-59	OPD
DRC	15	Male	FB 7-59	OPD
DRC	21	Male	FB 7-59	OPD
DRC	17	female	FB 7-59	OPD
DRC	25	Male	FB 7-59	OPD
DRC	17	female	FB 7-59	OPD
DRC	12	female	FB 7-59	OPD
DRC	12	Male	FB 7-59	OPD
DRC	10	Male	FB 7-59	OPD
DRC	34	Male	FB 7-59	OPD
DRC	7	female	FB 7-59	OPD
DRC	15	female	FB 7-59	OPD
DRC	17	Male	FB 7-59	OPD
DRC	21	female	FB 7-59	OPD
DRC	8	female	FB 7-59	OPD
DRC	10	Male	FB 7-59	OPD
DRC	14	Male	FB 7-59	OPD
DRC	22	Male	FB 7-59	OPD
DRC	5	Male	fb0-6	OPD
DRC	14	Male	FB 7-59	OPD
DRC	15	female	FB 7-59	OPD
DRC	12	female	FB 7-59	OPD
DRC	44	female	FB 7-59	OPD
DRC	21	female	FB 7-59	OPD
DRC	21	female	FB 7-59	OPD
DRC	40	Male	FB 7-59	OPD
Kenya	24	Male	fever	OPD
Ibadan	3	Male	fb0-6	Hospital

Ibadan	59	female	poor feeding	Hospital
Ibadan	9	female	FB 7-59	Hospital
Ibadan	6	female	fb0-6	Hospital
Kenya	6	Male	fb0-6	OPD
Zaria	6	Male	convulsions	Hospital
Ile Ife	3	Male	fb0-6	OPD
Ibadan	13	female	FB 7-59	Hospital
Ibadan	6	female	fb0-6	Hospital
Zaria	10	Male	FB 7-59	Hospital
Ibadan	15	female	>1 CSI	Hospital
Ibadan	14	Male	FB 7-59	Hospital
Zaria	1	Male	fb0-6	Hospital
Zaria	8	female	>1 CSI	OPD
Ile Ife	2	female	fb0-6	Hospital
DRC	2	Male	>1 CSI	Hospital
Kenya	10	female	FB 7-59	OPD
Zaria	1	Male	fb0-6	Hospital
Zaria	7	Male	FB 7-59	Hospital
Zaria	5	Male	fb0-6	Hospital
Ibadan	4	female	fb0-6	Hospital
Zaria	16	female	FB 7-59	Hospital
Zaria	1	Male	fb0-6	Hospital
Zaria	12	female	Hypo	Hospital
DRC	5	female	any CI	OPD
Ile Ife	2	Male	>1 CSI	Hospital
Zaria	12	female	FB 7-59	Hospital
Ile Ife	3	female	fever	Hospital
Zaria	3	Male	convulsions	OPD
DRC	6	Male	fb0-6	OPD
DRC	2	Male	Hypo	Hospital
Ibadan	2	female	fb0-6	Hospital
Kenya	12	Male	FB 7-59	Hospital
Ibadan	2	Male	fb0-6	Hospital
Kenya	4	female	fb0-6	OPD
Kenya	36	Male	FB 7-59	OPD
Ibadan	13	Male	convulsions	Hospital
DRC	2	Male	fb0-6	OPD
Kenya	17	female	FB 7-59	OPD
DRC	8	Male	no movement	OPD
Ibadan	1	female	convulsions	OPD
Zaria	21	Male	FB 7-59	Hospital
Zaria	13	female	FB 7-59	Hospital
DRC	17	Male	FB 7-59	Hospital
Ibadan	24	Male	LCI	Hospital
DRC	5	female	fb0-6	OPD
Zaria	1	Male	fb0-6	Hospital
Zaria	42	Male	FB 7-59	OPD
Ibadan	33	Male	FB 7-59	OPD
Zaria	3	Male	fb0-6	Hospital
Zaria	1	Male	fb0-6	Hospital

Zaria	3	Male	fb0-6	Hospital
Ibadan	11	female	FB 7-59	Hospital
Zaria	1	Male	fb0-6	Hospital
DRC	6	Male	not feeding	OPD
DRC	3	Male	any CI	Hospital
Zaria	1	Male	fb0-6	Hospital
Ile Ife	6	female	fb0-6	OPD
Zaria	36	female	fever	OPD
Kenya	6	female	fb0-6	OPD
Ibadan	6	Male	fb0-6	Hospital
Zaria	8	female	FB 7-59	Hospital
Ibadan	3	Male	convulsions	Hospital
DRC	13	Male	not feeding	OPD
Ibadan	2	Male	fb0-6	Hospital
Zaria	21	Male	FB 7-59	Hospital
Zaria	2	female	fb0-6	Hospital
Zaria	9	female	FB 7-59	Hospital
Ile Ife	1	female	fb0-6	Hospital
Ibadan	12	female	FB 7-59	Hospital
Ibadan	16	Male	FB 7-59	Hospital
DRC	2	Male	fb0-6	OPD
DRC	1	Male	fb0-6	Hospital
Zaria	8	female	FB 7-59	Hospital
Zaria	28	Male	FB 7-59	Hospital
Zaria	11	Male	FB 7-59	Hospital
Kenya	11	Male	FB 7-59	OPD
Ibadan	14	female	FB 7-59	Hospital
DRC	2	Male	fb0-6	OPD
Zaria	1	Male	convulsions	Hospital
Zaria	28	female	FB 7-59	Hospital
Zaria	1	female	fb0-6	Hospital
Ibadan	52	Male	FB 7-59	Hospital
Zaria	6	female	>1 CSI	Hospital
Zaria	24	female	FB 7-59	Hospital
DRC	37	female	FB 7-59	Hospital
DRC	10	Male	not feeding	OPD
Kenya	1	female	>1 CSI	Hospital
Zaria	1	female	>1 CSI	Hospital
Zaria	7	female	FB 7-59	Hospital
Zaria	2	female	convulsions	Hospital
Ibadan	5	female	fb0-6	Hospital
DRC	2	female	convulsions	OPD
Zaria	2	Male	fb0-6	Hospital
DRC	1	female	any CI	OPD
Kenya	36	Male	FB 7-59	OPD
Zaria	24	female	FB 7-59	Hospital
Zaria	28	female	FB 7-59	Hospital
Zaria	4	female	fb0-6	OPD
Kenya	11	female	not feeding	OPD
Zaria	3	female	Hypo	OPD

Ibadan	20	Male	FB 7-59	Hospital
Ibadan	14	Male	FB 7-59	Hospital
Zaria	35	female	FB 7-59	Hospital
Kenya	5	female	fb0-6	OPD
DRC	2	Male	fb0-6	OPD
Kenya	1	Male	fb0-6	OPD
DRC	6	female	convulsions	OPD
Zaria	21	female	FB 7-59	Hospital
DRC	16	Male	convulsions	OPD
Kenya	21	female	>1 CSI	Hospital
Zaria	12	female	FB 7-59	Hospital
Kenya	42	Male	FB 7-59	Hospital
Ibadan	26	Male	convulsions	Hospital
Zaria	49	Male	FB 7-59	Hospital
DRC	6	female	fb0-6	OPD
DRC	3	female	fb0-6	Hospital
Kenya	1	Male	Hypo	OPD
Kenya	2	Male	fb0-6	OPD
Ibadan	5	Male	Hypo	OPD
Zaria	14	female	FB 7-59	Hospital
Zaria	3	female	fb0-6	Hospital
Zaria	28	female	FB 7-59	Hospital
Zaria	7	female	no movement	OPD
Zaria	8	female	FB 7-59	Hospital
Ibadan	5	Male	convulsions	OPD
Zaria	19	Male	FB 7-59	Hospital
Ibadan	1	female	fb0-6	Hospital
Kenya	22	Male	FB 7-59	OPD
DRC	4	Male	not feeding	OPD
Zaria	16	Male	FB 7-59	Hospital
Ibadan	6	Male	fb0-6	Hospital
Kenya	5	Male	not feeding	Hospital
Zaria	20	female	FB 7-59	Hospital
Zaria	22	Male	FB 7-59	Hospital
Zaria	4	Male	fever	OPD
Kenya	23	Male	FB 7-59	OPD
Zaria	22	Male	FB 7-59	Hospital
Ibadan	27	female	FB 7-59	OPD
DRC	2	Male	fb0-6	Hospital
DRC	6	Male	fb0-6	Hospital
Kenya	37	female	not feeding	OPD
Ibadan	2	Male	fever	Hospital
Zaria	1	Male	fb0-6	Hospital
DRC	30	Male	FB 7-59	OPD
DRC	6	female	fb0-6	OPD
DRC	2	Male	convulsions	OPD
Kenya	2	female	fb0-6	OPD
DRC	4	female	any CI	Hospital
DRC	1	Male	LCI	OPD
Zaria	1	Male	convulsions	OPD

Zaria	6	Male	fb0-6	Hospital
Kenya	7	Male	FB 7-59	OPD
Zaria	42	Male	Hypo	OPD
Ibadan	1	female	fb0-6	Hospital
DRC	26	female	FB 7-59	OPD
Ibadan	22	Male	FB 7-59	Hospital
Kenya	8	female	fever	Hospital
Zaria	6	female	fb0-6	Hospital
Ibadan	9	Male	FB 7-59	Hospital
Zaria	16	female	FB 7-59	Hospital
Zaria	16	female	FB 7-59	Hospital
DRC	1	Male	fb0-6	OPD
Zaria	4	Male	fb0-6	Hospital
Zaria	2	female	Hypo	Hospital
Ibadan	49	female	poor feeding	Hospital
DRC	3	female	not feeding	Hospital
Ibadan	6	Male	fb0-6	Hospital
DRC	3	female	fb0-6	OPD
Zaria	1	female	convulsions	OPD
Zaria	3	female	convulsions	Hospital
Ibadan	22	female	FB 7-59	Hospital
Ibadan	26	female	convulsions	Hospital
Ile Ife	2	female	any CI	OPD
Zaria	14	Male	FB 7-59	Hospital
Zaria	7	female	FB 7-59	Hospital
Ibadan	2	Male	fb0-6	Hospital
Ile Ife	2	Male	fb0-6	Hospital
DRC	1	female	fb0-6	Hospital
Zaria	15	Male	FB 7-59	Hospital
Zaria	23	female	FB 7-59	Hospital
Zaria	9	Male	FB 7-59	Hospital
Zaria	20	female	fever	Hospital
Zaria	1	Male	fb0-6	Hospital
Kenya	6	Male	fb0-6	OPD
DRC	37	Male	any CI	Hospital
Zaria	14	female	FB 7-59	Hospital
Ibadan	9	female	FB 7-59	Hospital
Zaria	2	Male	fb0-6	Hospital
Zaria	2	female	>1 CSI	Hospital
Kenya	12	Male	not feeding	OPD
Ibadan	6	Male	fb0-6	Hospital
DRC	17	female	FB 7-59	OPD
Ibadan	5	female	fb0-6	Hospital
Zaria	29	female	FB 7-59	Hospital
DRC	15	Male	FB 7-59	OPD
Ibadan	19	female	FB 7-59	Hospital
Ibadan	43	Male	convulsions	Hospital
DRC	2	Male	>1 CSI	OPD
DRC	7	Male	>1 CSI	OPD
Ile Ife	2	Male	convulsions	OPD

Zaria	10	Male	FB 7-59	Hospital
Zaria	28	female	FB 7-59	Hospital
Zaria	7	female	FB 7-59	Hospital
Kenya	4	Male	any CI	Hospital
DRC	1	Male	fb0-6	OPD
Zaria	7	Male	FB 7-59	Hospital
Zaria	3	female	fb0-6	OPD
DRC	15	female	FB 7-59	Hospital
Ibadan	1	Male	>1 CSI	Hospital
DRC	1	Male	Hypo	OPD
Ibadan	19	Male	FB 7-59	Hospital
DRC	43	female	convulsions	OPD
Zaria	16	Male	FB 7-59	Hospital
Ibadan	3	female	fb0-6	Hospital
DRC	9	Male	not feeding	OPD
DRC	2	female	convulsions	Hospital
Zaria	7	female	FB 7-59	Hospital
Zaria	9	female	FB 7-59	Hospital
DRC	6	female	fb0-6	OPD
Ibadan	13	Male	convulsions	Hospital
DRC	38	Male	FB 7-59	Hospital
Zaria	26	female	FB 7-59	Hospital
Kenya	6	female	fb0-6	OPD
Kenya	7	female	>1 CSI	OPD
Ibadan	1	Male	convulsions	Hospital
Zaria	4	Male	>1 CSI	Hospital
Kenya	14	female	FB 7-59	OPD
Ibadan	1	Male	fb0-6	Hospital
Ibadan	2	Male	fb0-6	Hospital
Kenya	1	Male	no movement	OPD
Zaria	3	Male	fb0-6	Hospital
Ibadan	6	female	fb0-6	Hospital
Zaria	14	Male	FB 7-59	Hospital
Ibadan	3	Male	fb0-6	OPD
Zaria	17	female	FB 7-59	Hospital
Zaria	1	Male	Hypo	OPD
Zaria	8	female	FB 7-59	Hospital
Zaria	16	Male	FB 7-59	Hospital
Ibadan	6	Male	fever	Hospital
Zaria	14	female	FB 7-59	Hospital
DRC	23	Male	poor feeding	OPD
Zaria	10	Male	FB 7-59	Hospital
Kenya	39	female	convulsions	Hospital
Zaria	6	Male	fb0-6	Hospital
Ibadan	1	female	fb0-6	Hospital
DRC	1	female	Hypo	Hospital
Zaria	24	Male	FB 7-59	Hospital
Kenya	9	Male	poor feeding	OPD
Zaria	14	female	FB 7-59	Hospital
Zaria	6	Male	fb0-6	Hospital

Zaria	9	Male	FB 7-59	Hospital
Zaria	1	Male	fb0-6	Hospital
Zaria	5	Male	fb0-6	Hospital
Ibadan	1	female	fb0-6	Hospital
Zaria	2	female	fb0-6	Hospital
Ile Ife	3	Male	not feeding	OPD
Ibadan	8	Male	FB 7-59	Hospital
Zaria	3	Male	fb0-6	Hospital
DRC	12	Male	not feeding	OPD
Ile Ife	1	Male	fb0-6	Hospital
Zaria	32	female	FB 7-59	Hospital
Zaria	2	Male	Hypo	OPD
Ibadan	16	female	FB 7-59	Hospital
Kenya	57	female	FB 7-59	Hospital
Zaria	9	female	FB 7-59	Hospital
DRC	1	female	not feeding	OPD
DRC	7	Male	not feeding	Hospital
Zaria	2	female	fb0-6	Hospital
Zaria	5	Male	fb0-6	Hospital
Zaria	1	female	Hypo	OPD
Ile Ife	21	female	LCI	Hospital
DRC	1	female	Hypo	OPD
DRC	4	Male	movement on stimula	OPD
Ibadan	6	female	fb0-6	Hospital
DRC	1	Male	fb0-6	Hospital
DRC	5	female	fb0-6	OPD
DRC	15	Male	FB 7-59	OPD
Zaria	21	Male	FB 7-59	Hospital
Kenya	3	female	Hypo	OPD
Ile Ife	21	female	FB 7-59	OPD
Zaria	17	female	FB 7-59	Hospital
Ile Ife	1	Male	fb0-6	OPD
Ibadan	15	Male	FB 7-59	Hospital
Kenya	57	Male	FB 7-59	OPD
DRC	1	Male	no movement	OPD
DRC	6	female	fb0-6	OPD
Zaria	18	Male	fever	Hospital
Ile Ife	4	female	fb0-6	Hospital
Kenya	6	Male	fb0-6	OPD
DRC	2	female	fb0-6	OPD
Zaria	1	female	fb0-6	Hospital
DRC	2	female	convulsions	OPD
DRC	27	female	convulsions	Hospital
Ibadan	15	female	convulsions	Hospital
Ibadan	13	female	FB 7-59	Hospital
Ile Ife	7	Male	FB 7-59	OPD
Zaria	10	female	fever	OPD
DRC	15	female	FB 7-59	OPD
DRC	44	female	FB 7-59	OPD
Ibadan	6	female	fb0-6	Hospital

Zaria	1	Male	fb0-6	Hospital
Zaria	7	female	FB 7-59	Hospital
Ibadan	31	Male	FB 7-59	Hospital
Zaria	21	Male	FB 7-59	OPD
Zaria	2	Male	fb0-6	Hospital
Ile Ife	20	Male	LCI	OPD
Ibadan	3	female	fb0-6	Hospital
Zaria	14	Male	FB 7-59	Hospital
Ile Ife	1	Male	fb0-6	OPD
Kenya	23	Male	FB 7-59	OPD
Ile Ife	15	Male	LCI	Hospital
DRC	13	Male	FB 7-59	Hospital
Zaria	1	Male	fb0-6	Hospital
Kenya	11	Male	Hypo	OPD
Zaria	1	female	Hypo	OPD
Zaria	5	female	fb0-6	Hospital
Zaria	3	Male	fb0-6	OPD
Zaria	20	female	FB 7-59	Hospital
Ile Ife	2	Male	>1 CSI	OPD
Zaria	17	Male	FB 7-59	Hospital
DRC	6	female	fb0-6	OPD
Ibadan	1	female	convulsions	Hospital
Ibadan	6	Male	fb0-6	Hospital
Zaria	7	Male	FB 7-59	Hospital
Ile Ife	6	Male	fb0-6	Hospital
Zaria	1	female	fb0-6	OPD
Zaria	11	female	FB 7-59	Hospital
Zaria	28	Male	FB 7-59	Hospital
Zaria	28	female	FB 7-59	Hospital
Zaria	9	Male	FB 7-59	Hospital
Zaria	1	female	fb0-6	OPD
Zaria	7	Male	FB 7-59	OPD
Zaria	7	Male	FB 7-59	Hospital
Zaria	1	Male	fb0-6	Hospital
Ibadan	6	Male	fb0-6	Hospital
Zaria	9	Male	FB 7-59	Hospital
DRC	11	Male	>1 CSI	OPD
Ibadan	48	Male	fever	Hospital
DRC	4	female	Hypo	Hospital
Ile Ife	2	female	LCI	Hospital
Zaria	2	Male	fb0-6	Hospital
Zaria	2	Male	fb0-6	Hospital
DRC	2	female	Hypo	OPD
DRC	1	female	convulsions	OPD
Zaria	1	female	fb0-6	OPD
Zaria	21	female	FB 7-59	Hospital
Ile Ife	2	female	fever	Hospital
Zaria	3	Male	fb0-6	Hospital
Ibadan	49	female	FB 7-59	Hospital
Ibadan	6	Male	fb0-6	Hospital

DRC	29	Male	not feeding	OPD
Zaria	3	Male	fb0-6	Hospital
Zaria	1	Male	fb0-6	OPD
DRC	12	female	no movement	Hospital
Zaria	12	female	FB 7-59	Hospital
Zaria	28	female	FB 7-59	Hospital
Zaria	1	Male	fb0-6	Hospital
Zaria	15	Male	FB 7-59	Hospital
Ibadan	9	Male	FB 7-59	Hospital
Kenya	50	female	FB 7-59	OPD
Zaria	5	Male	fb0-6	Hospital
Zaria	14	Male	poor feeding	Hospital
Zaria	9	Male	FB 7-59	Hospital
Zaria	1	female	fb0-6	Hospital
DRC	6	Male	fb0-6	OPD
DRC	42	Male	poor feeding	OPD
Ibadan	32	female	FB 7-59	Hospital
Ibadan	7	Male	FB 7-59	Hospital
Zaria	9	female	FB 7-59	Hospital
Zaria	7	Male	FB 7-59	Hospital
Ibadan	6	Male	fb0-6	Hospital
DRC	25	female	no movement	OPD
Ibadan	12	Male	FB 7-59	Hospital
Zaria	9	female	FB 7-59	Hospital
Zaria	9	Male	FB 7-59	Hospital
Zaria	40	Male	FB 7-59	Hospital
Zaria	10	Male	FB 7-59	Hospital
DRC	2	female	convulsions	OPD
Kenya	6	female	fb0-6	Hospital
Ibadan	14	female	LCI	Hospital
Zaria	19	Male	FB 7-59	Hospital
DRC	51	Male	convulsions	Hospital
DRC	1	female	fb0-6	OPD
DRC	2	female	LCI	Hospital
Zaria	3	Male	fb0-6	Hospital
Zaria	4	Male	fb0-6	Hospital
Ile Ife	3	Male	convulsions	OPD
Zaria	7	Male	FB 7-59	OPD
Ile Ife	25	female	FB 7-59	OPD
Zaria	14	Male	FB 7-59	Hospital
Zaria	6	Male	Hypo	Hospital
Ile Ife	21	Male	FB 7-59	OPD
Zaria	1	Male	fb0-6	Hospital
Ile Ife	1	Male	fb0-6	Hospital
Zaria	1	female	no movement	OPD
Zaria	14	Male	FB 7-59	Hospital
Ibadan	2	Male	fb0-6	Hospital
Zaria	4	female	fb0-6	Hospital
Ibadan	10	female	convulsions	Hospital
Zaria	4	Male	convulsions	OPD

Zaria	4	Male	fb0-6	Hospital
Zaria	14	female	FB 7-59	Hospital
DRC	1	Male	Hypo	OPD
DRC	4	Male	>1 CSI	OPD
Zaria	36	female	FB 7-59	Hospital
Zaria	12	Male	FB 7-59	Hospital
DRC	2	female	Hypo	Hospital
Zaria	10	Male	>1 CSI	Hospital
DRC	3	Male	not feeding	Hospital
Zaria	14	female	FB 7-59	Hospital
DRC	6	female	convulsions	OPD
Kenya	56	female	poor feeding	OPD
Ibadan	2	Male	fb0-6	Hospital
Kenya	1	Male	Hypo	Hospital
DRC	2	female	fb0-6	OPD
Zaria	2	female	fb0-6	OPD
Ile Ife	2	female	fb0-6	Hospital
Ibadan	19	Male	FB 7-59	Hospital
Ibadan	2	Male	fb0-6	Hospital
DRC	2	Male	>1 CSI	Hospital
Kenya	15	Male	FB 7-59	Hospital
Ibadan	59	Male	FB 7-59	Hospital
DRC	5	female	poor feeding	Hospital
Zaria	17	female	FB 7-59	OPD
Ibadan	1	Male	fb0-6	Hospital
Ibadan	1	female	fever	Hospital
Zaria	9	Male	FB 7-59	Hospital
Zaria	2	female	convulsions	Hospital
Zaria	15	female	FB 7-59	Hospital
DRC	21	Male	any CI	Hospital
Zaria	1	female	fb0-6	Hospital
Zaria	14	female	FB 7-59	OPD
Ile Ife	6	Male	fb0-6	Hospital
Ibadan	42	female	LCI	Hospital
DRC	2	female	convulsions	Hospital
Zaria	6	female	fb0-6	Hospital
Zaria	15	Male	FB 7-59	Hospital
Zaria	2	Male	convulsions	OPD
Ile Ife	2	Male	fb0-6	Hospital
Kenya	6	Male	fb0-6	OPD
Ibadan	2	female	movement on stimula	Hospital
Zaria	3	Male	fb0-6	Hospital
Zaria	16	Male	FB 7-59	Hospital
Zaria	4	female	convulsions	Hospital
Kenya	56	Male	fever	Hospital
Zaria	35	Male	FB 7-59	Hospital
Ibadan	12	female	FB 7-59	Hospital
Ibadan	6	Male	fb0-6	Hospital
DRC	22	female	convulsions	OPD
Zaria	21	Male	FB 7-59	Hospital

DRC	1	female	fb0-6	OPD
Ibadan	3	female	fever	Hospital
DRC	16	Male	FB 7-59	OPD
DRC	10	Male	Hypo	OPD
Ibadan	3	Male	fb0-6	Hospital
Zaria	1	Male	fb0-6	Hospital
Zaria	11	Male	FB 7-59	OPD
Zaria	13	female	FB 7-59	Hospital
Zaria	1	female	fb0-6	Hospital
Kenya	11	female	FB 7-59	OPD
Kenya	48	female	Hypo	OPD
Zaria	12	Male	FB 7-59	Hospital
DRC	6	Male	fb0-6	OPD
Kenya	6	female	>1 CSI	OPD
Kenya	3	female	>1 CSI	OPD
Zaria	54	female	FB 7-59	Hospital
DRC	4	Male	fb0-6	OPD
Zaria	14	Male	FB 7-59	Hospital
DRC	32	female	any CI	Hospital
Zaria	7	Male	FB 7-59	OPD
Zaria	3	Male	fb0-6	OPD
Zaria	14	female	FB 7-59	Hospital
Zaria	2	Male	fb0-6	OPD
DRC	11	Male	>1 CSI	OPD
Kenya	37	Male	FB 7-59	OPD
Kenya	6	female	fb0-6	OPD
Zaria	2	Male	convulsions	OPD
Zaria	1	Male	fb0-6	Hospital
Zaria	10	female	FB 7-59	Hospital
Zaria	9	Male	FB 7-59	Hospital
Zaria	1	Male	convulsions	OPD
Kenya	28	Male	LCI	OPD
DRC	6	Male	fb0-6	OPD
Kenya	51	Male	convulsions	OPD
Zaria	3	female	no movement	OPD
Ibadan	29	Male	LCI	Hospital
Kenya	42	female	poor feeding	OPD
Ibadan	6	Male	fb0-6	Hospital
Kenya	34	female	not feeding	Hospital
Ibadan	4	Male	convulsions	Hospital
Ibadan	1	female	fb0-6	Hospital
Zaria	1	Male	fb0-6	OPD
Zaria	50	Male	fever	Hospital
DRC	30	Male	convulsions	OPD
Zaria	22	Male	convulsions	Hospital
Ibadan	4	female	fb0-6	Hospital
DRC	6	Male	fb0-6	OPD
Ibadan	2	Male	fever	OPD
Zaria	8	Male	FB 7-59	Hospital
Ile Ife	2	female	fever	Hospital

Kenya	2	Male	fb0-6	Hospital
Kenya	13	female	poor feeding	OPD
Zaria	3	Male	fb0-6	Hospital
Ibadan	2	Male	fb0-6	Hospital
DRC	1	female	>1 CSI	Hospital
DRC	1	female	>1 CSI	OPD
Kenya	1	female	Hypo	OPD
Zaria	7	Male	FB 7-59	Hospital
Ile Ife	4	Male	LCI	OPD
Zaria	21	Male	FB 7-59	Hospital
Kenya	1	Male	fb0-6	OPD
Zaria	26	female	FB 7-59	Hospital
DRC	2	female	not feeding	Hospital
Ibadan	28	Male	convulsions	Hospital
Zaria	2	Male	fb0-6	Hospital
Ibadan	2	Male	convulsions	Hospital
Zaria	1	Male	fb0-6	Hospital
Kenya	9	Male	FB 7-59	OPD
Zaria	1	Male	fb0-6	Hospital
Ile Ife	1	female	fever	Hospital
Zaria	20	female	LCI	OPD
Ibadan	1	Male	any CI	Hospital
DRC	2	female	convulsions	Hospital
Ibadan	4	Male	fb0-6	Hospital
Zaria	7	Male	FB 7-59	Hospital
Zaria	27	Male	FB 7-59	Hospital
DRC	1	Male	fb0-6	Hospital
Ibadan	10	Male	FB 7-59	Hospital
Zaria	11	Male	FB 7-59	Hospital
DRC	2	female	fb0-6	OPD
DRC	6	Male	any CI	OPD
Zaria	14	female	FB 7-59	Hospital
DRC	21	Male	FB 7-59	OPD
DRC	1	Male	fb0-6	Hospital
Zaria	32	Male	FB 7-59	Hospital
Zaria	2	Male	convulsions	OPD
Kenya	6	Male	fb0-6	Hospital
Kenya	10	female	FB 7-59	Hospital
DRC	1	female	>1 CSI	OPD
Kenya	17	female	FB 7-59	OPD
Kenya	5	Male	fb0-6	Hospital
DRC	15	female	>1 CSI	OPD
Kenya	1	female	fb0-6	OPD
Ibadan	1	Male	convulsions	Hospital
Ile Ife	1	female	fb0-6	OPD
Ibadan	11	Male	FB 7-59	Hospital
DRC	1	Male	fb0-6	OPD
Zaria	14	female	FB 7-59	OPD
Zaria	6	Male	fb0-6	Hospital
DRC	22	Male	convulsions	OPD

Zaria	6	Male	fb0-6	Hospital
DRC	5	female	>1 CSI	OPD
Kenya	9	Male	FB 7-59	OPD
Ile Ife	6	Male	fb0-6	Hospital
Zaria	9	Male	FB 7-59	Hospital
Zaria	21	female	FB 7-59	Hospital
Zaria	9	Male	FB 7-59	Hospital
DRC	1	Male	fb0-6	OPD
Zaria	44	Male	FB 7-59	Hospital
DRC	1	female	Hypo	Hospital
Zaria	14	female	FB 7-59	Hospital
DRC	5	female	poor feeding	Hospital
Zaria	1	Male	fb0-6	OPD
Ibadan	1	Male	fb0-6	Hospital
Zaria	9	female	FB 7-59	Hospital
DRC	7	female	>1 CSI	OPD
Ibadan	1	Male	fb0-6	Hospital
DRC	3	Male	fb0-6	OPD
DRC	4	Male	fb0-6	OPD
Zaria	6	female	fb0-6	Hospital
Zaria	5	female	fb0-6	Hospital
DRC	2	female	not feeding	Hospital
Ile Ife	7	Male	fever	OPD
DRC	3	Male	fb0-6	OPD
Ile Ife	1	Male	fever	Hospital
Zaria	4	female	fb0-6	Hospital
Zaria	6	Male	Hypo	OPD
DRC	23	female	FB 7-59	OPD
DRC	1	Male	fb0-6	OPD
DRC	27	female	not feeding	OPD
DRC	3	Male	any CI	OPD
DRC	1	Male	fb0-6	OPD
Ibadan	20	female	convulsions	Hospital
Zaria	8	Male	FB 7-59	Hospital
Ile Ife	6	Male	fb0-6	Hospital
Zaria	54	female	FB 7-59	Hospital
Zaria	5	Male	fb0-6	Hospital
Kenya	11	Male	FB 7-59	OPD
Ibadan	3	Male	LCI	Hospital
Ibadan	20	Male	FB 7-59	Hospital
DRC	2	Male	Hypo	OPD
DRC	8	Male	any CI	OPD
Zaria	14	Male	FB 7-59	Hospital
Ibadan	37	Male	fever	Hospital
Zaria	26	Male	poor feeding	OPD
Zaria	21	Male	FB 7-59	Hospital
Ibadan	51	female	convulsions	Hospital
Kenya	6	female	fb0-6	OPD
DRC	15	female	FB 7-59	OPD
Zaria	1	Male	fb0-6	Hospital

Kenya	8	Male	convulsions	OPD
DRC	22	Male	not feeding	OPD
Ibadan	23	female	convulsions	Hospital
DRC	6	female	fb0-6	OPD
DRC	19	Male	convulsions	Hospital
Ile Ife	4	female	fb0-6	Hospital
Ile Ife	1	Male	fb0-6	OPD
DRC	6	female	fb0-6	OPD
Kenya	22	Male	convulsions	OPD
Kenya	16	Male	FB 7-59	OPD
DRC	22	female	FB 7-59	OPD
DRC	6	female	convulsions	Hospital
Ibadan	1	female	Hypo	Hospital
DRC	19	Male	fever	OPD
Ibadan	1	Male	>1 CSI	OPD
Ibadan	19	Male	FB 7-59	Hospital
DRC	1	female	fb0-6	OPD
DRC	1	Male	fb0-6	Hospital
Zaria	28	Male	FB 7-59	Hospital
Zaria	10	Male	FB 7-59	Hospital
DRC	3	Male	convulsions	Hospital
Ile Ife	5	Male	fb0-6	Hospital
Zaria	8	Male	FB 7-59	Hospital
Zaria	1	female	fb0-6	Hospital
Ile Ife	2	Male	fb0-6	OPD
DRC	1	Male	fb0-6	Hospital
DRC	6	female	fb0-6	OPD
Zaria	7	female	FB 7-59	Hospital
Zaria	7	female	FB 7-59	Hospital
DRC	1	Male	Hypo	Hospital
Kenya	21	Male	FB 7-59	OPD
DRC	1	Male	fb0-6	OPD
DRC	6	female	fb0-6	Hospital
DRC	3	Male	not feeding	Hospital
Ile Ife	3	Male	fb0-6	Hospital
Kenya	6	female	fb0-6	OPD
DRC	6	female	fb0-6	OPD
Ibadan	11	Male	FB 7-59	OPD
Zaria	14	female	FB 7-59	Hospital
Zaria	1	Male	convulsions	Hospital
DRC	14	Male	not feeding	Hospital
Zaria	22	Male	FB 7-59	Hospital
Zaria	3	female	fb0-6	Hospital
Zaria	1	female	fb0-6	OPD
Ile Ife	5	Male	fb0-6	Hospital
Kenya	2	Male	fb0-6	OPD
Zaria	14	Male	FB 7-59	Hospital
DRC	29	female	not feeding	Hospital
DRC	3	Male	fb0-6	OPD
DRC	2	female	any CI	Hospital

DRC	2	female	any CI	Hospital
Zaria	1	Male	fb0-6	Hospital
DRC	6	female	fb0-6	OPD
DRC	1	female	fb0-6	Hospital
DRC	6	female	fb0-6	OPD
DRC	7	female	any CI	Hospital
Ile Ife	18	female	convulsions	OPD
Ile Ife	5	Male	fb0-6	OPD
Zaria	17	Male	FB 7-59	Hospital
DRC	6	Male	fb0-6	OPD
Zaria	3	Male	fb0-6	Hospital
DRC	18	female	convulsions	OPD
DRC	14	female	fever	OPD
DRC	6	Male	fb0-6	Hospital
Ile Ife	16	female	FB 7-59	OPD
Kenya	6	female	fb0-6	OPD
DRC	4	Male	fb0-6	OPD
DRC	6	Male	no movement	Hospital
Zaria	28	Male	FB 7-59	Hospital
Zaria	7	Male	FB 7-59	Hospital
Kenya	10	Male	Hypo	OPD
DRC	2	Male	fb0-6	OPD
Ile Ife	3	female	fever	OPD
Ibadan	26	female	FB 7-59	Hospital
DRC	33	Male	FB 7-59	OPD
DRC	3	female	fb0-6	OPD
Zaria	17	Male	FB 7-59	Hospital
DRC	2	Male	convulsions	Hospital
Zaria	3	female	Hypo	Hospital
DRC	7	female	any CI	OPD
Ibadan	1	female	fb0-6	Hospital
DRC	6	Male	convulsions	OPD
Ile Ife	2	female	LCI	Hospital
Zaria	18	Male	FB 7-59	Hospital
Zaria	6	Male	fb0-6	Hospital
Zaria	28	female	FB 7-59	Hospital
Zaria	13	Male	FB 7-59	Hospital
Zaria	20	Male	FB 7-59	Hospital
Zaria	25	Male	FB 7-59	OPD
Ibadan	50	Male	fever	Hospital
Ibadan	7	Male	FB 7-59	Hospital
Ile Ife	2	female	fb0-6	Hospital
Zaria	28	female	FB 7-59	Hospital
Zaria	8	Male	FB 7-59	Hospital
DRC	1	female	convulsions	OPD
Zaria	17	female	FB 7-59	OPD
DRC	3	Male	fb0-6	OPD
Kenya	30	Male	FB 7-59	OPD
Zaria	23	female	FB 7-59	Hospital
Zaria	7	female	FB 7-59	Hospital

Zaria	55	Male	FB 7-59	Hospital
Zaria	22	Male	convulsions	Hospital
Zaria	12	Male	not feeding	OPD
DRC	1	female	convulsions	OPD
Zaria	1	Male	fb0-6	Hospital
DRC	7	Male	no movement	OPD
Zaria	8	female	FB 7-59	Hospital
Kenya	42	Male	FB 7-59	Hospital
Zaria	42	female	FB 7-59	Hospital
Ile Ife	1	female	fb0-6	OPD
Zaria	7	female	FB 7-59	Hospital
Zaria	23	Male	FB 7-59	Hospital
Zaria	1	female	Hypo	OPD
DRC	2	Male	Hypo	Hospital
Zaria	13	female	FB 7-59	Hospital
Zaria	7	Male	FB 7-59	OPD
Zaria	22	Male	convulsions	Hospital
DRC	31	Male	fever	OPD
Zaria	1	Male	convulsions	OPD
DRC	20	Male	FB 7-59	OPD
DRC	2	female	>1 CSI	OPD
DRC	43	female	not feeding	OPD
Zaria	3	Male	fb0-6	Hospital
Kenya	1	female	Hypo	OPD
Ibadan	3	Male	fb0-6	Hospital
Zaria	33	female	FB 7-59	Hospital
Zaria	33	Male	FB 7-59	Hospital
DRC	6	Male	convulsions	OPD
Zaria	37	Male	FB 7-59	Hospital
Zaria	8	female	FB 7-59	OPD
Zaria	21	Male	FB 7-59	Hospital
DRC	1	female	not feeding	Hospital
Zaria	3	female	fb0-6	Hospital
DRC	11	female	FB 7-59	OPD
Zaria	3	Male	fb0-6	Hospital
Zaria	7	female	FB 7-59	OPD
DRC	7	Male	poor feeding	OPD
DRC	3	Male	fb0-6	Hospital
Kenya	46	female	fever	Hospital
Zaria	8	Male	FB 7-59	OPD
Zaria	9	Male	FB 7-59	OPD
Ibadan	46	female	convulsions	Hospital
DRC	59	female	fever	OPD
DRC	6	Male	fb0-6	OPD
DRC	29	Male	FB 7-59	OPD
DRC	3	female	not feeding	Hospital
Zaria	14	Male	FB 7-59	Hospital
Zaria	19	Male	FB 7-59	Hospital
DRC	2	Male	fb0-6	OPD
Ile Ife	5	Male	fb0-6	Hospital

Kenya	25	Male	not feeding	OPD
Zaria	3	female	fb0-6	Hospital
Ibadan	4	Male	any CI	OPD
Zaria	1	Male	fb0-6	Hospital
Zaria	9	Male	LCI	OPD
DRC	6	female	fb0-6	OPD
Kenya	3	female	fb0-6	Hospital
Ile Ife	7	Male	FB 7-59	OPD
Ibadan	6	female	fb0-6	Hospital
Zaria	1	Male	fb0-6	Hospital
Kenya	1	Male	>1 CSI	OPD
DRC	10	Male	fever	OPD
Kenya	40	Male	FB 7-59	OPD
DRC	26	Male	FB 7-59	OPD
DRC	6	female	fb0-6	OPD
Zaria	21	Male	FB 7-59	Hospital
DRC	9	female	Hypo	Hospital
Zaria	45	female	FB 7-59	Hospital
Zaria	31	Male	FB 7-59	Hospital
Zaria	23	female	FB 7-59	Hospital
Ibadan	3	Male	fever	Hospital
DRC	24	Male	fever	OPD
Zaria	37	Male	FB 7-59	Hospital
Zaria	18	Male	FB 7-59	Hospital
Zaria	16	Male	FB 7-59	Hospital
Ibadan	35	Male	convulsions	Hospital
Zaria	14	female	FB 7-59	Hospital
Zaria	28	Male	FB 7-59	Hospital
Kenya	37	Male	fever	Hospital
Kenya	7	female	FB 7-59	Hospital
Kenya	6	female	fb0-6	OPD
DRC	1	Male	fb0-6	Hospital
Zaria	7	Male	FB 7-59	Hospital
Zaria	1	Male	Hypo	OPD
DRC	38	Male	fever	Hospital
Zaria	1	Male	fb0-6	Hospital
DRC	42	Male	fever	OPD
Ibadan	1	Male	fb0-6	Hospital
Zaria	4	female	fb0-6	Hospital
Zaria	9	female	FB 7-59	Hospital
Kenya	21	female	FB 7-59	OPD
Zaria	5	Male	fb0-6	Hospital
Zaria	8	Male	FB 7-59	Hospital
Zaria	1	Male	fb0-6	Hospital
Kenya	6	Male	fb0-6	OPD
Zaria	21	female	FB 7-59	OPD
Zaria	1	Male	fb0-6	OPD
Zaria	7	female	FB 7-59	Hospital
Zaria	43	female	FB 7-59	Hospital
Zaria	16	female	FB 7-59	Hospital

Zaria	27	Male	FB 7-59	Hospital
DRC	59	Male	LCI	OPD
Zaria	53	Male	FB 7-59	Hospital
Zaria	1	Male	fb0-6	OPD
Zaria	15	female	FB 7-59	Hospital
Zaria	16	Male	FB 7-59	Hospital
Ibadan	1	Male	convulsions	Hospital
Zaria	14	Male	>1 CSI	OPD
Ile Ife	9	Male	FB 7-59	OPD
DRC	48	female	FB 7-59	OPD
DRC	57	female	FB 7-59	OPD
Zaria	2	Male	fb0-6	Hospital
Zaria	11	Male	FB 7-59	Hospital
DRC	43	female	not feeding	OPD
Zaria	21	Male	FB 7-59	OPD
Ibadan	21	female	FB 7-59	Hospital
Kenya	20	Male	FB 7-59	Hospital
Zaria	24	Male	FB 7-59	Hospital
Zaria	34	Male	FB 7-59	Hospital
Zaria	9	Male	FB 7-59	Hospital
Zaria	28	Male	FB 7-59	Hospital
Zaria	7	Male	FB 7-59	Hospital
DRC	1	female	no movement	Hospital
Zaria	6	Male	fb0-6	OPD
Zaria	16	Male	FB 7-59	Hospital
DRC	1	Male	Hypo	Hospital
Zaria	16	female	FB 7-59	Hospital
Zaria	11	female	FB 7-59	Hospital
Zaria	59	Male	FB 7-59	Hospital
Zaria	32	Male	FB 7-59	Hospital
Zaria	4	Male	fb0-6	Hospital
Kenya	1	Male	Hypo	OPD
Zaria	1	Male	convulsions	Hospital
Zaria	22	Male	FB 7-59	Hospital
Zaria	28	Male	FB 7-59	Hospital
Zaria	14	female	FB 7-59	Hospital
DRC	1	Male	>1 CSI	OPD
Zaria	5	Male	fb0-6	Hospital
Ibadan	4	Male	fb0-6	Hospital
Zaria	21	female	FB 7-59	Hospital
Zaria	1	Male	fb0-6	Hospital
Ibadan	42	female	FB 7-59	Hospital
Zaria	7	Male	FB 7-59	Hospital
Zaria	8	Male	FB 7-59	Hospital
Ile Ife	6	female	fb0-6	Hospital
Zaria	1	Male	Hypo	OPD
Zaria	15	Male	FB 7-59	Hospital
Ile Ife	17	female	FB 7-59	OPD
Zaria	22	female	FB 7-59	Hospital
Zaria	7	Male	FB 7-59	Hospital

Zaria	1	Male	fb0-6	Hospital
Zaria	3	female	fb0-6	Hospital
Zaria	1	female	Hypo	Hospital
Ibadan	42	female	FB 7-59	Hospital
Zaria	14	female	FB 7-59	Hospital
Zaria	21	Male	FB 7-59	Hospital
Ibadan	29	Male	FB 7-59	Hospital
DRC	6	Male	fb0-6	OPD
Kenya	39	Male	LCI	Hospital
Kenya	44	Male	fever	OPD
Zaria	3	Male	fb0-6	Hospital
Zaria	6	Male	fb0-6	Hospital
Zaria	2	Male	fb0-6	Hospital
DRC	6	female	fb0-6	Hospital
Zaria	1	female	fb0-6	OPD
Zaria	3	female	fb0-6	Hospital
Zaria	25	Male	FB 7-59	Hospital
Zaria	1	Male	fb0-6	Hospital
DRC	54	Male	fever	OPD
Zaria	7	Male	FB 7-59	Hospital
Zaria	15	female	FB 7-59	Hospital
Zaria	35	Male	FB 7-59	Hospital
DRC	1	Male	>1 CSI	OPD
Zaria	1	Male	fever	OPD
Zaria	3	Male	any CI	OPD
Zaria	11	Male	FB 7-59	Hospital
DRC	6	Male	fb0-6	OPD
Zaria	12	female	FB 7-59	Hospital
Zaria	17	Male	FB 7-59	Hospital
Zaria	22	female	FB 7-59	OPD
Ibadan	1	female	fb0-6	Hospital
Zaria	7	Male	FB 7-59	Hospital
Zaria	3	Male	fb0-6	OPD
DRC	1	Male	>1 CSI	Hospital
Zaria	15	female	FB 7-59	Hospital
Ibadan	2	Male	convulsions	Hospital
Kenya	50	female	FB 7-59	OPD
Zaria	21	female	FB 7-59	Hospital
Ibadan	1	female	fb0-6	Hospital
Zaria	1	female	fb0-6	Hospital
Zaria	8	Male	FB 7-59	Hospital
Ibadan	25	female	FB 7-59	Hospital
Kenya	36	Male	FB 7-59	OPD
Ibadan	6	Male	fb0-6	OPD
Ibadan	1	female	fb0-6	OPD
DRC	3	Male	convulsions	OPD
DRC	1	female	convulsions	OPD
Ibadan	5	female	fb0-6	Hospital
Zaria	7	Male	FB 7-59	Hospital
Zaria	8	female	convulsions	Hospital

Zaria	1	female	fb0-6	Hospital
Ibadan	31	female	FB 7-59	Hospital
Ibadan	1	Male	convulsions	Hospital
Zaria	5	Male	fb0-6	Hospital
DRC	2	Male	fb0-6	Hospital
DRC	1	Male	fb0-6	OPD
Zaria	3	female	fb0-6	Hospital
Ibadan	34	Male	FB 7-59	Hospital
Ibadan	28	female	FB 7-59	Hospital
Ibadan	3	female	fb0-6	OPD
Ibadan	8	female	FB 7-59	Hospital
Zaria	8	female	FB 7-59	Hospital
Ibadan	6	Male	fb0-6	OPD
Ibadan	42	Male	>1 CSI	Hospital
Ile Ife	8	female	FB 7-59	OPD
Zaria	2	female	fb0-6	Hospital
Ibadan	18	female	FB 7-59	Hospital
Ibadan	2	Male	fb0-6	Hospital
Kenya	8	Male	FB 7-59	OPD
Zaria	1	Male	fb0-6	Hospital
Zaria	9	female	FB 7-59	Hospital
Zaria	3	Male	fb0-6	Hospital
Ibadan	6	female	fb0-6	Hospital
Ibadan	6	female	fb0-6	Hospital
Ibadan	6	female	fb0-6	Hospital
Ile Ife	16	female	FB 7-59	OPD
Zaria	14	Male	FB 7-59	Hospital
Zaria	9	female	FB 7-59	Hospital
Ibadan	16	female	FB 7-59	OPD
Kenya	14	Male	FB 7-59	OPD
Zaria	8	Male	FB 7-59	Hospital
Kenya	11	female	FB 7-59	OPD
Zaria	14	female	FB 7-59	Hospital
Ile Ife	6	Male	fb0-6	OPD
Zaria	3	Male	fb0-6	Hospital
Zaria	1	female	fb0-6	Hospital
Kenya	11	Male	FB 7-59	OPD
Ibadan	20	Male	FB 7-59	Hospital
Ibadan	2	Male	fb0-6	Hospital
Ibadan	2	Male	fb0-6	Hospital
DRC	1	Male	fb0-6	Hospital
Ibadan	18	Male	FB 7-59	Hospital
DRC	4	Male	fb0-6	OPD
Zaria	2	female	fb0-6	Hospital
Zaria	3	Male	fb0-6	Hospital
DRC	5	female	fb0-6	Hospital
Ibadan	44	Male	FB 7-59	Hospital
Ibadan	6	Male	fb0-6	Hospital
DRC	19	female	LCI	Hospital
Kenya	6	Male	fb0-6	Hospital

Kenya	13	female	FB 7-59	OPD
Ibadan	9	Male	FB 7-59	OPD
Zaria	7	Male	FB 7-59	OPD
Zaria	8	Male	FB 7-59	Hospital
Kenya	4	female	fb0-6	OPD
Ibadan	13	female	FB 7-59	OPD
Zaria	12	Male	convulsions	Hospital
Ibadan	21	female	FB 7-59	Hospital
Ibadan	10	Male	convulsions	Hospital
Zaria	14	Male	convulsions	OPD
Zaria	50	Male	FB 7-59	Hospital
Zaria	8	female	FB 7-59	Hospital
DRC	1	Male	no movement	Hospital
Zaria	1	female	Hypo	OPD
Zaria	1	female	convulsions	OPD
Kenya	10	Male	not feeding	OPD
Kenya	51	female	FB 7-59	Hospital
Zaria	3	Male	fb0-6	Hospital
Zaria	3	female	fb0-6	Hospital
Ibadan	22	Male	convulsions	Hospital
Ibadan	14	Male	convulsions	Hospital
Zaria	10	female	FB 7-59	Hospital
Ibadan	4	female	convulsions	Hospital
Zaria	6	Male	Hypo	Hospital
Zaria	38	female	FB 7-59	Hospital
Ibadan	21	Male	FB 7-59	Hospital
Zaria	3	female	fb0-6	Hospital
Ibadan	14	Male	FB 7-59	OPD
Ibadan	12	female	FB 7-59	Hospital
Zaria	8	Male	FB 7-59	Hospital
Zaria	4	Male	fb0-6	Hospital
Ile Ife	3	Male	LCI	Hospital
Ibadan	35	female	FB 7-59	Hospital
DRC	2	female	>1 CSI	Hospital
Zaria	14	female	FB 7-59	Hospital
Ibadan	7	Male	FB 7-59	Hospital
DRC	1	Male	fb0-6	Hospital
DRC	1	female	fb0-6	OPD
Kenya	6	female	fb0-6	OPD
Zaria	31	female	FB 7-59	Hospital
Ibadan	21	Male	fever	Hospital
Zaria	33	female	FB 7-59	Hospital
Zaria	6	female	fb0-6	Hospital
Ibadan	14	Male	FB 7-59	Hospital
Ibadan	41	female	LCI	Hospital
Zaria	7	Male	FB 7-59	Hospital
DRC	20	Male	not feeding	Hospital
DRC	2	female	fb0-6	Hospital
Zaria	17	Male	FB 7-59	Hospital
Zaria	1	Male	Hypo	OPD

Zaria	11	Male	FB 7-59	Hospital
Zaria	3	Male	poor feeding	Hospital
Zaria	1	female	fb0-6	Hospital
Zaria	20	female	FB 7-59	Hospital
DRC	52	Male	fever	Hospital
DRC	21	female	convulsions	OPD
DRC	7	Male	FB 7-59	OPD
Zaria	2	female	fb0-6	Hospital
Kenya	29	female	convulsions	OPD
Ibadan	6	Male	fb0-6	Hospital
DRC	34	Male	FB 7-59	OPD
Zaria	21	female	Hypo	OPD
Kenya	5	female	fever	OPD
Zaria	35	Male	FB 7-59	Hospital
Kenya	1	female	Hypo	OPD
DRC	16	female	poor feeding	OPD
Zaria	6	Male	fb0-6	Hospital
Zaria	1	female	fb0-6	Hospital
Zaria	4	Male	fb0-6	Hospital
Zaria	8	Male	FB 7-59	Hospital
Zaria	1	female	Hypo	OPD
Kenya	17	female	FB 7-59	OPD
Ibadan	42	female	poor feeding	Hospital
Zaria	3	Male	fb0-6	Hospital
Zaria	2	Male	convulsions	OPD
DRC	2	Male	Hypo	Hospital
Zaria	6	Male	fb0-6	Hospital
Kenya	12	Male	no movement	OPD
Kenya	18	female	FB 7-59	OPD
Ibadan	20	Male	FB 7-59	Hospital
Kenya	10	female	FB 7-59	OPD
Zaria	21	Male	>1 CSI	Hospital
DRC	6	female	fb0-6	OPD
Kenya	1	Male	Hypo	Hospital
Kenya	6	female	fb0-6	OPD
DRC	4	female	fb0-6	Hospital
Zaria	2	Male	fb0-6	OPD
Ibadan	14	Male	convulsions	Hospital
Zaria	10	Male	FB 7-59	Hospital
DRC	6	Male	fb0-6	OPD
Zaria	14	female	LCI	OPD
DRC	23	female	fever	OPD
Kenya	11	female	FB 7-59	OPD
DRC	6	Male	any CI	OPD
Zaria	7	Male	FB 7-59	OPD
Zaria	1	Male	fb0-6	Hospital
DRC	3	Male	poor feeding	OPD
Kenya	21	Male	FB 7-59	OPD
Ibadan	3	female	fb0-6	Hospital
Kenya	13	Male	not feeding	OPD

Kenya	3	female	Hypo	OPD
Kenya	3	female	Hypo	OPD
Zaria	2	Male	poor feeding	OPD
Kenya	25	female	FB 7-59	OPD
Kenya	23	Male	FB 7-59	OPD
Ibadan	6	Male	fb0-6	Hospital
Kenya	8	female	FB 7-59	OPD
DRC	6	Male	fb0-6	OPD
Kenya	10	female	FB 7-59	OPD
Ibadan	7	Male	FB 7-59	OPD
Kenya	7	Male	FB 7-59	OPD
Kenya	29	female	FB 7-59	Hospital
Kenya	11	female	poor feeding	OPD
Kenya	50	Male	convulsions	OPD
Zaria	21	Male	FB 7-59	Hospital
Zaria	9	Male	FB 7-59	Hospital
Ibadan	6	female	fb0-6	Hospital
DRC	2	female	fb0-6	OPD
Zaria	7	Male	FB 7-59	Hospital
Kenya	6	female	fb0-6	OPD
Ibadan	21	Male	FB 7-59	Hospital
Zaria	19	female	FB 7-59	Hospital
Zaria	5	Male	not feeding	Hospital
Ile Ife	3	female	fever	OPD
Zaria	22	Male	FB 7-59	Hospital
Kenya	5	Male	not feeding	OPD
Zaria	9	female	FB 7-59	Hospital
DRC	2	Male	poor feeding	Hospital
DRC	18	female	FB 7-59	Hospital
Zaria	14	Male	FB 7-59	Hospital
Ibadan	2	Male	fb0-6	Hospital
Zaria	10	female	FB 7-59	Hospital
Ile Ife	6	female	LCI	Hospital
Zaria	3	Male	fb0-6	Hospital
Ibadan	14	female	fever	Hospital
DRC	11	Male	not feeding	OPD
Zaria	37	Male	FB 7-59	Hospital
DRC	6	female	fb0-6	OPD
Kenya	10	Male	FB 7-59	Hospital
DRC	9	female	Hypo	OPD
Zaria	10	Male	FB 7-59	Hospital
Kenya	44	Male	LCI	Hospital
Ile Ife	5	Male	fever	OPD
Kenya	6	Male	fb0-6	OPD
Zaria	7	Male	FB 7-59	Hospital
Ibadan	39	female	FB 7-59	Hospital
Zaria	11	female	FB 7-59	Hospital
DRC	3	Male	convulsions	OPD
DRC	6	Male	fb0-6	OPD
Kenya	8	Male	FB 7-59	OPD

DRC	36	female	>1 CSI	OPD
Zaria	1	Male	convulsions	Hospital
Ile Ife	1	female	LCI	Hospital
Zaria	1	Male	fb0-6	Hospital
Zaria	9	Male	FB 7-59	Hospital
Kenya	2	Male	fb0-6	Hospital
Zaria	2	female	fb0-6	Hospital
Zaria	3	Male	fb0-6	Hospital
DRC	2	Male	fb0-6	OPD
Zaria	1	Male	convulsions	OPD
Ibadan	2	Male	convulsions	Hospital
Zaria	7	Male	FB 7-59	Hospital
Zaria	7	Male	FB 7-59	Hospital
Zaria	3	Male	fb0-6	Hospital
DRC	5	Male	fb0-6	Hospital
Zaria	21	female	FB 7-59	Hospital
Zaria	5	female	fb0-6	Hospital
Zaria	4	female	fb0-6	Hospital
DRC	3	female	convulsions	OPD
Zaria	17	female	fever	OPD
Ibadan	50	Male	>1 CSI	Hospital
Zaria	9	female	FB 7-59	Hospital
Zaria	4	female	fb0-6	Hospital
Kenya	9	Male	poor feeding	OPD
Zaria	21	Male	FB 7-59	Hospital
Zaria	3	Male	Hypo	OPD
Ibadan	2	Male	convulsions	Hospital
DRC	3	Male	convulsions	Hospital
Kenya	16	female	FB 7-59	OPD
DRC	2	female	fb0-6	Hospital
Ile Ife	4	Male	LCI	Hospital
Zaria	2	Male	fb0-6	Hospital
Zaria	3	female	fb0-6	Hospital
Ibadan	15	female	convulsions	Hospital
Ibadan	3	Male	fb0-6	Hospital
DRC	2	female	Hypo	Hospital
Kenya	49	female	fever	OPD
Ibadan	4	Male	fb0-6	Hospital
Zaria	7	Male	FB 7-59	Hospital
Kenya	3	Male	any CI	Hospital
Ibadan	50	Male	FB 7-59	Hospital
Kenya	16	Male	FB 7-59	OPD
Kenya	7	female	no movement	OPD
Ibadan	8	female	FB 7-59	Hospital
Ibadan	3	Male	fb0-6	Hospital
DRC	2	Male	fb0-6	Hospital
Ibadan	1	Male	fb0-6	Hospital
DRC	3	Male	Hypo	Hospital
DRC	2	Male	fb0-6	Hospital
DRC	5	Male	fb0-6	OPD

DRC	9	female	FB 7-59	OPD
Zaria	14	Male	FB 7-59	Hospital
Zaria	7	Male	FB 7-59	Hospital
Zaria	10	Male	FB 7-59	Hospital
Zaria	1	female	fb0-6	Hospital
Zaria	1	Male	fever	Hospital
Zaria	1	female	fb0-6	Hospital
DRC	16	female	FB 7-59	OPD
DRC	8	Male	not feeding	OPD
Ile Ife	6	Male	LCI	Hospital
DRC	25	female	fever	Hospital
Zaria	14	Male	FB 7-59	Hospital
Zaria	6	Male	fb0-6	Hospital
Zaria	19	Male	FB 7-59	Hospital
Zaria	7	Male	FB 7-59	Hospital
Ibadan	11	Male	fever	Hospital
Zaria	3	Male	fb0-6	Hospital
Ibadan	2	female	convulsions	Hospital
Ile Ife	4	female	fb0-6	Hospital
Zaria	2	female	>1 CSI	Hospital
Ile Ife	10	Male	FB 7-59	OPD
DRC	6	Male	fb0-6	OPD
Ibadan	6	female	fb0-6	Hospital
Zaria	9	Male	FB 7-59	Hospital
DRC	17	Male	convulsions	OPD
Zaria	1	Male	fb0-6	Hospital
DRC	6	Male	fb0-6	OPD
DRC	1	Male	>1 CSI	OPD
Ibadan	42	Male	FB 7-59	Hospital
Zaria	14	Male	fever	OPD
Ibadan	2	female	fb0-6	Hospital
Kenya	3	female	Hypo	OPD
Zaria	3	Male	fb0-6	Hospital
Ile Ife	6	female	fb0-6	Hospital
Ile Ife	21	Male	FB 7-59	OPD
Zaria	24	Male	FB 7-59	Hospital
DRC	4	Male	fever	OPD
Zaria	12	female	FB 7-59	OPD
Ibadan	17	Male	FB 7-59	Hospital
Ile Ife	8	female	FB 7-59	OPD
Zaria	6	female	convulsions	OPD
Zaria	3	Male	fb0-6	Hospital
Ibadan	7	Male	FB 7-59	OPD
Ibadan	6	Male	fb0-6	OPD
Zaria	26	Male	FB 7-59	Hospital
Zaria	3	female	fb0-6	Hospital
Ibadan	8	Male	FB 7-59	Hospital
DRC	9	female	FB 7-59	OPD
Ibadan	2	Male	any CI	Hospital
Kenya	10	Male	FB 7-59	OPD

Zaria	21	female	FB 7-59	OPD
Kenya	20	Male	FB 7-59	OPD
Zaria	10	female	FB 7-59	Hospital
Kenya	18	Male	FB 7-59	OPD
Ibadan	6	female	fb0-6	Hospital
Kenya	1	Male	Hypo	Hospital
Kenya	2	Male	fb0-6	OPD
Zaria	15	female	FB 7-59	Hospital
Zaria	3	Male	fb0-6	Hospital
Zaria	6	Male	fb0-6	Hospital
DRC	24	Male	FB 7-59	OPD
Ibadan	7	Male	FB 7-59	OPD
Ibadan	17	female	FB 7-59	Hospital
Kenya	12	Male	Hypo	OPD
Zaria	7	female	FB 7-59	Hospital
DRC	6	Male	fb0-6	Hospital
Ibadan	1	Male	fb0-6	Hospital
Zaria	3	Male	fb0-6	Hospital
Zaria	4	Male	poor feeding	OPD
DRC	26	female	FB 7-59	OPD
DRC	13	Male	FB 7-59	OPD
Ibadan	3	Male	fb0-6	Hospital
DRC	2	Male	fb0-6	Hospital
Ibadan	4	Male	fb0-6	Hospital
DRC	11	female	FB 7-59	Hospital
Zaria	7	female	FB 7-59	Hospital
Ile Ife	4	Male	fever	Hospital
Ibadan	44	Male	FB 7-59	Hospital
Zaria	1	Male	fb0-6	OPD
DRC	46	Male	FB 7-59	Hospital
