# Supplementary material for: Clinical signs of possible serious infection and associated mortality among young infants presenting at first-level health facilities
Source: PLoS One. 2021 Jun 30;16(6):e0253110. doi: 10.1371/journal.pone.0253110 (PMC8244884; doi:10.1371/journal.pone.0253110)
Supplement: Attachment — Submitted filename: Response to reviewers_26 Feb 2021.docx [file pone.0253110.s003.docx]

**Point by point response to reviewers’ comments**

**PONE-D-20-1717: Clinical signs of possible serious infection and associated mortality among young infants presenting at first-level health facilities**

**Reviewer #1**

**Comment#1**

Introduction: “Unfortunately, in many low-resource settings families may not accept referral advice, and sick young infants are not taken to hospital (9-12).” Is refusal by families the major reason for which young infants are not admitted or are there any further barriers to be considered?

**Authors’ response**

Thank you. We agree with the reviewer and have added the following sentence in the revised manuscript:

*“**Common barriers for not accepting referral are distance to the hospital, availability of transport and other logistical reasons such as lack of child care, cost of travel and treatment, lack of permission from husband or family elders, concerns around quality of care, a previous negative experience or poor attitudes of health workers at the hospital, cultural or religious beliefs and the absence of referral protocols/algorithms.”*

**Comment#2**

Methods: „For this analysis, 7129 young infants classified as pneumonia, severe pneumonia, clinical severe infection, or critical illness by IMCI trained nurses at first-level health facilities were selected“ Patients were selected in the outpatient setting and IMCI nurses were trained at first-level health facilities, correct? This sentence is somewhat confusing and the setting of patient inclusion should be better specified.

**Authors’ response**

Thank you for raising this confusion. We have modified the paragraph in the revised manuscript as given below:

*“Briefly, young infants with any sign of infection were identified by trained community-level health workers (CLHW) in the community and were referred to first-level health facilities in the study catchment area for further management. At the first-level health facilities, nurses trained in IMCI assessed infants for signs of possible serious infection. All young infants with any sign of possible serious infection were referred to a hospital. Those infants whose caregivers accepted referral to a hospital were followed up on day 15 of the initial assessment to collect information on their survival status. Those whose caregivers refused referral were offered simplified antibiotic treatment on an outpatient basis at the first-level health facility after consent was obtained and followed until day 15 to collect their survival status. For this analysis, 7129 young infants who presented at the first-level health facilities in the study catchment area and were classified by nurses as pneumonia, severe pneumonia, clinical severe infection, or critical illness were selected.”*

**Comment#3**

Methods – Statistical analyses: Please be more specific on the analyses conducted, e.g. which tests were used for between-group comparisons? How was missing data handled? Were infants that were initially treated in the outpatient and later admitted to an inpatient facility excluded or counted as in- or outpatient treated cases?

**Authors’ response**

We agree with the reviewer and have added the following sentences in the revised manuscript.

*“A Chi-square test was used to compare the CFR between outpatient and inpatient treatment groups and p-values were reported. Cases with missing values were excluded from the analysis. Infants were classified as outpatient or hospital inpatient treatment group based on the initial treatment they received.”*

**Comment#4**

Results: “A total of 18420 young infants 0-59 days old were assessed by IMCI-trained nurses at first-level health facilities at five study sites.” Again this may be misleading in terms of setting of patient screening (community vs first-level health facilities).

**Authors’ response**

Please see our response to the above-mentioned Comment#2, in which we have clarified this issue. We have also modified the sentence in the results section as “*A total of 18420 young infants 0-59 days old identified by the CLHWs in the community were assessed by IMCI-trained nurses at the first-level health facilities at five study sites.”*

**Comment#5**

Results: How was the age and sex distribution of young infants with signs of PSBI? Did the authors collect any data on underlying comorbidity? E.g. add a demographics table.

**Authors’ response**

Thank you for your comment. As suggested we have added the following information about the demographics in the text, *“Neonates up to 28 days were 6040 (84.7%), mean age was 14.5 days (SD 13.6), the median age was 10 days (IQR 4-21) and 3893 (54.6%) were males”*. Unfortunately, no information on comorbidities was available. We did not collect information on underlying comorbidity.

**Comment#6**

Results: Did the distribution of signs of PSBI differ between <7, >=7 days old?

**Authors’ response**

We have added a new Table 1 showing clinical signs stratified by age in the revised manuscript as given below:

**Table 1. Distribution of signs of IMCI^*^ classification stratified by age categories in young infants presenting with any sign of possible serious infection (n=7129)**

| **IMCI^*^ classification** | **Age 0-6 days**  **(N=2752)**  **n (%)** | **Age 7-59 days**  **(N=4377)**  **n (%)** |
| --- | --- | --- |
| **Pneumonia** |  |  |
| Fast breathing^†^ in 7-59 days old^‡^ | Not applicable | 1866 (42.6%) |
| **Severe pneumonia** |  |  |
| Fast breathing^†^ in 0-6 days old^‡^ | 1317 (47.9%) | Not applicable |
| **Clinical severe infection^§^** |  |  |
| High body temperature (≥ 38 ^o^C) ^‡^ | 601 (21.8%) | 811 (18.5%) |
| Severe chest indrawing^‡^ | 290 (10.5%) | 1041 (23.8%) |
| Movement only when stimulated^‡^ | 12 (0.4%) | 19 (0.4%) |
| Stopped feeding well^‡^ | 83 (3.0%) | 170 (3.9%) |
| Low body temperature (< 35.5 ^o^C) ^‡^ | 162 (6.9%) | 31 (0.7%) |
| Multiple signs of clinical severe infection^§^ | 187 (6.8%) | 359 (8.2%) |
| **Critical illness^¶^** |  |  |
| Convulsions^‡^ | 62 (2.2%) | 43 (1.0%) |
| Unable to feed at all^‡^ | 14 (0.5%) | 24 (0.5%) |
| No movement at all^‡^ | 8 (0.3%) | 7 (0.2%) |
| Multiple signs of critical illness**^¶^** | 16 (0.6%) | 6 (0.4%) |

*IMCI: Integrated management of childhood illnesses.

^†^ Fast breathing is defined as a respiratory rate of ≥ 60 breaths per minute.

^‡^ Young infants presenting with a single sign.

^§^ Clinical severe infection is defined as the presence of any one of the following signs: severe chest indrawing, high body temperature (≥ 38 ^o^C), low body temperature (< 35.5 ^o^C), stopped feeding well, or movement only when stimulated.

^¶^ Critical illness is defined as the presence of any one of the following signs: convulsions, unable to feed at all, or no movement at all.

**Commnt#7**

Results: „Of the 7129 young infants, fast breathing (in 7-59 days old)“. Another 18% had fast breathing (in <7d old) according to Fig. 2. I think it would be clearer to refer the proportions to the infants at risk, which means the <7, >= 7-day olds or to report a combined proportion of fast breathing.

**Authors’ response**

Thank you for this comment. In the revised manuscript, we are keeping these two as separate entities according to the current IMCI classification where fast breathing in 7-59 days old doesn’t need a hospital referral and recommended treatment is oral amoxicillin at home, while infant with fast breathing in first 6 days of life needs immediate hospital referral for further management. We have added a new table (Table 1) in the revised manuscript, which shows the prevalence of fast breathing by age categories.

**Comment#8**

Results: ”Compared to inpatient treatment, young infants who received outpatient treatment had a CFR 1.5 times lower for pneumonia (p = 0.74), 3.4 times lower for clinical severe infection or severe pneumonia (p < 0.0001) and 1.8 times higher for critical illness (p = 0.097).“ Were there any differences by study site, level of health facility, underlying comorbidity (if such data is available) or age group of infants (<7 days, >=7 days)? I think it would be important looking a bit deeper into this as this finding may have important implications for care.

**Authors’ response**

Thank you for these suggestions.

First, we analyzed the data by the place of treatment and study sites. Please see the table “*Case fatality rate with the place of treatment by study site and by IMCI classification and recategorization of signs of clinical severe infection or severe pneumonia on mortality risk”* reporting that analysis (see below). We noticed the same trend of deaths in the outpatients and among the hospitalized patients, but numbers in some cells are small so it is difficult to draw any conclusions for study sites. We have reported this as supplementary table S1.

Second, we analyzed the data by the place of treatment and age 0-6 days and >=7 days. Please see the table “*Case fatality rate with the place of treatment by age and by IMCI classification and recategorization of signs of clinical severe infection or severe pneumonia on mortality risk*” reporting that analysis (see below). The CFR was higher for both 0-6 days and 7-59 days age groups among severe pneumonia and clinical severe infection categories in hospitalized versus outpatient treatment groups. In contrast among 0-6 days old infants with a critical illness, the CFR was more than twice in the outpatient treatment group compared to the hospitalized patients. In the 0-6 days old age group, both low and moderate-mortality risk signs had over twice the CFR for those treated in a hospital compared to outpatients. In contrast, CFR was 9 times higher in 7-59 days old with moderate-mortality risk signs amongst those hospitalized, although the numbers are rather small. We have added this as Table 4 to the manuscript.

Third, as mentioned earlier AFRINEST study did collect data on other co-morbidities.

**Table S1. Case fatality rate with the place of treatment by study site and by IMCI* classification and recategorization of signs of clinical severe infection or severe pneumonia on mortality risk (n = 7046)**

|  | **DRC (n=1253)** | | **Kenya (n=2269)** | | **Ibadan, Nigeria (n=1164)** | | **Ile-Ife, Nigeria (n=1039)** | | **Zaria, Nigeria (n=1321)** | |
| --- | --- | --- | --- | --- | --- | --- | --- | --- | --- | --- |
|  | **Outpatient treatment** | **Hospitalized treatment** | **Outpatient treatment** | **Hospitalized treatment** | **Outpatient treatment** | **Hospitalized treatment** | **outpatient treatment** | **Hospitalized treatment** | **Outpatient treatment** | **Hospitalized treatment** |
|  | **Deaths/ infants (CFR**^†^**)** | **Deaths/ infants (CFR**^†^**)** | **Deaths/ infants (CFR**^†^**)** | **Deaths/ infants (CFR**^†^**)** | **Deaths/ infants (CFR**^†^**)** | **Deaths/ infants (CFR**^†^**)** | **Deaths/ infants (CFR**^†^**)** | **Deaths/ infants (CFR**^†^**)** | **Deaths/ infants (CFR**^†^**)** | **Deaths/ infants (CFR**^†^**)** |
| **A. IMCI *classification** |  |  |  |  |  |  |  |  |  |  |
| Pneumonia^‡^ | 0/284 (0.0%) | 0/8 (0.0%) | 1/711 (0.1%) | 1/11 (9.1%) | 0/168 (0.0%) | 0/64 (0.0%) | 0/173 (0.0%) | 0/0 | 2/165 (1.2%) | 0/259 (0.0%) |
| Clinical severe infection^§^ or severe pneumonia^¶^ | 26/837 (3.1%) | 7/50 (14.0%) | 17/1510 (1.1%) | 2/18 (11.1%) | 7/800 (0.9%) | 2/96 (2.1%) | 4/824 (0.5%) | 1/37 (2.7%) | 34/714 (4.8%) | 11/151 (7.3%) |
| Critical illness^#^ | 11/41 (26.8%) | 8/33 (24.2%) | 3/16 (18.7%) | 2/3 (66.7%) | 1/3 (33.3%) | 0/2 (0.0%) | 0/5 (0.0%) | 0/0 | 3/18 (16.7%) | 0/14 (0.0%) |
|  |  |  |  |  |  |  |  |  |  |  |
| **B. Recategorization of signs of clinical severe infection**^§^ **or severe pneumonia**^¶^ **based on mortality risk** |  |  |  |  |  |  |  |  |  |  |
| **Lower-mortality risk signs^**^** | **12/677 (1.8%)** | **4/31 (12.9%)** | **8/1120 (0.7%)** | **1/13 (7.7%)** | **6/686 (0.9%)** | **0/87 (0.0%)** | **4/775 (0.5%)** | **1/36 (2.8%)** | **7/468 (1.5%)** | **6/136 (4.4%)** |
| High body temperature^††^ | 3/338 (0.9%) | 0/2 (0.0%)) | 1/300 (0.3%) | 0/4 (0.0%)- | 2/339 (0.6%) | 0/11 (0.0%) | 1/202 (0.5%) | 0/6 (0.0%) | 4/204 (2.0%) | 0/3 (0.0%) |
| Severe chest indrawing^††^ | 4/113 (3.5%) | 1/2 (50.0%) | 2/526 (0.4%) | 0/2 (0.0%) | 2/181 (1.1%) | 0/6 (0.0%) | 1/403 (0.2%) | 1/9 (11.1%) | 1/87 (1.1%) | 0/0 |
| Only fast breathing in 0-6 days old^††^ | 5/226 (2.2%) | 3/27 (11.1%) | 5/294 (1.7%) | 1/7 (14.3%) | 2/166 (1.2%) | 0/70 (0.0%) | 2/170 (1.2%) | 0/21 (0.0%) | 2/177 (1.1%) | 6/133 (4.5%) |
| **Moderate-mortality risk signs^‡‡^** | **14/160 (8.7%)** | **3/19 (15.8%)** | **9/390 (2.3%)** | **1/5 (20.0%)** | **1/114 (0.9%)** | **2/9 (22.2%)** | **0/49 (0.0%)** | **0/1 (0.0%)** | **27/246 (11.0%)** | **5/15 (33.3%)** |
| Movement only when stimulated^††^ | 0/3 (0.0%) | 0/0 | 1/6 (16.7%) | 0/0 | 0/9 (0.0%) | 0/1 (0.0%) | 0/0 | 0/0 | 0/12 (0.0%) | 0/0 |
| Stopped feeding well^††^ | 2/38 (5.3%) | 1/3 (33.3%) | 4/87 (4.6%) | 0/0 | 0/32 (0.0%) | 0/3 (0.0%) | 0/4 (0.0%) | 0/0 | 3/82 (3.7%) | 0/2 (0.0%) |
| Low body temperature^††^ | 3/36 (8.3%) | 1/11 (9.1%) | 1/55 (1.8%) | 1/3 (33.3%) | 0/7 (0.0%) | 1/1 (100.0%) | 0/7 (0.0%) | 0/0 | 11/56 (19.6%) | 2/6 (33.3%) |
| Multiple signs of clinical severe infection^§^ | 9/83 (10.8%) | 1/5 (20.0%) | 3/390 (2.3%) | 0/2 (0.0%) | 1/66 (1.5%) | 1/4 (25.0%) | 0/38 (0.0%) | 0/1 (0.0%) | 13/96 (13.5%) | 3/7 (42.9%) |

*IMCI: Integrated management of childhood illnesses.

†CFR: Case fatality rate.

‡Pneumonia is defined as fast breathing (respiratory rate of ≥ 60 breaths per minute ) in 7-59 days old infants.

§Clinical severe infection is defined as the presence of any one of the following signs: severe chest indrawing, high body temperature (≥ 38 ^o^C), stopped feeding well, movement only when stimulated, or low body temperature (< 35.5 ^o^C).

¶ Severe pneumonia is defined as fast breathing (respiratory rate of ≥ 60 breaths per minute) in 0-6 days old infants.

^#^Critical illness is defined as the presence of any one of the following signs: convulsions, unable to feed at all, or no movement at all. For the current analysis, only these three common signs of critical illness were considered.

^**^ Lower mortality risk signs are defined as infants with a case fatality rate for any sign ≤ 2.0%.

†† Young infants presenting with a single sign.

‡‡ Moderate mortality risk signs are defined as infants with a case fatality rate for any sign >2.0%.

**Table 4.** **Case fatality rate with the place of treatment by age and by IMCI^*^ classification and recategorization of signs of clinical severe infection or severe pneumonia on mortality risk (n = 7046)**

|  | **Age 0-6 days**  **(n=2707)** | | **Age 7-59 days**  **(n=4339)** | |
| --- | --- | --- | --- | --- |
|  | **Outpatient treatment** | **Hospitalized treatment** | **Outpatient treatment** | **Hospitalized treatment** |
|  | **Deaths/ infants (CFR^†^)** | **Deaths/ infants (CFR^†^)** | **Deaths/ infants (CFR^†^)** | **Deaths/ infants (CFR^†^)** |
| **A. IMCI* classification** |  |  |  |  |
| Pneumonia^‡^ | Not applicable | Not applicable | 3/1501 (0.2%) | 1/342 (0.3%) |
| Clinical severe infection§ or severe pneumonia^¶^ | 62/2300 (2.7%) | 20/316 (6.3%) | 26/2385 (1.1%) | 3/36 (8.3%) |
| Critical illness^#^ | 12/44 (27.3%) | 6/47 (12.8%) | 6/39 (15.4%) | 4/36 (11.1%) |
|  |  |  |  |  |
| **B. Recategorization of signs of clinical severe infection**^§^ **or severe pneumonia**^¶^ **based on mortality risk** |  |  |  |  |
| **Lower-mortality risk signs^**^** | **27/1902 (1.4%)** | **12/279 (4.3%)** | **10/1824 (0.5%)** | **0/24 (0.0%)** |
| High body temperature^††^ | 8/588 (1.4%) | 0/12 (0.0%)) | 3/795 (0.4%) | 0/14 (0.0%) |
| Severe chest indrawing^††^ | 3/281 (1.1%) | 2/9 (22.2%) | 7/1029 (0.7%) | 0/10 (0.0%) |
| Only fast breathing in 0-6 days old^††^ | 16/1033 (1.5%) | 10/258 (3.9%) | NA | NA |
| **Moderate-mortality risk signs^‡‡^** | **35/398 (8.8%)** | **8/37 (21.6%)** | **16/561 (2.8%)** | **3/12 (25.0%)** |
| Movement only when stimulated^††^ | 1/11 (9.1%) | 0/1 (0.0%) | 0/19 (0.0%) | 0/0 |
| Stopped feeding well^††^ | 5/79 (6.3%) | 1/4 (25.0%) | 4/164 (2.4%) | 0/4 (0.0%) |
| Low body temperature^††^ | 13/134 (9.7%) | 4/19 (21.0%) | 2/27 (7.4%) | 1/1 (50.0%) |
| Multiple signs of clinical severe infection^§^ | 16/174 (9.2%) | 3/13 (23.1%) | 10/351 (2.8%) | 2/6 (33.3%) |

*IMCI: Integrated management of childhood illnesses.

†CFR: Case fatality rate.

‡Pneumonia is defined as fast breathing (respiratory rate of ≥ 60 breaths per minute ) in 7-59 days old infants.

§Clinical severe infection is defined as the presence of any one of the following signs: severe chest indrawing, high body temperature (≥ 38 ^o^C), stopped feeding well, movement only when stimulated, or low body temperature (< 35.5 ^o^C).

¶ Severe pneumonia is defined as fast breathing (respiratory rate of ≥ 60 breaths per minute) in 0-6 days old infants.

^#^Critical illness is defined as the presence of any one of the following signs: convulsions, unable to feed at all, or no movement at all. For the current analysis, only these three common signs of critical illness were considered.

^**^ Lower mortality risk signs are defined as infants with a case fatality rate for any sign ≤ 2.0%.

†† Young infants presenting with a single sign.

‡‡ Moderate mortality risk signs are defined as infants with a case fatality rate for any sign >2.0%.

**Comment#9**

Discussion: „This finding could be due to several reasons, such as delay in reaching the hospital after accepting referral advice, relatively sicker patients accepting referral advice, delay in getting appropriate treatment after reaching the hospital or inadequate quality of care, and development of nosocomial infections (23-25).“

**Comment#9.a.** Are there any other studies from LMIC with similar results?

**Authors’ response**

We could only find one published study by Hibberd et al that reported CFR by some clinical signs.^[1]^ They reported CFR for some individual signs of PSBI in young infants in a multicentre study from low resource settings; 0.8% for high fever, 5.7% for a breathing problem, 15.3% for feeding problem/stopped suckling/feeding, 18% for convulsions and 18.4% for hypothermia. However, they did not calculate it for all signs of infection and did not report it for the place of treatment. Similar data were collected in the two SATT and ANISA studies^[2]^, but we could not find any published or unpublished information from these studies presenting similar analyses.

**Comment#9.b.** Is there a way to adjust for the severity of disease and comorbidities in the data? I think if the authors consider differences in disease severity as a possible explanation of their findings, it would be important to control for this confounder. Otherwise, the results should be presented with more caution and this issue should be more prominently included in the discussion.

**Authors’ response**

Thank you for raising this issue. Unfortunately, AFRINEST study did not collect any information on comorbidities. We have discussed this in study limitations as given below:

*We did not collect information on comorbidities in this study.*

**Comment#9.c.** Were there any other signs detected by the trained nurses that can help to distinguish infants likely to benefit from hospital admissions vs. infants that may have better outcomes if treated in the outpatient setting?

**Authors’ response**

AFRINEST’s objective was to evaluate the effectiveness of simplified antibiotics when the referral to a hospital was not feasible, especially in low resource settings. For this purpose, all the nurses were trained in IMCI to identify sick young infants using signs recommended for referral to a hospital. Data on signs not included in the IMCI algorithm was not actively collected.

**Comment#10**

Discussion: Fourth, infants categorized as having higher mortality risk signs, who are not as sick as critically ill, could benefit from a shorter stay in the hospital when they accept referral instead of the recommended seven days (8).

Based on which data was this conclusion made? Please underpin this hypothesis with data or references.

**Authors’ response**

Thank you for your comment. First, we have corrected ‘higher mortality risk signs’ as moderate-mortality risk signs (typographical mistake), to distinguish them from the critical illness that includes high-mortality risk signs in the revised manuscript. Second, it has been demonstrated in the AFRINEST and SATT studies^[3]^ that most young infants with signs of ‘clinical severe infection’ were cured with outpatient simplified antibiotics regimens. However, there was around 10% treatment failure, which included 3% deaths or clinical deterioration. We hypothesize that these young infants with a ‘clinical severe infection’ who didn’t benefit from simplified antibiotic regimens on an outpatient basis could benefit from hospitalization with a shorter stay, compared to ‘critically ill’ young infants who would need a longer hospitalization.

**Comment#11**

Can any conclusions on the definition of sepsis in LMIC be drawn from this data?

**Authors’ response**

AFRINEST was not designed to define ‘neonatal sepsis’, nor was it designed to identify the aetiology of neonatal sepsis. It was a pragmatic trial to identify how to best treat young infants with ‘possible serious infection’ when a referral to a hospital is not feasible. Therefore the data do not allow us to inform us about improving the definition of ‘sepsis’ in young infants in LMICs.

**Reviewer #2**

**Comment#1**

Abstract page 2: The authors should avoid using abbreviations in the abstract (see Submission Guidelines of the Journal).

**Authors’ response**

In the revised manuscript, we have removed all the abbreviations in the abstract.

**Comment#2**

Introduction: 1) The authors state on page 4 row 1-6: “Neonatal mortality has decreased…for case fatality rate (CFR) of 9.8% (4).” Please try to supply the reader with more recent data about neonatal mortality, incidence of bacterial infections / sepsis and who they are defined!!! Some of the references are from 2009 and 2012.

**Authors’ response**

Thank you for your comment. We have modified the first paragraph in the ‘Introduction’ of the revised manuscript as given below and have updated a couple of references:

*An estimated 2.4 million neonatal deaths occurred globally in 2019, representing 47% of under-five deaths in 2019. The neonatal period in the first 28 days of life after birth is the most vulnerable time for survival. Each day 6700 neonatal deaths occur, one-third on the first day of birth, and three-quarters in the first week of life. The neonatal global rate of 17 deaths per 1000 live births in 2019 reduced by 52% from 37 deaths per 1000 in 1990. Bacterial infections including sepsis, meningitis and pneumonia account for 37% of neonatal deaths in the low-resource settings of South Asia and sub-Saharan Africa. An estimated 9.8% died of the 6.9 million neonatal cases of possible serious bacterial infection (PSBI) in 2012. A recent systematic review of 26 studies from 14 countries estimated the neonatal sepsis incidence as 2824 per 100000 live-births with a mortality of 17.6%. However, the review also highlighted the variations in the definition of sepsis.*

**Comment#3**

There is discrepancy between the objective mentioned in the end of the introduction part of the manuscript (page 5 row 7-10) and the objective mentioned in the abstract in the background (page 2, row 3-4) part. The authors should give us consistent description of the “objective of the study”!!!

**Authors’ response**

We appreciate that you raised this point. We have revised the sentence in the ‘Introduction’ of the revised manuscript as given below:

*Therefore, we conducted an analysis using African Neonatal Sepsis Trial (AFRINEST) data to calculate the case fatality rate (CFR) for infants with individual or combined signs of possible serious infection, stratified by hospitalized or outpatient treatment.*

**Comment#4**

See the following information which should be integrated in your paper.

**Comment#4.a.** Neonatal Mortality ( Reference: data.unicef.org)

The first 28 days of life – the neonatal period – is the most vulnerable time for a child’s survival. Children face the highest risk of dying in their first month of life at an average global rate of 17 deaths per 1,000 live births in 2019, down by 52 per cent from 38 deaths per 1,000 in 1990.

**Comment#4.b.** In comparison, the probability of dying after the first month and before reaching age 1 was estimated at 11 deaths per 1,000 and the probability of dying after reaching age 1 and before reaching age 5 was estimated at 10 deaths per 1,000 in 2019.

**Comment#4.c.** Globally, 2.4 million children died in the first month of life in 2019 – approximately 6,700 neonatal deaths every day – with about a third of all neonatal deaths occurring within the first day after birth, and close to three-quarters occurring within the first week of life.

**Authors’ response:**

Thank you for your comments 4a-4c. We have modified the first paragraph in the ‘Introduction section’ as follows:

*An estimated 2.4 million neonatal deaths occurred globally in 2019, representing 47% of under-five deaths in 2019. The neonatal period in the first 28 days of life after birth is the most vulnerable time for survival. Each day 6700 neonatal deaths occur, one-third on the first day of birth, and three-quarters in the first week of life. The neonatal global rate of 17 deaths per 1000 live births in 2019 reduced by 52% from 37 deaths per 1000 in 1990. Bacterial infections including sepsis, meningitis and pneumonia account for 37% of neonatal deaths in the low-resource settings of South Asia and sub-Saharan Africa. An estimated 9.8% died of the 6.9 million neonatal cases of possible serious bacterial infection (PSBI) in 2012. A recent systematic review of 26 studies from 14 countries estimated the neonatal sepsis incidence as 2824 per 100000 live-births with a mortality of 17.6%. However, the review also highlighted the variations in the definition of sepsis.*

**Comment#4.d.** Definitions of indicators: Neonatal mortality rate: Probability of dying during the first 28 days of life, expressed per 1,000 live births. Infant mortality rate: Probability of dying between birth and exactly 1 year of age, expressed per 1,000 live births. Under-five mortality rate: Probability of dying between birth and exactly 5 years of age, expressed per 1,000 live births.

**Authors’ response**

We have incorporated the neonatal mortality rate in the text of the first paragraph of the ‘Introduction section’, as our manuscript is addressing infants up to two months of age.

**Comment#5**

Methods: The authors should explain why they defined outcome (“survival status”) on “day 15 after the initial assessment” and not in the end of the neonatal period day 28?

**Authors’ response**

This is a secondary analysis of AFRINEST dataset. That trial was planned to assess the outcome in sick young infants up to two months of age with possible serious infection till day 15 after enrollment. The probable justification was that an adverse outcome could occur up to that time after treatment and that it would be feasible to follow the enrolled infant up to 15 days.

**Comment#6**

Page 6 row 18-21: “CFR, was defined at the…..alone or in combination” This description of CFR definition should be better placed in the Methods part of the manuscript but not in the section of “statistical analysis”.

**Authors’ response**

We have included the definition of CFR in the ‘Outcomes’ in the ‘Methods section’. We have further explained the detail in statistical analysis.

**Comment#7**

Results: The authors should decide how they want to present their results. It is unnecessary to present the same information in the text with a written description and a Figure which presents the same information. Therefore, I would suggest to omit Figure 1 from the manuscript.

**Authors’ response**

As suggested by the reviewer we have omitted Figure 1 from the revised manuscript.

**Comment#8**

The authors mentioned that 7086 children were included in the study. But on page 7, row 17; “Of the 7192 young infants….” Please clarify this discrepancy in the numbers of patients.

**Authors’ response**

Eighty-three out of 7129 young infants with any sign of possible serious infection were excluded from analysis due to non-availability of information on survival status, thus 7046 young infants data were analysed. We have modified the following sentence in the revised manuscript:

*However, 83 young infants, for whom information on survival status was missing, were excluded from the analysis, thus data for 7046 young infants were analysed.*

**Comment#9**

The authors should decide once again, how they want to present their results. It is unnecessary to present the same information in the text with a written description (page 7, row 21-23 and page 8, row 1-4) and a table (Table 1) which presents the same information. In this case I would suggest to leave Table 1 which supplies well-arranged information and only “refer the reader” to this table in the results part without presenting the same result in written way.

**Authors’ response**

Thank you for highlighting this issue. We have modified the text in the result section in the revised manuscript as advised by the reviewer.

**Comment#10**

Discussion: The authors state on page 9, row 1-2 that there was a higher CFR in outpatients compared to inpatients with the same signs of critical illness this should be more in detail elaborated.

**Authors’ response**

In the first paragraph under ‘Discussion’, we highlighted the main findings. We elaborated on this aspect further in the paragraph under ‘Research and policy implications’ – “First, infants with any sign of critical illness cannot be managed at an outpatient level because they not only require antibiotics, but also other supportive care such as oxygen, fluid therapy, nutrition, management of hypothermia and hypoglycaemia.”

1. Hibberd PL, Hansen NI, Wang ME, Goudar SS, Pasha O, Esamai F, et al. Trends in the incidence of possible severe bacterial infection and case fatality rates in rural communities in Sub-Saharan Africa, South Asia and Latin America, 2010–2013: a multicenter prospective cohort study. Reprod Health 2016; 13:65.
2. Baqui A, Saha S, Ahmed A, Shahidulla M, Quasem I, Roth DE et al. Safety and efficacy of alternative antibiotic regimens compared with 7 day injectable procaine benzylpenicillin and gentamicin for outpatient treatment of neonates and young infants with clinical signs of severe infection when referral is not possible: a randomised open label, equivalence trial. Lancet Glob Heal. 2015; 3: e279–87.

   Mir F, Nisar I, Tikmani SS, Baloch B, Shakoor S, Jehan F, Ahmed I, Cousens S, Zaidi AK. Simplified antibiotic regimens for treatment of clinical severe infection in the outpatient setting when referral is not possible for young infants in Pakistan (Simplified Antibiotic Therapy Trial [SATT]): a randomised, open-label, equivalence trial. Lancet Glob Health. 2017; 5: e177-e185.

   Saha SK, Schrag SJ, El Arifeen S, Mullany LC, Shahidul Islam M, Shang N et al. Causes and incidence of community-acquired serious infections among young children in south Asia (ANISA): an observational cohort study. Lancet. 2018;392:145-159.
3. Baqui A, Saha S, Ahmed A, Shahidulla M, Quasem I, Roth DE et al. Safety and efficacy of alternative antibiotic regimens compared with 7 day injectable procaine benzylpenicillin and gentamicin for outpatient treatment of neonates and young infants with clinical signs of severe infection when referral is not possible: a randomised open label, equivalence trial. Lancet Glob Heal. 2015; 3: e279–87.

   Mir F, Nisar I, Tikmani SS, Baloch B, Shakoor S, Jehan F, Ahmed I, Cousens S, Zaidi AK. Simplified antibiotic regimens for treatment of clinical severe infection in the outpatient setting when referral is not possible for young infants in Pakistan (Simplified Antibiotic Therapy Trial [SATT]): a randomised, open-label, equivalence trial. Lancet Glob Health. 2017; 5: e177-e185.

   Tshefu A, Lokangaka A, Ngaima S, Engman C, Esamai F et al and African Neonatal Sepsis Trial (AFRINEST) Group. Simplified antibiotic regimens compared with injectable procaine benzylpenicillin plus gentamicin for treatment of neonates and young Infants with clinical signs of severe infection when referral is not possible: a randomized equivalence trial. Lancet. 2015; 385: 1767–76
